# Photocorrosion, Protection, and Predictive Design in Solar Water Splitting

**DOI:** 10.1002/smll.75177

**Published:** 2026-08-27

**Authors:** Seung Wook Shin, Girish U. Kamble, Uma V. Ghorpade, Dong Hyun Kang, Youseong Park, Mahesh P. Suryawanshi, Keeyoung Jung, Donghoon Song, Jin Hyeok Kim

**Affiliations:** ^1^ Rural Research Institute Korea Rural Community Corporation Ansan Republic of Korea; ^2^ Optoelectronics Convergence Research Center and Department of Materials Science and Engineering Chonnam National University Gwangju Republic of Korea; ^3^ School of Chemical Engineering UNSW Sydney Sydney Australia; ^4^ School of Photovoltaic and Renewable Energy Engineering UNSW Sydney Sydney Australia; ^5^ Department of Energy Science and Engineering (ESE) Sunchon National University Suncheon Republic of Korea; ^6^ Institute for Battery Research (IBR) Sunchon National University Suncheon Republic of Korea; ^7^ Department of Chemical Engineering Sunchon National University Suncheon Republic of Korea

**Keywords:** characterization methods, computing strategies, interfacial engineering, long‐term stability, photocorrosion, PEC water splitting devices, surface defects

## Abstract

Photoelectrochemical (PEC) technology is a promising pathway for cost‐effective, renewable, and clean fuel production from sunlight and water, addressing critical global energy demands. Despite extensive efforts, the major challenges, including unsatisfactory solar‐to‐hydrogen (STH) efficiency of less than 11.2% and limited long‐term stability of less than 3000 h, for the commercialization of PEC water‐splitting devices remain unresolved. To strengthen PEC performance and facilitate commercialization, we provide a comprehensive review of key fundamental mechanisms, recent advances in functional materials, interfacial engineering strategies, advanced characterization methods, and computational strategies. These aspects are interrelated and can synergistically form a feedback loop. In particular, computing strategies, which involve artificial intelligence (AI) for predicting device performance and automating experimental processes, can enable circulation within the feedback loop and hence accelerate the advancement of PEC water‐splitting devices toward commercial viability (STH efficiency: >15% and long‐term stability: >5 years). Ultimately, we aim to inspire researchers in this community through this comprehensive approach to the commercial production of green hydrogen applicable to diverse industrial sectors.

## Graphical abstract

This Review outlines a roadmap toward highly efficient and durable photoelectrochemical water splitting by integrating fundamental understanding, advanced materials engineering, and AI‐driven digital discovery strategies. Recent progress in interfacial design, stability enhancement, operando characterization, and automated materials optimization is highlighted to accelerate the development of commercially viable solar hydrogen technologies.

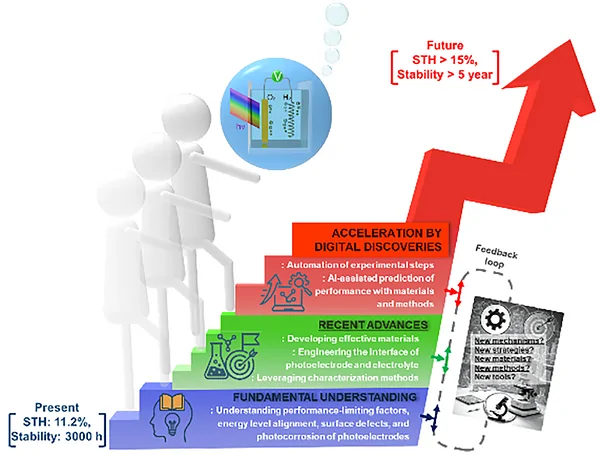

**Abbreviations**
PEC: Photoelectrochemical
STH: Solar‐to‐hydrogen
OER: Oxygen evolution reaction
HER: Hydrogen evolution reaction
RHE: Reversible hydrogen electrode
NHE: Normal hydrogen electrode
FE: Faradaic efficiency
ABPE: Applied‐bias photon‐to‐current efficiency
HTL: Hole transport layer
ETL: Electron transport layer
SAM: Self‐assembled monolayer
TAS: Transient absorption spectroscopy
TPC/TPV: Transient photocurrent/transient photovoltage
XPS: X‐ray photoelectron spectroscopy
XAS: X‐ray absorption spectroscopy
CVD: Chemical vapor deposition
ALD: Atomic layer deposition
AI: Artificial Intelligence
ML: Machine Learning
DL: Deep Learning
PV: Photovoltaic
CB: Conduction Band
VB: Valence Band
CBM: Conduction Band Minimum
VBM: Valence Band Maximum
IPCE: Incident Photon‐to‐Current Efficiency
XPS: X‐ray Photoelectron Spectroscopy
XAS: X‐ray Absorption Spectroscopy
XANES: X‐ray Absorption Near‐Edge Structure
EXAFS: Extended X‐ray Absorption Fine Structure
SPM: Scanning Probe Microscopy
SECM: Scanning Electrochemical Microscopy
UME: Ultramicroelectrode
MIS: Metal Insulator Semiconductor
BHJ: Bulk Heterojunction
LDH: Layered Double Hydroxide
OEC: Oxygen Evolution Catalyst
TRMC: Time‐Resolved Microwave Conductivity
TPC: Transient Photocurrent
TPV: Transient Photovoltage
TAS: Transient Absorption Spectroscopy
PAAM: Polyacrylamide
QDs: Quantum Dots
NRs: Nanorods
PEIS: Potential‐Dependent Electrochemical Impedance Spectroscopy
CVD: Chemical Vapor Deposition
MBE: Molecular Beam Epitaxy

## 1 Introduction

Leveraging collective efforts in fundamentals, recent advances, and digital discovery strategies facilitates the development of photoelectrochemical (PEC) water‐splitting systems, which convert renewable energy into clean fuel (i.e., hydrogen (H_2_)), thereby helping to mitigate the global energy crisis, reduce environmental pollution, and address concerns over global warming [1]. In addition to its use as a solar fuel, H_2_ is an essential resource for diverse sectors, including fertilizer production, polymer feedstock, oil refining, steelmaking, and electronics, among others [2]. Normally, the PEC water splitting system is composed of a photoanode, an electrolyte, and a photocathode, with a membrane included as needed, and utilizes sunlight to electrochemically split water into molecular oxygen (O_2_) and H_2_ at each electrode. While the oxygen evolution reaction (OER) occurs at the photoanode and the hydrogen evolution reaction (HER) at the photocathode, the former constitutes the performance‐limiting step in PEC systems due to poor charge transfer and/or high recombination rates at each interface [3, 4, 5, 6, 7]. Thus, promoting the OER to achieve high solar‐to‐hydrogen (STH) efficiency is key to commercial deployment, as concurrent with excellent long‐term stability [8]. Specifically, the target STH efficiency of 15% and long‐term stability of 5 years are minimum targets for commercialization stage [3]. To fulfill these achievements, various inorganic compound‐based photoelectrodes, including Si, III‐V based compounds, and emerging materials (i.e., Cu_2_O, CZTSSe, Sb_2_(S, Se)_3_, TiO_2_, Fe_2_O_3_, and BiVO_4_) have been developed to date. In particular, concurrently high STH conversion efficiency and high long‐term stability of photoelectrodes have been realized using a few semiconductors like Si and III‐V based compounds [9]. High STH efficiency is achievable with many semiconducting materials [3]. With perovskite‐based compounds, present‐day high STH efficiency and long‐term stability reach 11.2% and 3000 h, [10, 11] respectively, especially revealing that the long‐term stability is far below the minimum. Achieving the target performance is therefore closely associated with greatly extending the long‐term stability. In this review, an STH efficiency of ≥10% is regarded as a research‐level benchmark demonstrating practical PEC feasibility, whereas commercialization‐oriented PEC systems are generally expected to achieve STH efficiencies >15% together with operational stability exceeding 5 years to satisfy economic and deployment requirements [12].

PEC devices are subjected to harsh electrolyte conditions (i.e., strong acid or basic pH) [4, 12]. It is well known that the surface of the photoelectrode in the PEC water‐splitting device is always in direct contact with the electrolyte, leading to a significantly rapid degradation. The chemical corrosion of photoelectrodes is strongly dependent on PEC device structures and performance measurement conditions, such as the intensity of light illumination, biasing voltage, catalyst, surface passivation (i.e., overlayer on photoelectrode), the electronic band structure of each layer in photoelectrodes, compositional ratio, and pH in electrolyte, semiconductor/electrolyte and catalysis/electrolyte interfaces [13]. Countless efforts have been made to tackle the photocorrosion issue through introducing complex architectures for PEC water‐splitting devices, which can integrate photoelectrodes with a suitable cocatalyst, passivation, and/or tunneling junction layers [14]. It should be noted that deposition methodologies strongly affect the quality of these layers.

Despite the aforementioned efforts on materials, photoelectrode or electrolyte engineering strategies, the trade‐off relationship between PEC efficiency and long‐term stability is found in many cases [15, 16]. Furthermore, the fundamental mechanisms have not been fully unraveled, which limits the design space of PEC devices and hinders their progress. To date, diverse sets of characterization methods enabling reaction dynamic analysis and in situ analysis have been developed for PEC devices [17, 18, 19, 20]. It should be emphasized that the characterization methods can be exploited to unravel fundamental mechanisms on various materials and strategies. Meanwhile, digital discovery strategies, including artificial intelligence (AI) and deep learning (DL), and machine learning (ML), [21, 22, 23] are gaining prominence across various research disciplines. With particular relevance to PEC systems, digital discovery strategies can significantly help reveal effective design principles or key factors through bibliometric approaches [24, 25, 26, 27, 28, 29, 30, 31], which are complementary to characterization methods. In addition, they contribute to discovering promising materials for catalysts, passivation layers, and photoelectrodes [32, 33], predicting STH conversion efficiency and long‐term stability [34, 35, 36], and enabling automated experimentation [37, 38]. Automation can especially incorporate characterization methods and data collection and analysis, along with processing steps involving functional materials. These advantages curtail significant time and experimental input from researchers and hence enable the accelerated development of effective materials and strategies for PEC devices.

**FIGURE 1:**
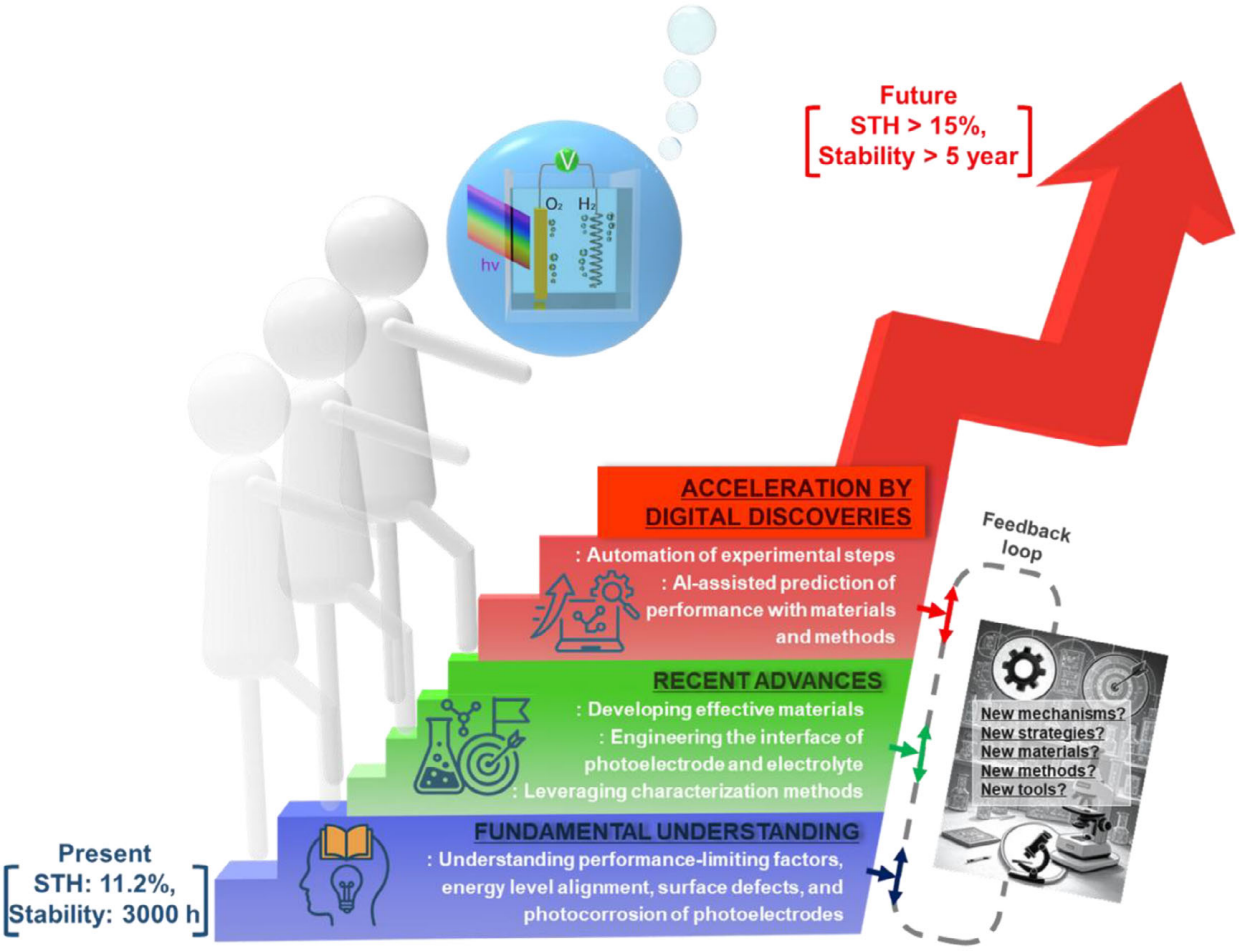
A graphic representing the topics and issues addressed in this review.

This review differs from prior PEC surveys by unifying fundamental mechanisms, materials development, interfacial engineering, advanced characterization, and emerging AI/ML‐driven discovery strategies within a single framework aimed at accelerating PEC commercialization. Particular emphasis is placed on feedback loops between experiment, computation, and operando characterization, and on the simultaneous optimization of efficiency and long‐term stability. While the overall strategy for commercialization of PEC water‐splitting devices is depicted in Figure 1, we herein specifically focus on the fundamental mechanisms of key performance‐limiting factors, including energy level alignment, surface defects, and photocorrosion of photoelectrodes in PEC water‐splitting devices (Section 2). We then move on to recently explored photoelectrode materials (Section 3) and interfacial engineering strategies (Section 4), after which we proceed with fundamental characterization methods (Section 5) and computing strategies (Section 6). As the aforementioned key sections on fundamentals, recent advances, and computing strategies are intensely interrelated and hence synergistic, the commercialization of PEC devices can be facilitated in the near future.

In assembling this review, literature was surveyed primarily through Web of Science, Scopus, and Google Scholar using keywords such as “photoelectrochemical water splitting”, “photoelectrode stability”, “solar‐to‐hydrogen efficiency”, “photocorrosion”, “interface engineering”, and “photoelectrochemistry machine learning”. Emphasis was placed on recent work from the past 10 years in high‐impact journals, including reports with state‐of‐the‐art efficiency or stability and mechanistic studies that clarify structure‐performance‐stability relationships, while seminal earlier contributions are cited where historically relevant. The discussion is therefore selective and insight‐driven rather than exhaustive, with the goal of distilling general design principles for durable, high‐efficiency PEC systems.

For clarity and consistency, the nomenclature and abbreviations used throughout this review are summarized in the Abbreviations section, and all performance metrics, electrochemical potentials, and current‐density units have been standardized accordingly.

## 2 Key Fundamental Issues on Photoelectrodes

The long‐term stability for water‐splitting devices is one of the most challenging hurdles for commercialization, which requires operational times of ≥5 years without significant performance degradation, as well as cost‐effective H_2_ production [39, 40, 41]. Most previously reported photoelectrodes have corroded and/or oxidized quickly when they come into contact with the electrolytes used for the PEC water splitting, which usually show high ionic strength. This photoelectrode degradation is due to the very narrow window of stability based on Pourbaix diagrams [42, 43]. Despite this, some photoelectrodes, including Si, III‐V compounds, TiO_2_, BiVO_4,_ and Co_3_O_4_, have demonstrated outstanding long‐term stability after introducing protective strategies [11, 43, 44]. Commonly, the long‐term stability of PEC devices is strongly affected by several factors, including energy level alignment, surface defects, and photocorrosion.

In this review, a PEC photoelectrode is considered as a multi‐component architecture consisting of a photovoltaic (PV) conversion layer that absorbs light and generates charge carriers, and a distinct photocatalytic layer that mediates the interfacial HER/OER reactions; additional junction and protective/passivation layers may also be present. Accordingly, Si and III‐V absorbers are treated as photovoltaic components, while materials such as NiO_x_, CoO_x_, and Pt are discussed as photocatalytic layers that primarily govern surface reaction kinetics rather than light absorption. Additionally, PEC water‐splitting performance is discussed in terms of several standard figures of merit, including STH efficiency, photocurrent density at a given potential (commonly 1.23 V vs. reversible hydrogen electrode (V_RHE_)), onset potential, Faradaic efficiency (FE), applied‐bias photon‐to‐current efficiency (ABPE), and long‐term stability assessed by chronoamperometry. Together, these metrics provide a comprehensive picture of activity, selectivity, and durability, which are all critical for the practical deployment of PEC systems.

**FIGURE 2:**
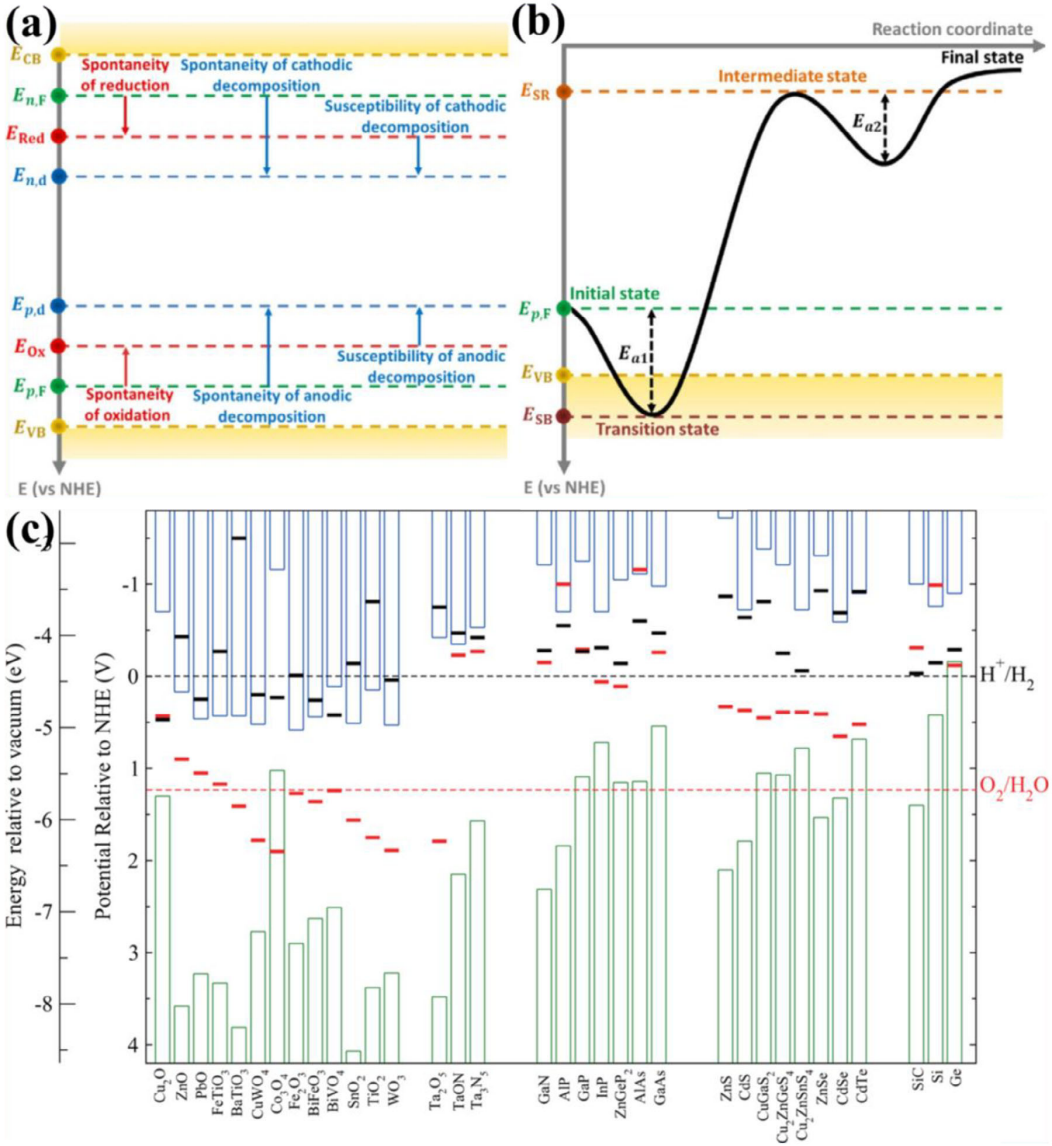
(a) Thermodynamic energy diagram of the photoelectrodes‐liquid electrolyte interface. The directions for each arrow indicate a thermodynamically favorable. E_VB_, E_p,F_, E_Ox_, E_p,d_, E_n,d_, E_Red_, E_n,F_, and E_CB_ are valence band energy, Fermi level of the holes, water oxidation potential, photoelectrode oxidation potential, anodic decomposition potential, cathodic decomposition potential, Fermi level of the electrons, and conduction band energy, respectively. (b) Reaction kinetic diagram of anodic decomposition process at the photoelectrodes‐liquid electrolyte interface by consumption of holes. E_SB_ and E_SR_ are the surface back‐bond energy of the photoelectrode and the surface radical energy, respectively. Reproduced with permission [51]. Copyright 2019, Wiley. (c) Calculated each potential relative to the NHE and vacuum levels for several photoelectrodes in solution at pH = 0, the temperature of 298.15 K, and pressure of 1 bar. Reproduced with permission [45]. Copyright 2012, American Chemical Society.

The origin of instability of photoelectrodes under light irradiation has been well established by previous papers [42, 44, 45, 46, 47, 48, 49, 50]. These studies generally demonstrated that the long‐term stability of photoelectrode is strongly dependent on the alignment of reduction potential (E_Red_), conduction band minimum (CBM) relative to E(H_2_O/H_2,_ water reduction potential) for photocathode and oxidation potential (E_Ox_), valence band maximum (VBM) relative to E(O_2_/H_2_O, water oxidation potential) for each photoelectrode as shown in Figure 2a [43, 51]. The photocathode is stable when the E_red_ is higher than the cathodic decomposition potential (E_n,d_), while the photoanode is stable when the E_Ox_ is lower than the anodic decomposition potential (E_p,d_). The E_n,d,_ and E_p,d_ are determined by the electrolyte pH , which can be thermodynamically calculated or obtained from electrochemical experiments [45, 46]. From literature surveys [45, 46], most photocathodes for HER are over a wide range of pH, while only a few photoanodes for OER, such as BiVO_4_, SnO_2_, WO_3_, Fe_2_O_3_, CuWO_4_, BaTiO_3_, and FeTiO_3,_ are thermodynamically stable against anodic photocorrosion process at a limited pH range. When the photoelectrode is thermodynamically unstable, the photocorrosion (i.e., decomposition) process tends to occur reversibly and compete kinetically with other reactions such as water reduction, oxidation, and interface or surface recombination [51]. The electronic free energy at the photoelectrode/electrolyte interface is less than that of the initial photocorrosion when both reactions are fast [51]. The potential of the photocorrosion reaction at the photoelectrode is normally lower, while it leads to a thermodynamically more favorable reaction than OER or HER [42]. The photocorrosion reaction also occurs when photogenerated charge carriers (i.e., hole or electron) contribute to the oxidation or reduction reaction for the photoelectrode [52]. The photocorrosion process involves complex steps with different activation energies, as shown in Figure 2b. In the initial step, the energy for the photoinduced charge carrier has to reach the surface back‐bond energy (E_sb_) for the photoelectrode, which is the energy to break the bonds between the surface and underlying lattice atoms [53]. The initial step for the photocorrosion reaction requires the activation energy (i.e., E_a1_ = E_sb –_ E_p,F_), which forms the surface radicals with dangling bonds at a high energy level (defined as E_SR_) as shown in Figure 2b. The required activation energy (Ea_2_) for the subsequent second step is mainly dependent on the energy level for dangling bonds. Figure 2c shows an overview of the potentials of several photoelectrodes relative to the normal hydrogen electrode (NHE) and vacuum levels, where the red and black dashed lines indicate the water oxidation and reduction potentials. It demonstrates that not all semiconductor materials are fully satisfied by both thermodynamic and potential conditions for photocorrosion reactions. In this regard, the photogenerated charge carriers for photoelectrodes can easily cause lattice distortions and form localized polarons (so‐called charge‐induced instability), which mainly suppress charge transfer kinetics and finally degrade the stability of photoelectrodes [47, 54].

Another origin of instability in photoelectrodes is the abundant defect sites at the surface of the photoelectrode, which can accelerate the photocorrosion reactions [55]. The defects are created irreversibly during the synthesis of photoelectrodes, particularly based on metal chalcogenide‐based compounds. For the PEC system, atoms at the surface of each photoelectrode are weakly bonded with the underlying lattice and species from the electrolyte. Abundant defect sites at the surface of photoelectrodes affect the adsorption and desorption processes to weaken each bond. Finally, this can accelerate the susceptibility to the photocorrosion process, which is discussed in the following.

The photocorrosion, which adversely affects the PEC water splitting device, must be avoided to ensure longevity [52]. Many researchers have extensively investigated the photocorrosion mechanisms of diverse material‐based photoelectrodes, and these mechanisms have been well‐demonstrated [56, 57, 58]. Table 1 shows photocorrosion reactions of representative photoelectrodes. The critical factors for photocorrosion reactions include (i) positions or alignments of the conduction band (CB) and valence band (VB), [45] (ii) participation in self‐oxidation and/or reduction of photogenerated charge carriers (electrons and/or holes), [44, 47] and (iii) pH of the electrolyte or applied potentials [4, 8], respectively. First, the detailed photocorrosion mechanisms related to band positions in photoelectrodes are discussed already. Second, the photocorrosion reaction occurs at the surface of photoelectrodes via parasitic reactions of self‐oxidation or reduction [44]. Under illumination conditions, the photogenerated charge carriers, such as electrons and/or holes, can be thermodynamically generated from the CB and VB in the photoelectrodes. The electrons at the cathode and holes at the anode are easily transferred to the surface of the photoelectrodes. However, the electrons at the photoanode and holes at the photocathode are the main cause for parasitic reactions (i.e., during reactions like HER and/or OER) [59, 60]. The photogenerated charge carriers could be enriched on the outer surface of photoelectrodes before being consumed by donors or acceptors in the reaction process [61]. The accumulation of excess photoinduced charge carriers on photoelectrode surfaces is the main cause of photo‐induced dissolution. Thirdly, the pH in electrolytes under light irradiation can accelerate the photocorrosion process. At both HER and OER potentials, the long‐term stability of photoelectrodes is significantly dependent on the pH of the electrolyte. This involves an oxidation reaction in acidic conditions or a dissolution reaction in alkaline conditions [8]. In the case of Si‐based photoelectrode, the oxidation reaction between the acidic electrolyte and Si readily forms a thin SiO_2_ layer with high transmittance, and it plays the role of charge injection barrier. In alkaline electrolytes, the formed SiO_2_ on the Si photoelectrode continuously dissolves due to photogenerated charge carriers, eventually leading to a loss of photocurrent density [62, 63]. In particular, this dissolution process will not stop until reaching other materials at the interface or the other side, ultimately causing critical damage to the PEC water‐splitting device under light illumination conditions.

**TABLE 1:** Photocorrosion mechanisms for representative photoelectrodes.

Photoelectrode materials	Overall photocorrosion reactions
Si [56]	Si + 2H_2_O ↔ SiO_2_ + 2H_2_
GaN [64]	GaN + 3H_2_O + 3e^−^ ↔ Ga + NH_3_ + 3OH^−^
Cu_2_O [65, 66, 67, 68]	Cu_2_O + 2OH^−^ + 2H^+^ ↔ 2CuO + H_2_O
	O_2_ + 4e^−^ + 2H^+^ ↔ 2OH^−^
Kesterite [58]	Cu2ZnSnS4 + (x+2z) H+ ↔ Cu2‐xZn1‐ySnS4‐z + xCu2+ + yZn2+ + zS0
Kesterite [58]	Cu_2_ZnSnS_4_ + 2H^+^ ↔ Cu_2_SnS_3_ + Zn^2+^+ zS^0^
	Cu_2_ZnSnS_4_ + 16H_2_O + 36H^+^ ↔ 2Cu^2+^ + Zn^2+^ + Sn^4+^ + 4HSO_4_^−^ + 28H^+^
Sb_2_Se_3_ [57]	Sb_2_Se_3_ + 4OH^−^ ↔ 2Sb(OH)_2_^+^ + 3Se^2−^
Sb_2_Se_3_ [57] CdS [69, 70]	2Sb(OH)_2_^+^ + 2OH^−^ ↔ Sb_2_O_3_ + 3H_2_O
	CdS + 2H+ ↔ Cd^2+^ + S^0^
CdS [69, 70] ZnO [71, 72, 73]	CdS + 2O_2_ ↔ Cd^2+^ + SO_4_^2−^
	2ZnO + 4H^+^ ↔ 2Zn^2+^ + O_2_
TiO_2_ [74, 75]	TiO_2_ + H_3_O^+^ + e^−^ ↔ Ti(OH)_3_
Fe_2_O_3_ [76]	Fe_2_O_3_ + 6H^+^ ↔ 2Fe^3+^ + 3H_2_O
BiVO_4_ [45, 77]	4BiVO_4_ + 12H^+^ + 12Cl^−^ ↔ 4BiCl_3_ + 2V_2_O_5_ + 6H_2_ + 3O_2_

## 3 Current Status of Representative Photoelectrodes for PEC Water Splitting

Generally, PEC systems are considered practically viable when achieving STH efficiencies of at least 10%, while commercialization‐level deployment is expected to require STH efficiencies approaching or exceeding 15% [12, 78]. However, several limitations and unresolved challenges remain, including voltage losses during operation, a substantial gap between demonstrated and required device performance, high hydrogen production costs, and the lack of a mature hydrogen supply infrastructure. For clarity, the photoelectrode materials discussed in this review are classified according to the technological maturity of the semiconductor platform and the current level of PEC development rather than the commercialization status of complete PEC water‐splitting devices. Accordingly, the materials are divided into two categories:

- Established Photoelectrode Materials, including Si, III–V semiconductors, TiO_2_, Fe_2_O_3_, and BiVO_4_. which have been extensively investigated and demonstrate relatively advanced technological readiness
- Emerging/Developing Photoelectrode Materials, including Cu_2_O, kesterite, Sb_2_(S,Se)_3_, which continue to undergo extensive optimization to address challenges related to efficiency, stability, charge transport, and scalability.

In this section, the fundamental properties, PEC performance, and long‐term stability of these representative photoelectrode systems are discussed.

A quick overview of popular and capable constituents utilized in photoelectrodes representing PEC water splitting is presented in Table 2. Table 2 summarizes representative semiconductor materials employed in PEC water splitting, categorized according to the technological maturity of the semiconductor platform. Established photoelectrode materials include Si, III–V, TiO_2_, Fe_2_O_3_, and BiVO_4_. In contrast, Cu_2_O, kesterite, Sb_2_(S,Se)_3_ are classified as emerging or developing PEC photoelectrode materials that require further optimization for practical deployment. The table summarizes established photoelectrodes alongside emerging materials currently being investigated for PEC water‐splitting applications. Established photoelectrode materials provide high performance, but generally require extensive protection against photocorrosion. Established metal‐oxide photoelectrodes offer superior chemical stability but are often limited by poor charge transport and low absorption coefficients. Emerging photoelectrode materials present opportunities for low‐cost and earth‐abundant PEC systems but require substantial improvements in both efficiency and durability. Consequently, a variety of stabilization and performance‐enhancement strategies, discussed in Section 4, have been developed to address these limitations. Additionally, the best PEC performance and long‐term stability for established photoelectrode materials and emerging photoelectrodes are provided in Table 2.

**TABLE 2:** Classification of PEC photoelectrode materials into established photoelectrode materials, and emerging/developing photoelectrode materials, together with their key characteristics, advantages, and limitations.

Materials	Type of photoelectrodes	E_g_ (eV)	Major merits	General demerits
Si	Photoanode (established photoelectrode materials)	1.12	Abundance, low cost, and high theoretical efficiency	Poor stability, photocorrosion, and high interfacial recombination
III‐V (GaAs or others)		1.42–2.26	Optimal E_g_, high efficiency, and carrier mobility	high cost, and severe photocorrosion
TiO_2_		3.0–3.2	Excellent stability, low cost, and non‐toxicity	Wide E_g_ and short carrier diffusion length
Fe_2_O_3_		2.0–2.2	Excellent stability, low cost, non‐toxicity, and reliable E_g_	Short carrier diffusion length and slow oxygen evolution reaction kinetics
Fe_2_TiO_5_		2.2	Excellent stability, reliable E_g_, and non‐toxicity	Extremely high temperature synthesis and frequent secondary phase formation
BiVO_4_		2.4	Superior stability, non‐toxic, and reliable E_g_	High surface recombination rate, poor bulk carrier transport, and requires pH control
Cu_2_O	Photocathode (emerging/developing photoelectrode materials)	2.0	Cost‐effective, reliable E_g_, and non‐toxic elements	Poor photostability
CIGS		1.0–1.7	High light absorption and tunable E_g_	Poor intrinsic stability and photocorrosion
CZTSSe		1.0–1.5	Earth‐abundant, low cost, and non‐toxic	Poor long‐term stability
Sb_2_Se_3_		1.2	Reliable E_g_ and high carrier mobility	Poor long‐term stability

**TABLE 3:** Representative PEC performance and long‐term stability records for established metal‐oxide photoelectrodes and emerging/developing PEC photoelectrode materials.

Materials	Device structure	Performance (mA/cm^2^)	HC‐STH (%)	Onset Potential (≅Photo voltage) (V_RHE_)	Stability (J/J_0_), time, applied bias	Visual reference
Si [85, 86]	Si/NW GaAs/*a*‐TiO_2_/NiO_x_	−9 mA/cm^2^ @0 V_RHE_	—	+0.91	100%, 600 h, 0 V	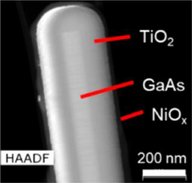
	Glass/SiN_x_/Al_2_O_3_/*p*‐Si/*n*‐Si/Pt	−39 mA/cm^2^ @0 V_RHE_	11.2	+0.54	100%, 370 h, 0 V	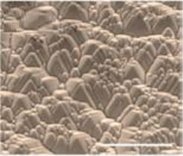
III‐V based compounds [158]	*p*‐GaAs/*n*‐GaAs/n‐AlGaAs/n^+^AgAs/Ti/ Pt	−23.1 mA/cm^2^ @0 V_RHE_	17.0	+1.02	89%, 192 h, 0 V	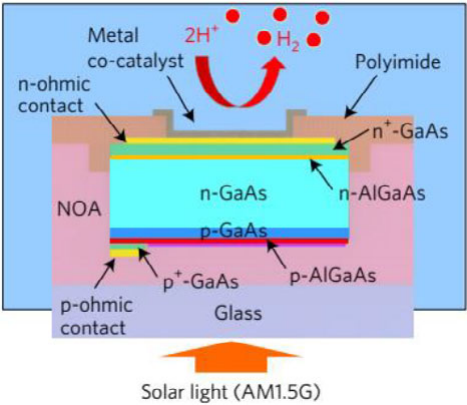
**CIGS** [159]	CIGS/ZnS/CdS/rGO/Pt	−35.5 mA/cm^2^ @0 V_RHE_	9.04	+0.66	85%, 10 h, 0 V	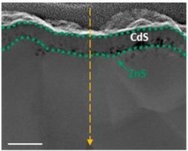
Cu_2_O [107]	Cu_2_O/Ga_2_O_3_/TiO_2_/ RuO_x_	−10 mA/cm^2^ @0 V_RHE_	—	+1.0	93%, 100 h, 0 V	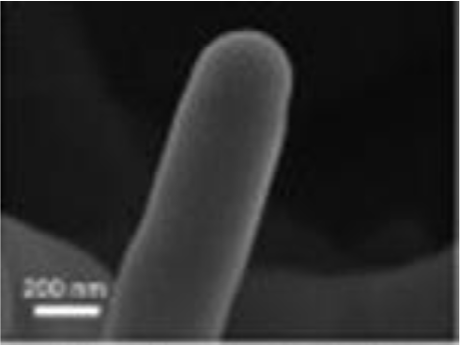
Kesterite [122, 128]	Cu_2_ZnSnS_4_/HfO_2_/CdS/HfO_2_/Pt	−19 mA/cm^2^ @0 V_RHE_	—	+0.72	100%, 24 h, 0V	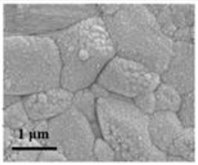
	Cu_2_ZnSn(S,Se)_4_/CdS/TiO_2_/Pt	−40.40 mA/cm^2^ @0 V_RHE_	6.47	+0.55	90%, 1h, 0V	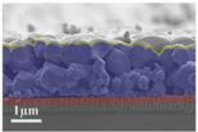
Sb_2_(S,Se)_3_ [137]	Sb_2_Se_3_/AL/GABA/TiO_2_/Pt	−35 mA/cm^2^ @0 V_RHE_	4.7	+0.5	100%, 100 h, 0V	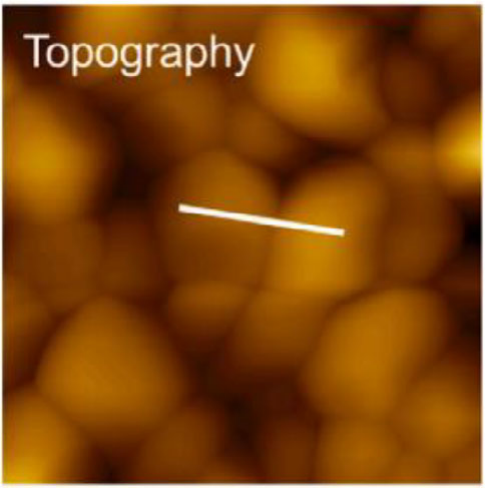
TiO_2_ [144, 145]	S doped TiO_2_ /Cu_2_ZnSnS_4_ NCs	8.84 mA/cm^2^ @1.23 V_RHE_	—	−0.15	80%, 2 h, 1.23 V	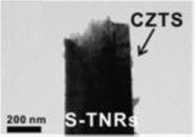
	FTO/TiO_2_/organic	1.68 mA/cm^2^ @1.23 V_RHE_	—	+0.27	100%, 30 days, 1.23 V	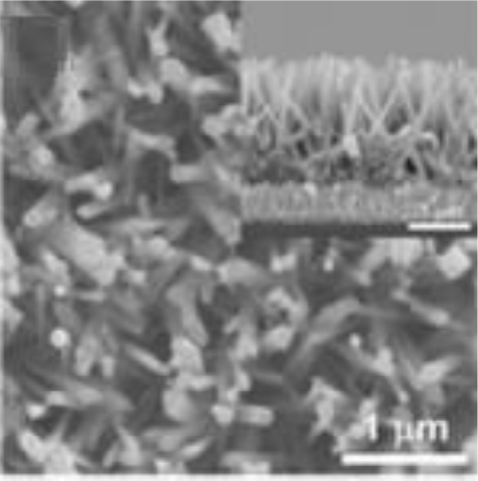
Fe_2_O_3_ [151]	Nb and Sn doped Fe_2_O_3_/FeNbO_4_	2.71 mA/cm^2^ @1.23 V_RHE_	—	+0.69	96%, 20 h, 1.23 V	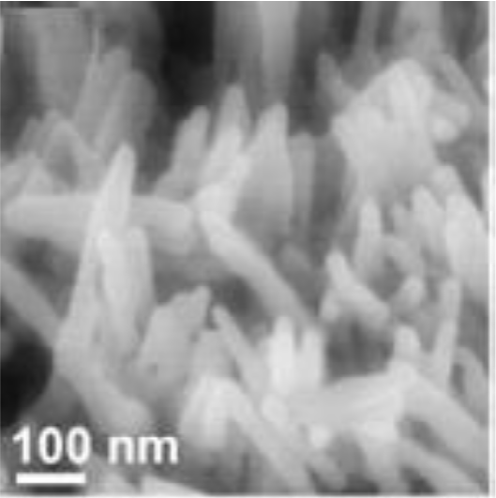
BiVO_4_ [155, 157]	BiVO_4_/ FeOOH/ NiOOH/ Pt	4.7 mA/cm^2^ @1.23 V_RHE_	—	+0.6	100%, 500 h, 1.23 V	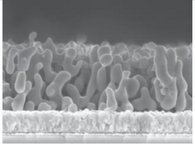
	BiVO_4_/N:NiFeO_x_/Pt	6.4 mA/cm^2^ @1.23 V_RHE_	—	+0.73	100%, 5 h, 1.23 V	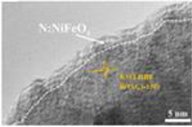

### 3.1 Established Photoelectrode Materials

#### 3.1.1 Si

**FIGURE 3:**
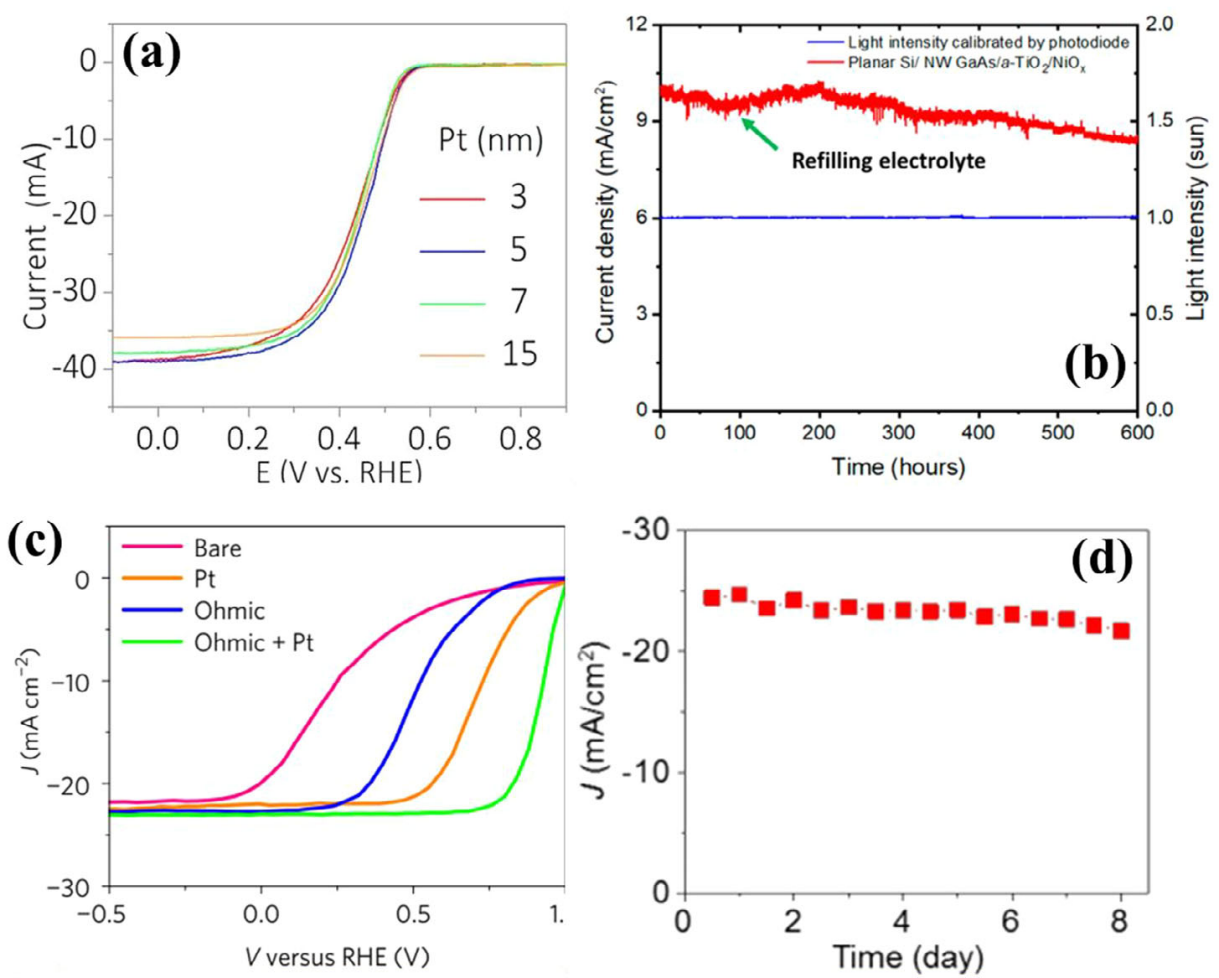
(a) Current density‐potential curves of the Si‐based PEC devices with different thicknesses of Pt. Reproduced with permission [85]. Copyright 2020, Elsevier. (b) Long‐stability results for Si‐based photoelectrodes. Reproduced with permission [86]. Copyright 2021, American Chemical Society. (c) Current density‐potential curves of integrated GaAs photocathodes at various materials configurations, such as GaAs, GaAs/Pt, GaAs with ohmic contact, and GaAs with ohmic contact and Pt, and (d) Photocurrent density vs time of GaAs‐based photoelectrodes. Reproduced with permission [97] Copyright 2017, Nature Publishing Group.

Si has been extensively studied and utilized as established photoelectrode materials for water splitting owing to its suitable E_g_ (∼1.1 eV), low cost, and well‐developed Si‐based PEC industrialization [55, 79]. These are the semiconductor absorbers with established PV manufacturing infrastructure. Although these materials are established photoelectrode materials, PEC systems based on them are still under development and have not yet reached widespread commercialization. Despite these advantages, the poor photovoltage and relatively sluggish kinetics of Si‐based photoelectrodes limit the PEC performance of water splitting devices. Several strategies, such as adjusting band alignment, creating homo/heterojunctions, extending active area, enhancing light absorption, and introducing a photoactive layer and new catalysts, have been extensively reported [80, 81, 82]. These strategies have led to significant improvements in PEC performance of Si‐based photoelectrodes in recent years. Among them, the following paragraphs briefly summarize the outstanding PCE performance and long‐term stability of Si‐based photoelectrodes. Lei and co‐workers successfully developed the three‐dimensional (3D) p‐Si nanopillar/NiCoSe_x_/Pt structured photocathode with a significantly improved photocurrent density of −37.5 mA/cm^2^ at 0 V_(reversible hydrogen electrode (RHE))_ and excellent onset potential of +0.31 V_RHE_ [83]. This photocathode demonstrated relatively poor long‐term stability with an initial photocurrent density of 91% after 2 h. On the other hand, the significantly improved long‐term stability of inverted pyramid textured Si/Co_2_P structured photocathode over 150 h without notable degradation in photocurrent density (−35.2 mA/cm^2^ at 0 V_RHE_) has been reported by Liu and co‐workers [84]. Recently, He and co‐workers reported the simultaneously improved PEC performance (−39.1 mA/cm^2^ at 0 V_RHE_) and long‐term stability (370 h without degradation in the acidic electrolyte) of a bifacial designed Si‐based photocathode shown in Figure 3a [85]. Interestingly, this bifacial Si‐based photocathode showed excellent photocurrent density (−61.20 mA/cm^2^ at 0 V_RHE_) and yielded 56.88% of excess H_2_ generation. Furthermore, Lewis and co‐workers demonstrated excellent long‐term stability over 600 h without substantial photocurrent density decay of a Si/nano‐wired GaAs/amorphous (*a*)‐TiO_2_/NiO_x_ structured photoanode, as shown in Figure 3b [86]. Authors suggested that *a*‐TiO_2_ layer on nano‐wired GaAs can successfully passivate defects and suppress the corrosion reaction during the water‐splitting process.

#### 3.1.2 III‐V Based Compounds

III‐V compounds are representative of well‐developed and established photoelectrode materials for photocatalysis and PEC applications in solar fuel generation owing to their suitable E_g_, favorable band alignment, and excellent photophysical properties for the collection and transport of photogenerated carriers [87, 88, 89]. Like other photoelectrodes, III‐V‐based compounds also suffer corrosion reactions in highly acidic or alkaline electrolytes under the relevant electrochemical conditions of water splitting, resulting in the rapid degradation of the properties of photoelectrodes and their PEC performance [90, 91]. Several strategies for III‐V compound‐based photoelectrodes have been studied, and the improved PEC performances and long‐term stability have been achieved [16, 92, 93, 94, 95, 96]. Among them, Yoon and co‐workers reported excellent PEC performance (photocurrent density of −23.1 mA/cm^2^ at 0 V_RHE_ and an onset potential of + 1.0 V_RHE_) and long‐term stability (192 h without degradation in acidic electrolyte at pH 0.55) of the printed assemblies of epitaxially grown GaAs‐based photoelectrodes as shown in Figure 3c,d. Particularly, this photoelectrode demonstrated over 10% STH efficiency under unassisted water‐splitting conditions.

### 3.2 Emerging and Developing PEC Photoelectrode Materials

#### 3.2.1 Cu_2_O

**FIGURE 4:**
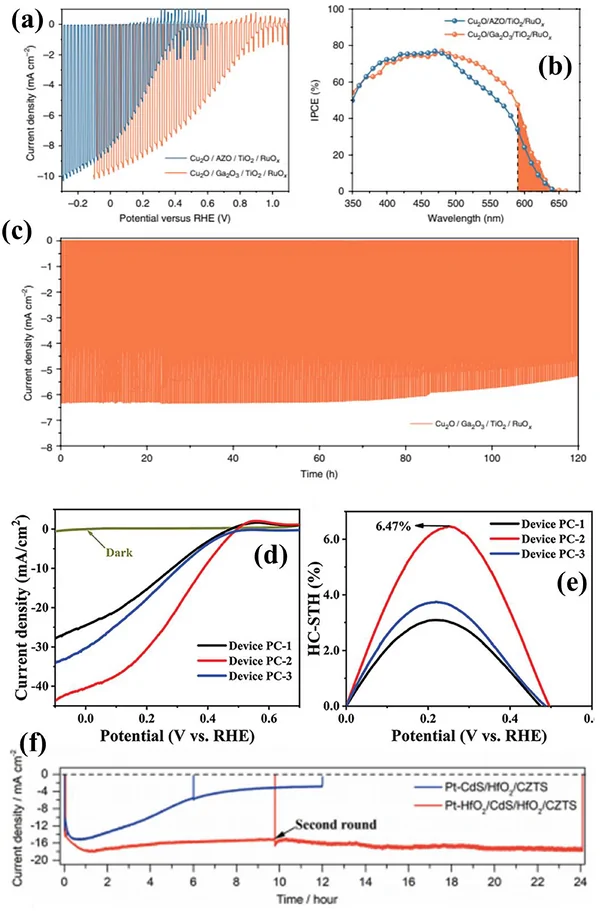
(a) Photocurrent density vs potentials, (b) corresponding wavelength‐dependent IPCE spectra, and (c) long‐term stability test for Cu_2_O/Ga_2_O_3_/TiO_2_/RuO_x_ photocathode. Reproduced with permission [107]. Copyright 2023, Nature Publishing Group. (d) photocurrent density vs potentials and (e) calculated HC‐STH efficiencies for CZTSSe‐based photocathodes. Reproduced with permission [129]. Copyright 2023, Wiley. (f) Long‐term stability test for CZTS/HfO_2_/CdS/HfO_2_/Pt photocathode. Reproduced with permission [128]. Copyright 2021, Royal Society of Chemistry (RSC).

Cu_2_O is well‐known as a promising photocathode material for HER owing to its outstanding optoelectronic properties (i.e., a direct type E_g_ of 2.0 eV, long charge carrier diffusion lengths (∼ several micrometers), and high hole mobility of 100 cm^2^/Vs) and favorable band alignment [3, 55]. Unfortunately, Cu_2_O‐based photoelectrodes show inferior PEC performance and STH efficiency compared to their theoretical value (∼ −14.7 mA/cm^2^ and ∼18%) because of the poor charge separation at the photocathode/electrolyte interface, interface mismatching of the photocathode with electron/hole selective layers (i.e., hole transport layer (HTL), electron transport layer (ETL)), and high recombination [98]. To overcome these issues, several strategies have been explored, including introducing a protective layer, HTL, ETL, and engineering properties of Cu_2_O by doping with Fe or B, designing nanostructures (i.e., 3D), and cocatalysts [55, 99, 100, 101, 102, 103, 104, 105, 106]. Through interesting and impressive studies, Grätzel and co‐workers reported significantly improved PEC performance (photocurrent density of −10 mA/cm^2^ at 0 V_RHE_ and an onset potential of + 1.0 V_RHE_) and long‐term stability (93% of initial photocurrent density after operation over 100 h) of Cu_2_O‐based photocathode by introducing Ga_2_O_3_ interfacial layer between Cu_2_O and TiO_2_ as shown in Figure 4a–c [107]. These photocathodes showed an improved incident photon‐to‐current conversion efficiency (IPCE) spectrum over 500 nm wavelength, leading to a total 6% improvement in photocurrent density compared to that without a Ga_2_O_3_ interfacial layer. Additionally, authors suggested the significantly improved long‐term stability was attributed to the introduction of a 100 nm thick TiO_2_ overlayer on the Ga_2_O_3_/Cu_2_O structured photocathodes. Unfortunately, further improvement of PEC performance and long‐term stability of Cu_2_O‐based photocathode has not been reported yet.

#### 3.2.2 Cu_2_ZnSn(S,Se)_4_ (CZTSSe)

Kesterite compounds have emerged as one of the promising photocathode materials for PEC water splitting devices owing to their outstanding optoelectronic properties, such as a tunable E_g_ from 1.0 to 1.5 eV, high absorption coefficient over 10^4^ cm^−1^, and favorable band alignment, as well as low cost and non‐toxicity [108, 109, 110, 111, 112, 113, 114, 115, 116, 117, 118, 119, 120, 121]. The theoretical photocurrent density and STH efficiency of kesterite‐based photocathode are predicted to be ∼ −42.85 mA/cm^2^ and 30%, [122] however, reported studies presented inferior PEC performances owing to their abundant anti‐site defects, related defect density, and vulnerability to photocorrosion under strong acidic electrolytes. In this regard, the previous reports have focused on suppressing anti‐site defects using adjusting synthesis processes or partially substituting the cation site and introducing a protective layer on the kesterite photocathode [123, 124, 125, 126, 127]. Recently, Chen and co‐workers reported the impressively improved photocurrent density of −40.4 mA/cm^2^ at 0 V_RHE_ and high half‐cell (HC)‐STH efficiency of 6.47% in CZTSSe/CdS/TiO_2_/Pt structured photocathode by preparing high‐quality CZTSSe thin films with compact and uniform grains using a stepwise process as shown in Figure 4d,e [122]. However, the long‐term stability of this photocathode (90% of the initial photocurrent density after 1 h) was inferior to that of other photocathodes. Another impressive study, which reported significantly improved long‐term stability (no notable degradation after 24 h) of a kesterite‐based photocathode, was achieved by introducing the HfO_2_/CdS/HfO_2_ sandwich‐structured buffer layer, as shown in Figure 4f [128]. This photocathode exhibited an excellent STH efficiency of 7.27% and a large onset potential value of + 0.72 V_RHE_.

#### 3.2.3 Sb_2_(S,Se)_3_

**FIGURE 5:**
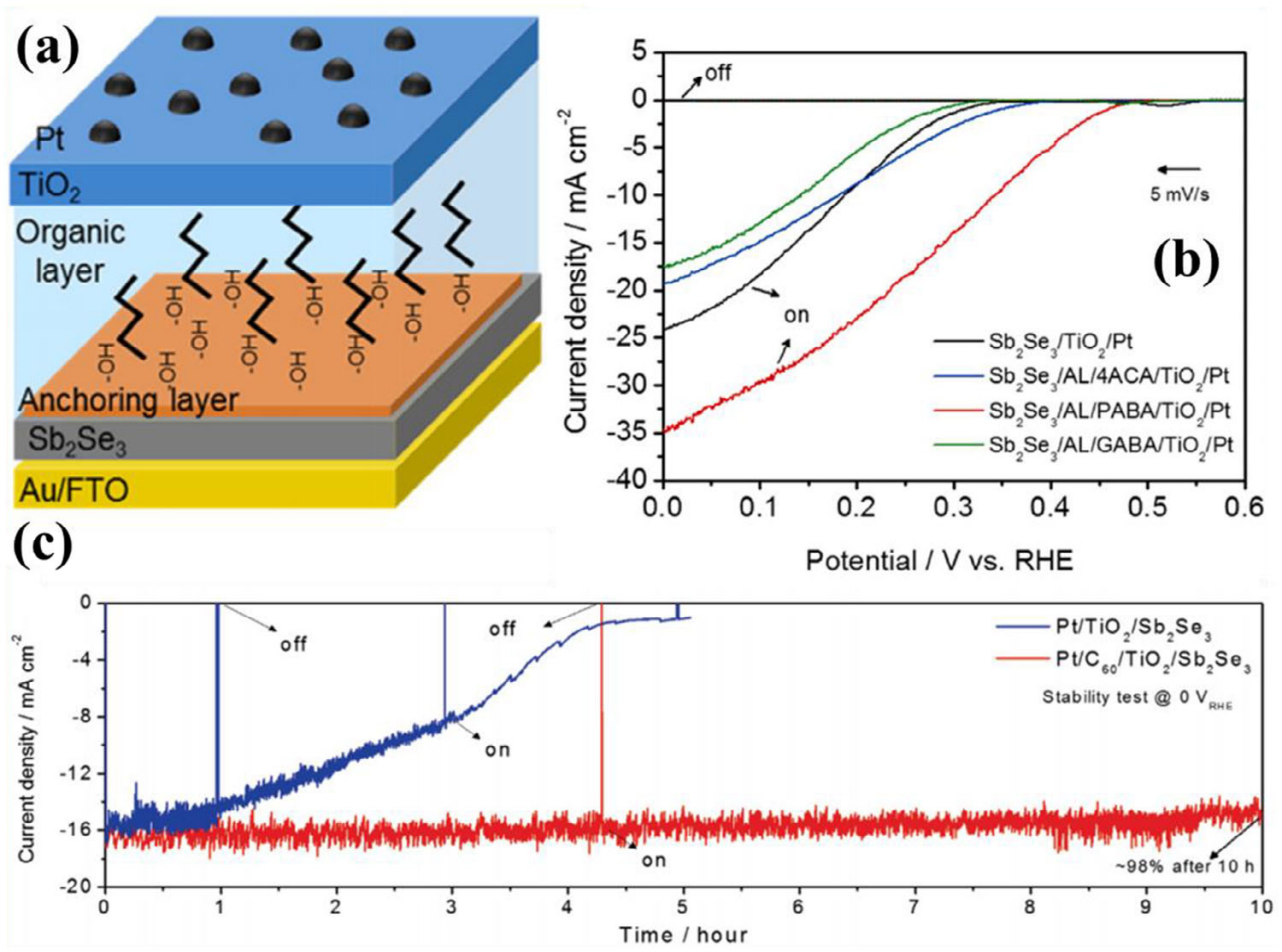
(a) Diagrammatic representation of the photocathodes in the organic layer/TiO_2_/Pt/FTO/Au/Sb_2_Se_3_/AL configuration, (b) photocurrent density vs potential of the Sb_2_Se_3_‐based photocathodes. Reproduced with permission [137]. Copyright 2021, Elsevier (c) long‐term stability tests of the Sb_2_Se_3_‐based photocathodes. Reproduced with permission [136]. Copyright 2019, Wiley.

Sb_2_Se_3_ is well‐known as an emerging photocathode material for water splitting owing to its narrow E_g_ of 1.0 – 1.3 eV, high absorption coefficient over 10^5^ cm^−1^, and excellent theoretical STH efficiency over 30% as well as simplicity of its constituent elements compared to other materials [130, 131, 132, 133]. In particular, the one‐dimensional Sb‐Se nanoribbons‐based unique crystal structure of Sb_2_Se_3_ contributed to the outstanding charge carrier mobility and transport properties, finally leading to the improved PEC performance [3]. Extensive research for Sb_2_Se_3_‐based photocathodes has been led by Prof. Moon`s group, focusing on various strategies [134, 135, 136]. Among these, Moon and co‐workers reported excellent PEC performance (−35 mA/cm^2^ at 0 V_RHE_) and long‐term stability (98% initial photocurrent density after 10 h) by introducing an organic molecule between Sb_2_Se_3_ and TiO_2_ and a combination of TiO_2_/C_60_ protective layer as shown in Figure 5 [136]. However, the long‐term stability of the Sb_2_Se_3_‐based photocathode was significantly poor compared to that of other photocathodes (See Table 3).

### 3.3 Established Metal‐Oxide Photoelectrodes

#### 3.3.1 TiO_2_

**FIGURE 6:**
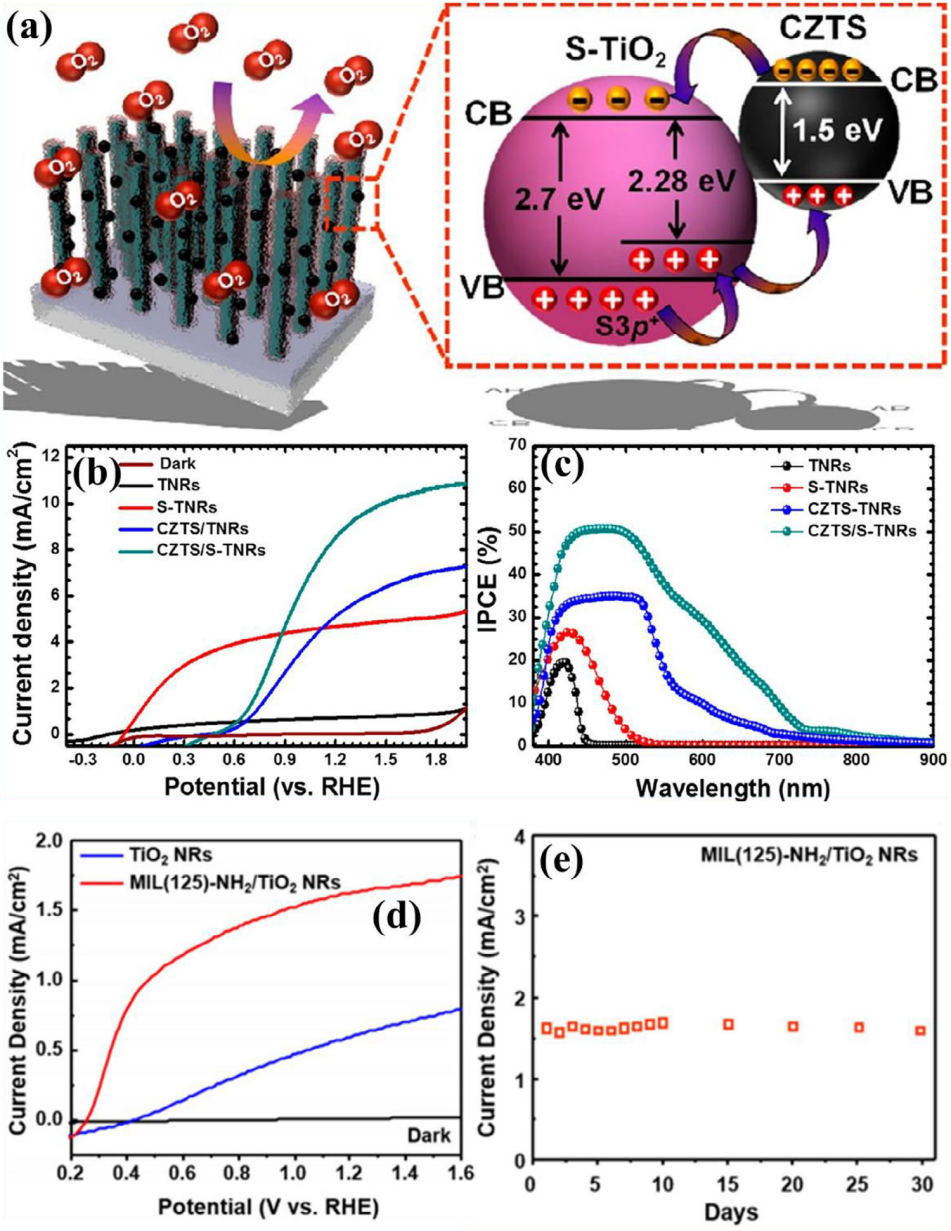
(a) Scheme illustration for charge transfer mechanism, (b) photocurrent density vs potentials, and (c) IPCE spectra for TiO_2_‐based photoelectrodes. Reproduced with permission [144]. Copyright 2017, American Chemical Society. (d) photocurrent density vs potentials and (e) long‐term stability test for TiO_2_‐based photoelectrodes. Reproduced with permission [145]. Copyright 2019, Elsevier.

TiO_2_ was first reported as a material for water splitting by Honda and Fujishima in 1972 under UV‐Vis illumination [138, 139]. TiO_2_ possesses merits such as non‐toxicity and chemical stability, high resistance to photocorrosion, and is therefore one of the most widely studied photoanode [140, 141]. Despite these outstanding properties and extensive investigations, the PEC performance of TiO_2_‐based photoanodes is limited owing to their rapid recombination rate and short hole diffusion lengths [3]. Several strategies, including microstructure modification, surface, E_g_, and junction‐engineering, have been developed to improve the performance of TiO_2_‐based photoanodes [142, 143]. Among extensive studies, Kim and co‐workers reported a significantly improved photocurrent density (8.8 mA/cm^2^ at 1.23 V_RHE_) compared to that of bare TiO_2_ (0.73 mA/cm^2^ at 1.23 V_RHE_) by introducing absorbing nanoparticles on a 3D nanostructure and E_g_ engineering in TiO_2,_ shown in Figure 6a–c [144]. The authors proposed the charge‐transport mechanism using density functional theory calculations and various characterizations. Unfortunately, this photoanode exhibited poor long‐term stability of 80% initial photocurrent density after 2 h. On the other hand, Lee and co‐workers demonstrated a significantly improved photocurrent density (1.63 mA/cm^2^ at 1.23 V_RHE_) and excellent long‐term stability (no notable degradation after 30 days) of a TiO_2_‐based photoanode by introducing a Ti metal‐organic framework overlayer on the surface of TiO_2_ nanorods and without any co‐catalyst, as shown in Figure 6d,e [145]. However, TiO_2_‐based photoanodes have limited PEC performance owing to their wide E_g_. Therefore, E_g_ engineering, the introduction of narrow E_g_ materials on TiO_2_, and interface engineering‐related studies have focused on improving PEC, although they show inferior long‐term stability compared to bare TiO_2_.

#### 3.3.2 Fe_2_O_3_

**FIGURE 7:**
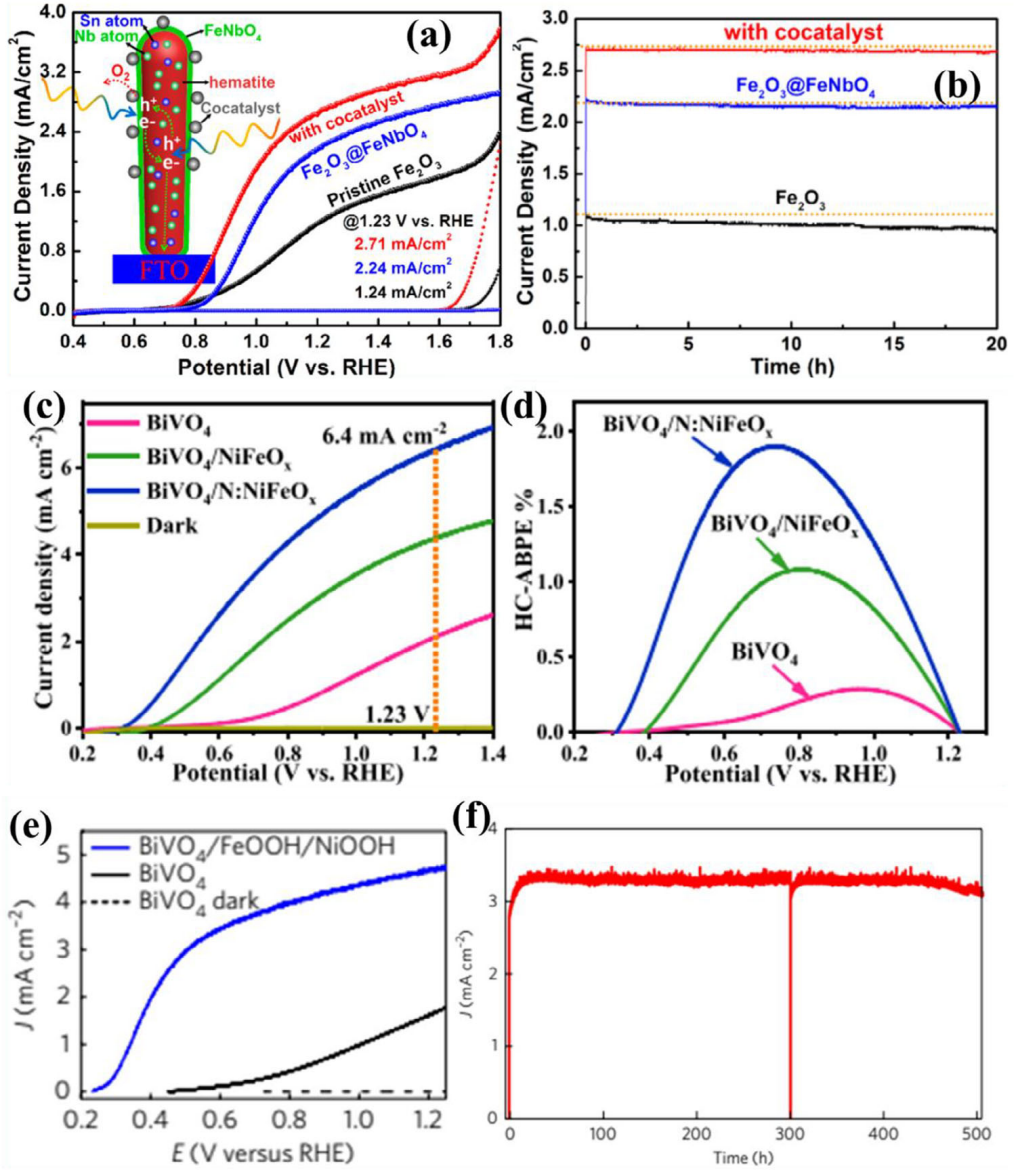
(a) Photocurrent density vs potential and (b) long‐term stability tests for Fe_2_O_3_‐based photoelectrodes. The inserted figure in (a) is an illustration of the charge transfer mechanism. Reproduced with permission [151]. Copyright 2019, American Chemical Society. (c) Photocurrent density vs potentials and (d) HC‐ABPE spectra for BiVO_4_‐based photoelectrodes. Reproduced with permission [155]. Copyright 2021 Nature Publishing Group. (e) Photocurrent density vs potential and (f) long‐term stability for BiVO_4_/FeOOH/NiOOH photoelectrode. Reproduced with permission [157]. Copyright 2018, Nature Publishing Group.

Fe_2_O_3_ is a promising candidate for PEC water oxidation owing to its suitable E_g_ from 1.9 to 2.2 eV, non‐toxicity, and chemical stability, as well as excellent theoretical photocurrent density (12.5–16.6 mA/cm^2^ at 1.23 V_RHE_) and STH efficiency (15.4–16.8%) at AM 1.5G illumination conditions [146, 147, 148]. Unfortunately, the PEC performance of Fe_2_O_3_‐based photoanodes is still limited by high recombination rates in the bulk and at the interface. This limitation results from an absorption coefficient, short excited‐state lifetime (10^−6^ s), slow OER kinetics, short hole diffusion length, and poor electrical conductivity [149, 150]. Various strategies, such as metal and non‐metal doping, surface engineering, fabricating nanostructures, improving charge transfer, and interface engineering, have been investigated to enhance the PEC performance and long‐term stability of Fe_2_O_3_‐based photoanodes [3]. Impressive studies for Fe_2_O_3_‐based photoanode through introducing FeNbO_4_ overlayers have been reported by Lee and co‐workers, shown in Figure 7a,b [151]. The Fe_2_O_3_/FeNbO_4_ photoanode exhibited a significantly improved photocurrent density (2.71 mA/cm^2^ at 1.23 V_RHE_) under simulated sunlight (100 mW/cm^2^) with ∼100% FE of H_2_ production, compared to that of bare Fe_2_O_3_ (0.8 mA/cm^2^ at 1.23 V_RHE_). Furthermore, it showed no notable degradation after 20 h.

#### 3.3.3 BiVO_4_

Satama and co‐workers first discovered the photocatalytic water oxidation property for BiVO_4_ and it has emerged as the most promising photoanode owing to its unique monoclinic scheelite‐based phase, direct‐type E_g_ (∼2.4 eV), theoretical photocurrent density (∼7.5 mA/cm^2^) and STH efficiency (∼9.2%) under AM 1.5G illumination condition, high onset potential (∼1 V_RHE_ with co‐catalyst) as well as slow photocorrosion rate [152, 153, 154]. Through extensive efforts and strategies, the high photocurrent density (∼6.4 mA/cm^2^ at 1.23 V_RHE_) and the ABPE for BiVO_4_‐based photoanode using N:NiFeO_x_ co‐catalysts reported by Bi and co‐workers as shown in Figure 7c,d [155]. Authors revealed that the electron‐enriched Ni sites contributed electrons to the V site in BiVO_4_, which suppresses V^5+^ dissolution during water oxidation reactions. Furthermore, the enhanced hole‐attracting ability of the Fe site significantly contributed to the OER activity. These synergistic effects resulted in significant charge transfer characteristics and PEC performance. However, this study showed good long‐term stability with short periods (no notable degradation after 5 h). The previous studies suggested that the rapid dissolution of V^5+^ ions from the BiVO_4_ surface is the main cause of poor long‐term stability [156, 157]. In this regard, Choi and co‐workers have demonstrated significantly improved long‐term stability with no notable degradation after 500 h for a BiVO_4_‐based photoanode under V‐ion‐containing electrolyte, as shown in Figure 7e,f [157]. Although this strategy effectively prevented the photocorrosion of the BiVO_4_ photoanode, the use of complex electrolytes may limit early commercialization.

## 4 Strategies for Enhancing Photoelectrode Long‐Term Stability

Enhancing the long‐term stability of photoelectrodes in electrolytes is a critical challenge in the advancement of PEC water‐splitting technology. The demand for stable photoelectrodes in PEC systems has driven significant innovation and research progress in the field [6]. The long‐term stability of photoelectrodes is essential for sustained and efficient solar energy conversion. The previously reported investigations have clearly highlighted the essential factors for long‐term stability in PEC devices. In this section, we will review several protection strategies for photoelectrodes, including physical separations (i.e., a protective layer, Section 4.1), interface engineering for catalyst/protective layers (Section 4.2), and strategies to mitigate unfavorable chemical dissolution during PEC operation (Section 4.3). Here, stabilization strategies are classified according to their primary functional role at the photoelectrode (protective passivation, catalytic enhancement, or interface/junction engineering), and processing methods are discussed separately to avoid overlap among categories. To improve clarity, the strategies discussed in this section are categorized according to their primary functional contribution to device stability;

- Protective passivation strategies, which suppress photocorrosion and chemical degradation.
- Interface and junction engineering approaches, which improve charge separation and suppress recombination.
- Electrolyte engineering strategies, which stabilize semiconductor surfaces and mitigate dissolution processes.
- Deposition and fabrication approaches, which influence film quality, defect density, conformality, and interfacial stability.

### 4.1 Surface Passivation

As mentioned in Section 2, the primary cause of instability of the PEC water splitting device is the photocorrosion of the photoelectrode during the PEC reaction process. This photocorrosion process can easily occur when photogenerated charges drive the self‐oxidation or reduction of the photoelectrode rather than the OER or HER [42, 47]. Among several strategies for long‐term stability in PEC devices [160], the physical separation of photoelectrodes (generally, semiconductor materials) is the most widespread and effective, involving the introduction of a protective layer on the photoelectrode. Experimentally, the protective layer plays a crucial role in improving thermodynamically and chemical long‐term stability and providing a surface passivation effect for defects in photoelectrodes, ultimately facilitating charge transfer. Excellent reviews on protective layers for PEC devices have been published [8, 42, 43, 161]. In this section, recently published protective layers are categorized into four groups: metal‐based, metal‐oxide‐based, amorphous, and other protective materials.

#### 4.1.1 Metal‐Based Layer

**FIGURE 8:**
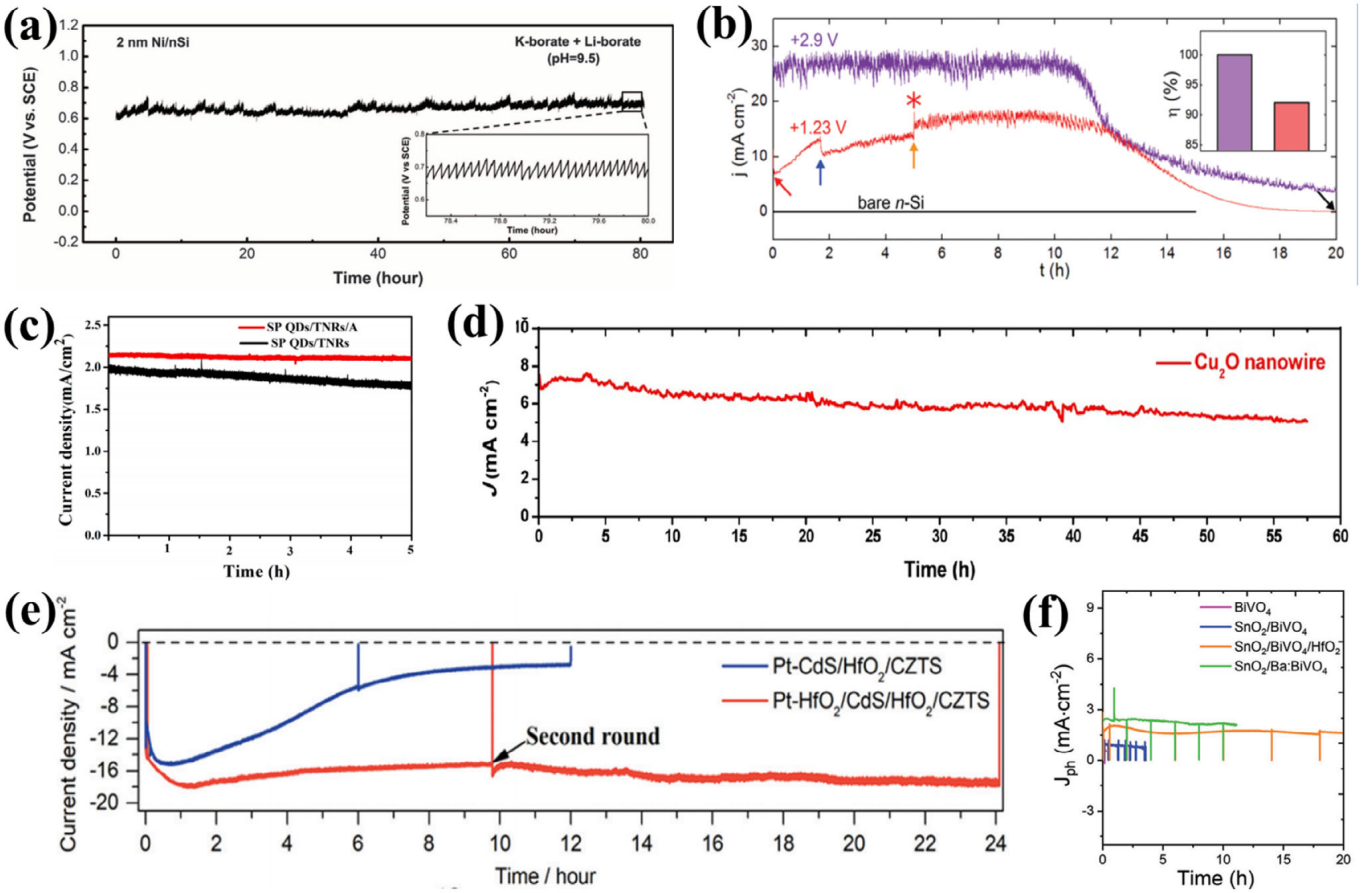
(a) Potential versus time data for Ni/Si‐based photoanode in 0.65 M K‐borate and 0.35 M Li‐borate (pH = 9.5) over 80 h under constant current density at 10 mA/cm^2^ and illumination. Reproduced with permission [94]. Copyright 2013 American Association for the Advancement of Science (AAS). (b) Photocurrent density versus time data for Ni/Si‐based photoanode in 1 M NaOH electrolyte at 2.9 V (purple) and 1.23 V (red). The inserted figure is the OER FE for each condition. Reproduced with permission [167]. Copyright 2018, Royal Society of Chemistry (RSC). (c) Photocurrent density versus time data for SiP QDs/TiO_2_ NRs without and with Al_2_O_3_ layer measured under simulated solar light (100 mW/cm^2^, AM 1.5G) illumination. Reproduced with permission [170]. Copyright 2022, Elsevier. (d) Measured photocurrent density for hydrogen evolution of Cu_2_O NW PEC device under constant bias at 0 V_RHE_, pH 5 electrolyte, and simulated AM 1.5 G illumination. Reproduced with permission [180]. Copyright 2016, American Chemical Society. (e) Long‐term stability results for Pt/CdS/HfO_2_/CZTS‐ and Pt/HfO_2_/CdS/HfO_2_/CZTS‐structured photocathode in 0.2 M Na_2_HPO_4_/NaH_2_PO_4_ solution (pH 6.5) under solar simulated AM 1.5 G light irradiation. Reproduced with permission [128]. Copyright 2021, Royal Chemical Society. (f) Long‐term stability results for BiVO_4_‐based photoanodes at 1.23 V_RHE_ in 1 M KB_i_ electrolyte under AM 1.5G and 1 sun illumination. Reproduced with permission [185]. Copyright 2024, Wiley‐VCH.

Metal‐based protective layers show improved charge transfer due to excellent electrical properties, even though their high visible light reflection and/or absorption can generate parasitic current density loss [160, 162]. Specifically, metal‐based protective layers can be readily formed into a Schottky junction at the metal/semiconductor (i.e., photoelectrodes) interface. This suppresses the photocorrosion process, facilitates the photogenerated charge separation through the band bending phenomenon, and improves photovoltage in the photoanode [94, 163]. Inspired by these merits, noble and non‐noble metals have been introduced as a protective layer on photoelectrodes like Si or III‐V based compounds to enhance long‐term stability [164, 165]. Experimentally, a metallic protective layer can be easily formed as metal‐oxide, metal‐(OH)_x_, or metal‐OOH phase at the metal‐electrolyte interface under basic electrolyte conditions. This offers better protection against the photocorrosion process in PEC devices [20]. Kenney and co‐workers successfully investigated an ultrathin Ni metallic protective layer (∼2 nm) on Si‐based photoelectrodes in an alkaline electrolyte [94]. Here, the Ni metallic protective layer is thin enough to allow light to pass through and reach the photoelectrode. During the PEC reaction, thin NiO_x_ layers were formed on the surface of the Ni protective layer, and they played the roles of an additional protective layer and an effective OER catalyst. As a result, NiO_x_/Ni/Si‐based photoelectrode exhibited excellent PEC performances (∼40 mA/cm^2^ at 1.23 V_RHE_), long‐term stability over 80 h at pH 9.5, as well as a large photovoltage of 1.07 V, as shown in Figure 8a. Further detailed information and discussion regarding the in situ protective layer and catalyst formation will be presented in Section 4.3.

Similarly, Shi and co‐workers successfully introduced NiFe alloys with a 6 nm thickness as a uniform protective layer on SiO_x_/Si structured photoanode in alkaline electrolyte [166]. As a result, this photoelectrode exhibited a low onset potential of 0.89 V_RHE_ and excellent photocurrent density of −30.7 mA/cm^2^ at 0 V_RHE_, as well as STH efficiency of 3.3%. Particularly, the excellent long‐term stability over 100 h was achieved at 1.8 V_RHE_ in 1.0 M K‐borate electrolyte under AM 1.5G and 1 sun illumination. Furthermore, Loget and co‐workers demonstrated the Ni metal nanoparticle (NP) partially (less than 20%) coated Si‐based photoelectrode by a simple low‐cost solution approach in alkaline solution [167]. The PEC performances were significantly enhanced by high activity around the Ni NPs. The Ni/SiO_x_/Si structured photoanode exhibited excellent long‐term stability over 10 h at 1.23 and 2.9 V_RHE_ without degradation, as shown in Figure 8b. The authors also investigated the surface properties for long‐term stability under operation and unbiased conditions to propose a protection mechanism. Previous reports suggested that metallic protective layers effectively improved long‐term stability; however, some challenges remain, including large parasitic photocurrent density loss from unexpected light absorption in the metallic layer, insufficient protection ability against the photocorrosion process, and an unclear protection mechanism.

#### 4.1.2 Metal Oxide‐Based Layer

Metal‐based protective layers are an effective strategy for improving long‐term stability in PEC devices; however, they often come with several drawbacks that can adversely affect the PEC system's long‐term stability and effectiveness. In particular, absorption and/or reflection of incident light by the photoelectrode can dramatically reduce photocurrent density [160, 162]. In this regard, metal oxide‐based single protective layers with high optical transparency in the visible region have been widely investigated to improve photocurrent density as well as long‐term stability [168, 169]. Recently, Zhang and co‐workers [170] reported the use of atomic layer deposition (ALD)‐Al_2_O_3_ as a passivation layer on silicon phosphide (SiP)‐based photoelectrode. This study reports that a higher photocurrent density of 2.14 mA/cm^2^ at 1.23 V_RHE_ and a higher IPCE spectrum of SiP quantum dots (QDs)/TiO_2_ NRs structured photoelectrode after introducing Al_2_O_3_ as a protective layer. A very thin ALD‐Al_2_O_3_ single protective layer can contribute significantly to improving the long‐term stability of the photoelectrode without degradation in photocurrent density after operation for 5 h, compared to that without Al_2_O_3_, as shown in Figure 8c. The study also suggested that a thin Al_2_O_3_ protective layer could offer good adhesion between SiP QDs and TiO_2_ NRs, preventing photocorrosion, while also promoting the charge carrier separation and transport. Similar results regarding the introduction of Al_2_O_3_ as a single protective layer on photoelectrodes, leading to significantly improved long‐term stability of PEC devices, have been extensively reported [171, 172, 173, 174, 175].

Because of its great stability and cost‐effectiveness, TiO_2_ has been extensively researched for use as a promising protective material on photoelectrodes in PEC devices [176]. Its wide E_g_ makes it transparent to visible light, indicating its suitability as a protective material in PEC devices. A single protective layer of TiO_2_ has been widely used to improve the long‐term stability of Si‐based photoelectrodes [177, 178]. The TiO_2_ layer protects the Si photoelectrode surface from degradation and ultimately reduces surface recombination [179] Based on this, Grätzel and co‐workers [180] successfully introduced an ALD‐TiO_2_‐based blocking layer to prevent direct contact of the exposed Cu metal surface on Cu_2_O NWs‐based photocathode with *n*‐type overlayer, leading to the formation of high quality *p‐n* junction. A bare Cu_2_O NWs‐based photocathode suffered photocorrosion in an acid electrolyte, leading to the formation of CuO on the Cu_2_O photocathode. Unfortunately, authors did not investigate the detailed role of the formed CuO layer during PEC operation. Surprisingly, the Cu_2_O NWs‐based photocathode with *a*‐TiO_2_ protective layer showed excellent long‐term stability over 55 h with a constant FE of ∼100% in acidic medium (pH 5), as shown in Figure 8d. After exchanging the electrolyte, the photocurrent density of Cu_2_O NWs‐based photocathode showed a similar photocurrent density to that observed at the initial stage. Similar approaches using ALD or chemical‐based *a*‐TiO_2_ as a protective layer on the photoelectrode have been reported, demonstrating significantly improved long‐term stability of the PEC devices [8, 14, 162].

On the other hand, Ga_2_O_3_ is known as an *n*‐type semiconductor with a wide E_g_ of 4.9 eV, excellent chemical resistance, desirable electrical properties, and thermal stability [107, 180]. Kang and co‐workers [181] successfully demonstrated a *β*‐Ga_2_O_3_ layer as a protective layer on a GaN photoanode through photo‐assisted electrodeposition. The *β*‐Ga_2_O_3_/GaN photoanode showed an enhanced photocurrent density of 1.2 mA/cm^2^ at 1.23 V_RHE_ without degradation over 5 h. After introducing Ni‐P_i_ as co‐catalysts on *β*‐Ga_2_O_3_/GaN photoanode, the long‐term stability was significantly improved to over 25 h without degradation of photocurrent density in 0.5 M NaOH electrolyte. Additionally, authors also proposed suitable protection and PEC working mechanisms for GaN‐based photoanodes. Furthermore, HfO_2_ is a promising material for photocorrosion‐resistant coatings owing to its excellent thermal stability, wide E_g_, and strong chemical resistance [182]. In this regard, HfO_2_ has been widely investigated for passivation materials for both photocathodes and photoanodes in the PEC devices [183, 184]. Jiang and co‐workers successfully demonstrated significantly improved PEC performance (‐20 mA/cm^2^ at 0 V_RHE_), high ABPE of 7.27% and long‐term stability over 24 h for CZTS‐based photocathodes by introducing HfO_2_/CdS/HfO_2_ protective layer in 0.2 M Na_2_HPO_4_/NaH_2_PO_4_ electrolyte (pH 6.5) under solar simulated AM 1.5G light irradiation condition as shown in Figure 8e. Notably, the CZTS photocathode in a tandem device with the BiVO_4_ photoanode achieved excellent long‐term stability over 60 h and an STH efficiency over 3%. Authors revealed that (i) the HfO_2_ layer between the CZTS and CdS interface significantly reduced the carrier recombination rate in the photocathode and (ii) HfO_2_ between the CdS and Pt interface effectively facilitated the charge transfer. On the other hand, Kang and co‐workers reported significantly improved photocurrent density (∼6.5 mA/cm^2^ at 1.23 V_RHE_) and long‐term stability (20 h) in 1 M KB_i_ electrolyte (pH 9.5) under AM 1.5G and 1 sun illumination, as shown in Figure 8f [185]. Authors suggested that a semi‐crystalline passivated HfO_2_ layer suppressed harmful chemical reactions when holes were transferred to the electrolyte, as evidenced by electrochemical impedance spectroscopy (EIS) characterization. The HfO_2_/BiVO_4_ structured photoanode exhibits a cathodic onset potential shift of 0.28 V_RHE_ and improved charge transfer characteristics. Interestingly, the combination of HfO_2_ and NiPt protective layers on the BiVO_4_ photoanode achieves excellent long‐term stability over 800 h under V‐contained electrolyte condition. Overall, the above‐discussed metal oxide‐based protective materials offer several potential advantages and have proven their photocorrosion‐resistant effect; however, they face some limitations, such as poor electrical conductivity, parasitic light absorption, and susceptibility to photocorrosion.

#### 4.1.3 Metal‐Insulator‐Semiconductor‐Based Layer

**FIGURE 9:**
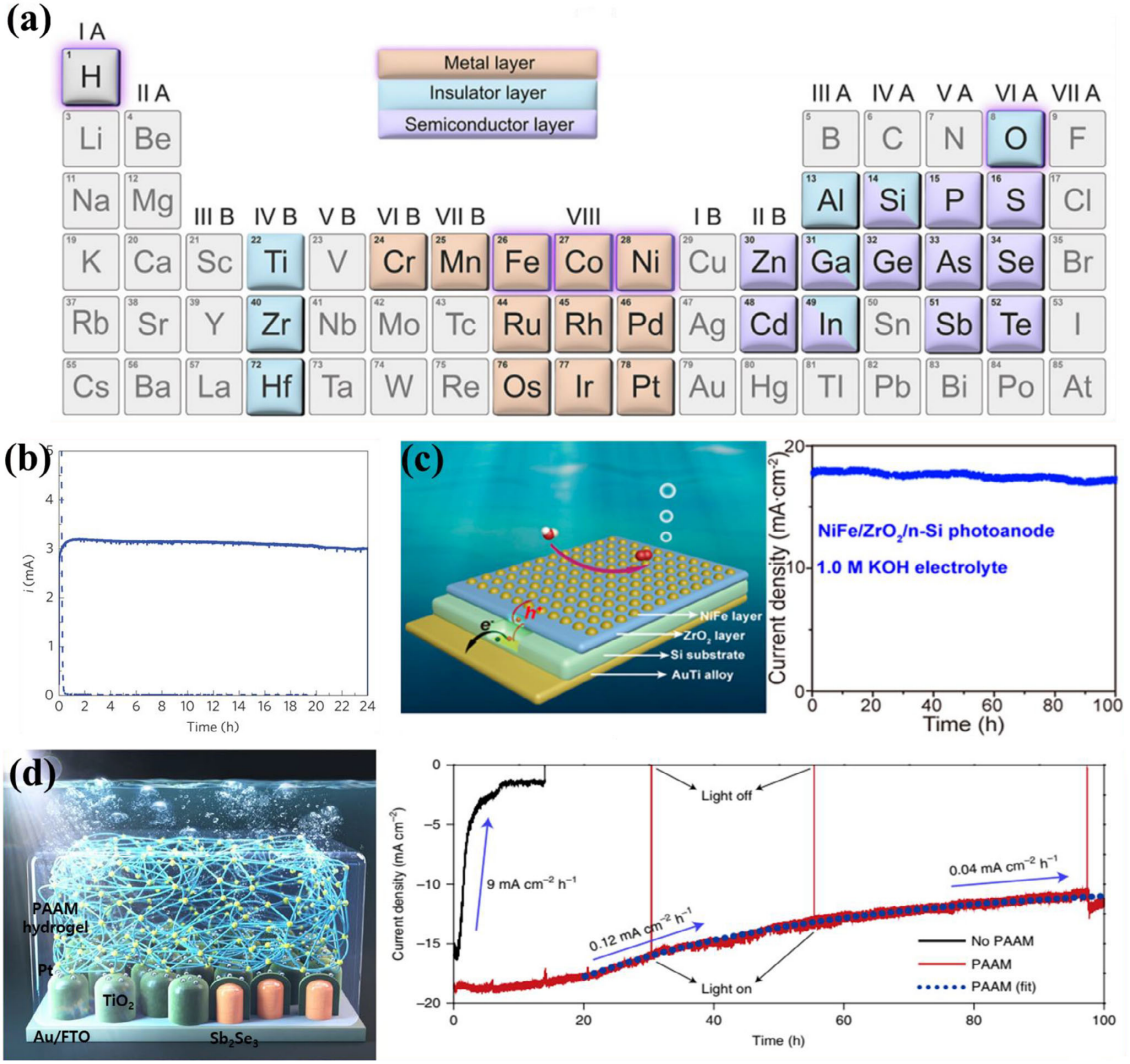
(a) Elements to prepare each layer of the MIS included photoelectrodes. Reproduced with permission [162]. Copyright 2024, Royal Society of Chemistry (RSC). (b) Long‐term stability for Ir/TiO_2_/Si‐based photoanode under constant potential at 1.7 V_NHE_ on a 0.196 cm^2^ and 1 M NaOH solution. Reproduced with permission [191]. Copyright 2011, Springer Nature. (c) PEC device scheme and long‐term stability for NiFe/ZrO_2_/*n*‐Si‐based photoanodes under alkaline conditions (pH = 14, 1.0 M KOH electrolyte). Reproduced with permission [193]. Copyright 2018, American Chemical Society. (d) Schematic and time‐dependent current density of Pt/TiO_2_/Sb2Se_3_ photocathodes without and with PAAM hydrogel‐protective layer under H_2_SO_4_ electrolyte (pH = 1). Reproduced with permission [194]. Copyright 2022, Springer Nature.

The long‐term stability of a photoelectrode can be improved by introducing conductors and/or insulators as a protective layer in connection with catalysts [160, 162, 186]. Charge transfer can be improved by conductors, while insulators protect the photoelectrode surface from photocorrosion and recombination at the photoelectrode/electrolyte interface [162, 187]. The utilization of conductors and/or insulators presents a promising approach to enhancing the long‐term stability and performance of PEC water splitting [44]. In this regard, the metal‐insulator‐semiconductor (MIS) structures are promising protective layers for PEC water splitting. Particularly, the insulator layer serves two purposes: (i) it shields the photoelectrodes from corrosive processes and (ii) it promotes the successful transfer of minority carriers across the MIS heterojunction [163, 188, 189]. The ideal insulator layer should possess exceptional thermodynamic and kinetic stability to ensure a long working life, optical transparency to reduce parasitic light absorption, tunable electronic properties, and an energy band structure that allows photogenerated carriers to be separated. Figure 9a shows well‐known elements for compounds in each layer for MIS photoelectrodes, and their complex interplay for each layer is essential for achieving long‐term stability for the PEC device. In this regard, the excellent reviews regarding the MIS protective layer on PEC devices, including basic concepts, fundamental principles, material for each MIS layer, classifications, charge carrier transfer mechanisms, and tandem MIS‐structured PEC applications, have been reported [8, 160, 162, 163, 190].

McIntyre and co‐workers [191] reported excellent corrosion‐resistant pinhole‐free Ir/2 nm thick ALD‐grown TiO_2_/Si‐based photoanode with excellent long‐term stability over 24 h at 1.7 V_NHE_ in 1 M NaOH, as shown in Figure 9b. Authors suggested that the Ir/TiO_2_/Si‐based MIS photoelectrode achieved higher photocurrent density and a larger photovoltage from 510 to 570 mV than previously reported metal oxide photoanodes. Interestingly, the photocurrent densities were dependent on the thickness of TiO_2_ and measured temperatures, indicating that it was related to the more thermally‐activated and bulk‐limited conduction mechanism, such as trap‐assisted tunneling or Frenkel‐Poole conduction [192]. Later, Liu and co‐workers investigated the significantly improved long‐term stability for Si‐based photoanode by an insulator layer (i.e., TiO_2_ or ZrO_2_) engineering strategy in MIS structured photoelectrode as shown in Figure 9c [193]. Particularly, the Si‐based MIS photoanode incorporating a high‐resistance ZrO_2_ as insulator exhibited excellent long‐term stability over 100 h under alkaline conditions (1.0 M KOH electrolyte and pH 14). NiFe/ZrO_2_/n‐Si structured photoanode showed high photocurrent density (26.6 mA/cm^2^ at 1.23 V_RHE_), low onset potential (0.96 V_RHE_), and improved OER activity. Additionally, authors proposed that the insulator oxide electroforming phenomenon was the main cause of the degradation mechanism for the Si‐based MIS photoanode during the long‐term stability test under constant external voltage. Recently, Moon and co‐workers [194] were the first to introduce polyacrylamide (PAAM) hydrogel as highly permeable and transparent protector on MIS‐based Sb_2_Se_3_ photocathodes (i.e., Pt/TiO_2_/Sb_2_Se_3_) with improved photocurrent density of ‐19 mA/cm^2^ at 0 V_RHE_ and long‐term stability over 100 h (maintaining ∼70% of the initial photocurrent density) as shown in Figure 9d compared to that without PAAM in a H_2_SO_4_ electrolyte (pH 1). In particular, the degradation rate for the PAAM/Pt/TiO_2_/Sb_2_Se_3_ structured photocathode (0.04–0.12 mA/cm^2^·h) was significantly reduced compared to that without PAAM (9 mA/cm^2^·h). Authors also demonstrated the structural stability mechanism for the Pt catalyst, which prevents agglomeration and suppresses the photocorrosion of TiO_2_ by stabilizing the dissolved Ti^3+^ ions in PAAM via Le Chatelier's principle. Additionally, they revealed an effective bubble escape strategy through micro gas tunnels in PAAM, which improved the photoelectrode's mechanical stability. Through a similar approach, the PAAM/NiFeO_x_/BiVO_4_ structured photoanode achieved excellent long‐term stability over 500 h at the KB_i_ electrolyte (pH 9) without significant degradation of photocurrent density. Although the MIS‐based photoelectrodes exhibited excellent long‐term stability, several drawbacks persist, such as high resistance from each interface, the need to discover novel materials for each layer to improve the photocurrent density and STH efficiency, developing advanced characterizations to clearly demonstrate and unravel the working mechanism, as well as large‐scale production.

#### 4.1.4 Amorphous Layer

Amorphous protective layers have become known as a promising strategy for improving the long‐term stability of photoelectrodes, which are critical components of PEC devices used for water splitting and solar fuel production [43, 160]. They are superior to conventional crystalline protective layers in several ways, including enhanced long‐term stability, high light transmission, large conformal coverage, defect passivation, and tunable properties [195, 196]. PEC systems can become more robust and efficient with continued advancements in the study of materials and fabrication processes by introducing amorphous protective layers on photoelectrodes [161].

**FIGURE 10:**
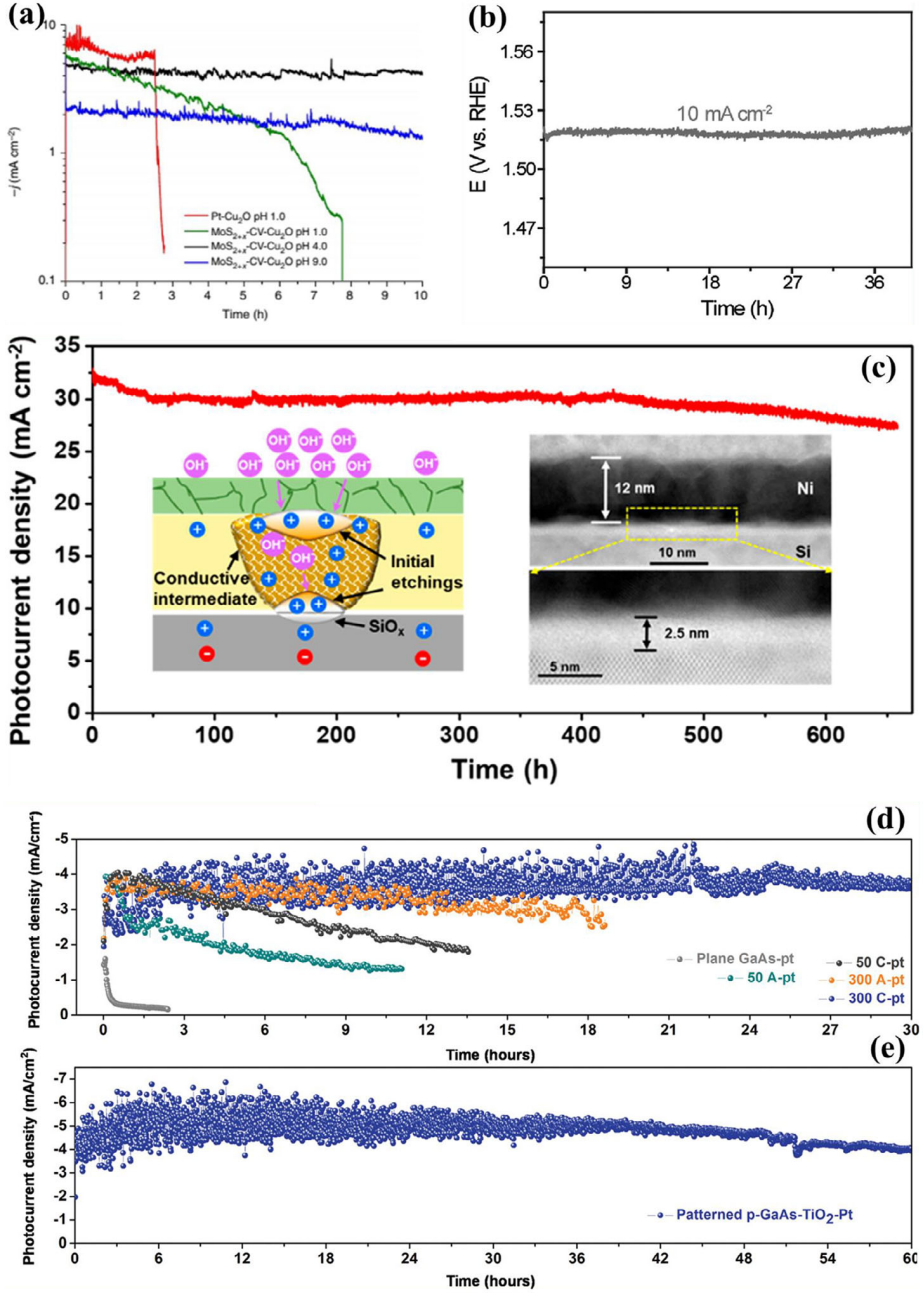
(a) Long‐term stability for *a*‐MoS_2‐x_/Cu_2_O structured photocathode at 0 V_RHE_ under AM 1.5 irradiation and pH 4. Reproduced with permission [103]. Copyright 2014, Springer Nature. (b) Long‐term stability for CoFe‐OH/BiVO_4_‐based photoanode under constant current density of 10 mA/cm^2^ under 1.0 M Na_2_SO_3_ as hole scavenger in a 0.5 M potassium phosphate buffer (pH 7). Reproduced with permission [198]. Copyright 2017, Wiley‐VCH. (c) Photocurrent density versus time for *a*‐TiO_2_/Si‐based photoelectrode measured in 1 M NaOH aqueous solution under 1 sun illumination with an external bias of 1.8 V_RHE_. The inserted figures are a schematic of the failing mechanism of *a*‐TiO_2_ and STEM images for Ni/*a*‐TiO_2_/Si structured photoelectrode. Reproduced with permission [199]. Copyright 2018, American Chemical Society. Long‐term stability results for (d) GaAs‐based photoelectrodes with various TiO_2_ protective layers and (e) 26 nm TiO_2_‐coated patterned GaAs‐based photoelectrode at 0 V_RHE_ under illumination conditions. Reproduced with permission [200]. Copyright 2020, Wiley‐VCH.

Hu and co‐workers demonstrated a simple and scalable approach for preparing *a*‐MoS_2‐x_ protective layer as a hydrogen evolution catalyst onto a protected Cu_2_O photocathode [103]. The *a*‐MoS_2‐x_/TiO_2_/AZO/Cu_2_O structured photocathode exhibited a photocurrent density of ‐5.7 mA/cm^2^ at 0 V_RHE_ and the excellent long‐term stability over 10 h at pH 1 under simulated AM 1.5G solar illumination, as shown in Figure 10a. Furthermore, authors demonstrated the charge transfer mechanism through the band energy diagram for *a*‐MoS_2‐x_/TiO_2_/AZO/Cu_2_O photocathode, assuming band edge pinning at the interfaces and taking the built‐in potentials at interfaces equal to the difference in Fermi level. Jang and co‐workers reported a facile and large‐scale approach for electrodepositing *a*‐CoO_x_ NW as a protective layer on a Si photoanode [195]. The *a*‐CoO_x_/Si‐structured photoanode exhibited the large onset potential of 1.06 V_RHE_, photocurrent density of 23.3 mA/cm^2^ at 1.23 V_RHE_, charge injection efficiency of 100% at 1.4 V_RHE_, high external quantum efficiency of 90% at 700 nm as well as excellent long‐term stability over 200 h at 1.8 V_RHE_ (80% of the initial photocurrent density) in K‐borate electrolyte (pH = 9.5). A meaningful study by Mullins and co‐workers investigated how *a*‐FeOOH on a Si photoelectrode has been used as both a protective layer and a catalyst for OER [197]. Authors revealed that the *a*‐FeOOH protective layer exhibited several beneficial characteristics, such as: (i) highly transparent properties in the visible region; (ii) excellent coverage in the electrolyte; and (iii) the capacity to function well over a range of electrolyte pH values. The *a*‐FeOOH/Si structured photoanode achieved excellent long‐term stability over 4 h without significant degradation, as well as STH efficiency of 4.3% in 1 M Na_2_CO_3_ at 0 V_RHE_. Similarly, Jin and co‐workers introduced a facile electrodeposition of *a*‐CoFe‐H nanosheets as both an electrocatalyst and protective layer for BiVO_4_‐based photoanode [198]. Interestingly, *a*‐CoFe‐H nanosheets offered high electrochemically active surface area, high transparency, and excellent OER catalytic activity with low overpotential (280 mV at 10 mA/cm^2^) and Tafel slope (28 mV/dec) and long‐term stability over 40 h in alkaline electrolyte as shown in Figure 10b. The *a*‐CoFe‐H/BiVO_4_ structured photoanode showed significantly enhanced photocurrent density of 2.48 mA/cm^2^ at 1.23 V_RHE_ compared to the bare photoanode (∼0.6 mA/cm^2^ at 1.23 V_RHE_). Unfortunately, the long‐term stability of *a*‐CoFe‐H/BiVO_4_ structured photoanode has not been reported.

As per studies, it is a well‐known fact that *a*‐TiO_2_ plays a crucial role as an effective protective layer [92, 176]. Considering this, Wang and co‐workers [199] successfully developed *a*‐TiO_2_ as an ultra‐stable protection layer on a Ni/a‐TiO_2_/Si‐structured photoanode. This strategy could improve the photocurrent density over ‐30 mA/cm^2^ at 0 V_RHE_ and excellent long‐term stability over 600 h with little photocurrent density degradation at 1 M NaOH aqueous solution under 1 sun illumination conditions, as shown in Figure 10c. Interestingly, the authors revealed the potential degradation and photocorrosion mechanisms using detailed TEM characterization and the surface damage phenomenon by FE‐SEM images for the photoelectrode under long‐term stability test. On the other hand, Zhang and co‐workers [200] reported an important role for *a*‐TiO_2_ in improving the long‐term stability of a large‐area patterned microdome structured *p*‐GaAs photocathode. It is well studied that the outstanding ALD‐prepared *a*‐TiO_2_ is a protective material on the photoelectrode with excellent long‐term stability for PEC devices. Authors simulated the optical studies for microdome structured *p*‐GaAs with *a*‐TiO_2_ to optimize the light absorption behavior. The *p*‐GaAs structured photocathode with different *a*‐TiO_2_ thicknesses exhibited significantly improved photocurrent density of ‐5 mA/cm^2^ under ‐0.8 V_Ag/AgCl_ and excellent long‐term stability over 60 h with only 19.8% degradation initial photocurrent density as shown in Figures 10d,e in a neutral pH electrolyte. Authors suggested that the enhanced charge separation was attributed to the favorable band alignment between *p*‐GaAs and *a*‐TiO_2_.

By applying amorphous protective layers to photoelectrodes, researchers have achieved significant progress in improving their long‐term stability and longevity. This is primarily owing to their better homogeneity and flawless coverage over the photoelectrode surface compared to their crystalline counterparts. This homogeneity successfully prevents material degradation by providing all‐around protection against the corrosive conditions encountered during PEC processes. However, there are also some demerits to using an amorphous protective layer, such as: (i) poor adhesion between the amorphous and photoelectrodes (i.e., peeling off), (ii) sensitive photoactivity of the amorphous layer (i.e., generating parasitic current density), and (iii) poor electrical property (i.e., leading to high series resistance). Optimizing the layer's thickness is one of the challenging issues, though, since excessively thick coatings can obstruct charge flow and lower PEC performance. To combine protection with ensuring the effective flow of electrons, careful material selection, fine interface engineering, and deposition processes at low temperatures are needed.

### 4.2 Interface and Junction Engineering

There are several advantages of designing a highly efficient photoelectrode decorated with a catalyst and protective layer to significantly improve PEC performance [160, 201]. By passivating the surface dangling bonds, defects, and related defect clusters, multiple insulator semiconductors with strong dielectric characteristics are applied to the photoelectrode surface to reduce the surface recombination rate. Additionally, effective charge transfer and overall system performance depend on a robust bond between the catalyst and the protective layer, which is minimized at lower levels and preserves a stable and reliable interface [202, 203]. However, the multilayered catalysts/insulator/photoelectrode device exhibited poor adhesion at each interface during long‐term operations. Enhanced adhesion efficiently protects the catalyst and photoelectrode from corrosive environments by ensuring that the protective layer persists under the demanding operating conditions. This strong interface reduces the possibility of chemical and physical deterioration, extending the PEC system's useful life and preserving its efficiency [160, 204]. In this regard, several strategies have been employed, such as: (i) introducing a functional layer between the catalyst and protective layer, (ii) optimizing deposition approach to prevent delamination, (iii) multi‐layered protection strategy, (iv) surface modifications for photoelectrode or catalysts, and (v) catalyst encapsulation strategy [42, 160]. In this section, the catalyst encapsulation strategy is mainly presented. The introduction of the functional layer between the catalyst and protective layer, the multi‐layered protection strategy, the optimized deposition approach, and surface modification are discussed.

**FIGURE 11:**
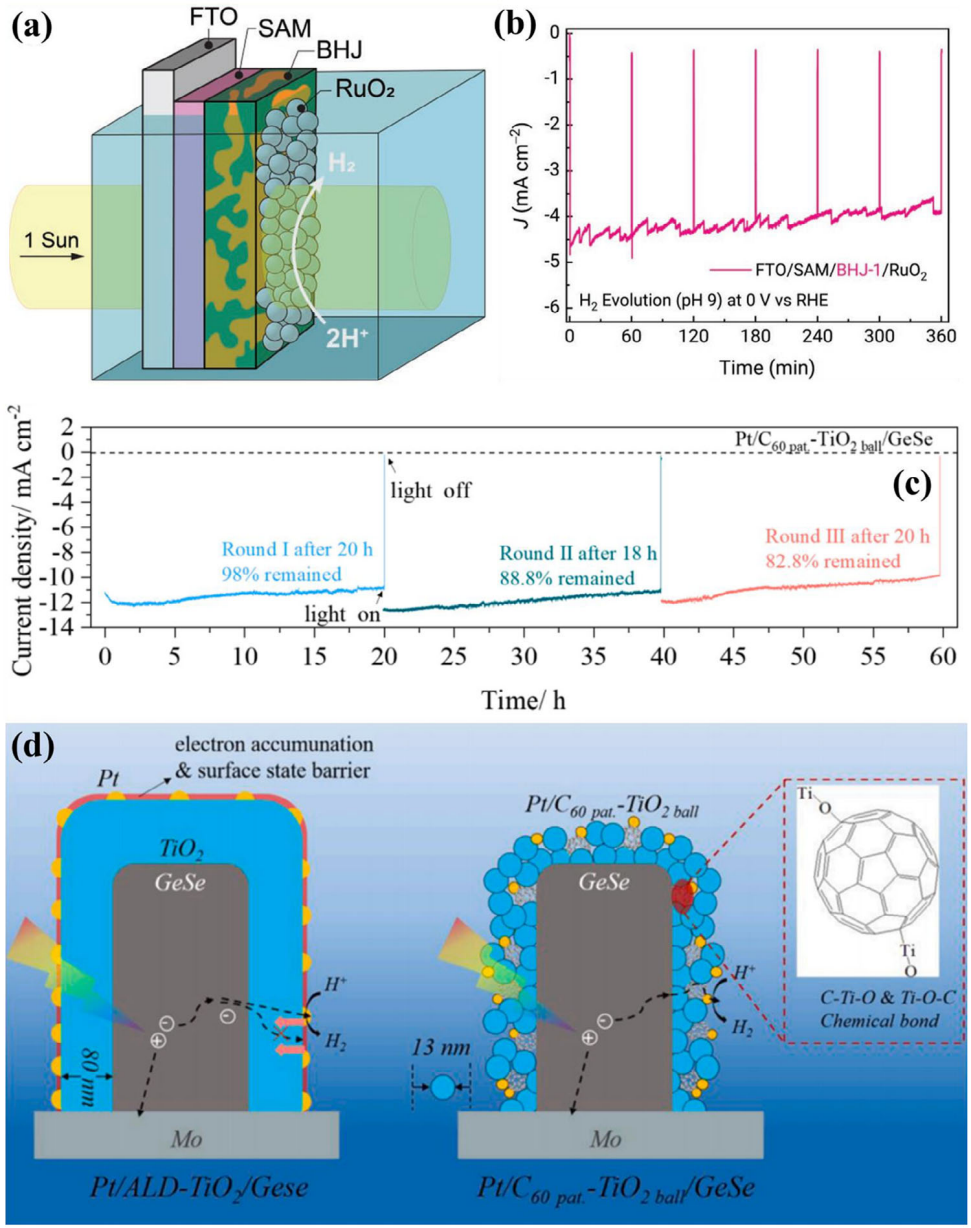
(a) Scheme and (b) long‐term stability results of FTO/SAM/BHJ/RuO_2_ structured photocathode at 0 V_RHE_ in 0.1 M NaB_i_ buffer electrolyte (pH 9). Reproduced with permission [211]. Copyright 2022, Wiley‐VCH. (c) Long‐term stability test and (d) schematic illustration for electron charge transfer mechanism of Pt/C_60_/TiO_2_/GeSe at 0 V_RHE_ in electrolyte (pH = 6.8). Reproduced with permission [212]. Copyright 2021, Elsevier.

As is well known, encapsulating the catalysts with a protective layer (i.e., metal oxides or organic semiconductors) can prevent detachment and agglomeration to improve the structural stability of catalysts, and a similar approach has been widely investigated in photoelectrodes [194, 205, 206, 207, 208, 209, 210]. Interestingly, organic semiconductors offer an adjustable energy level, easily controllable molecular engineering, excellent optoelectronic properties, as well as scalable production using a solution‐based process [14]. Sivula and co‐workers successfully introduced the organic semiconductor to enhance adhesion materials between catalysts and photoelectrode on bulk heterojunction (BHJ)‐based Si photoelectrode, as shown in Figure 11a [211]. Authors selected an organic self‐assembled monolayer (SAM) as an enhancing adhesion material between catalysts and photoelectrode owing to its benefits, such as easily controllable chemical functionality, extremely consistent and flawless (i.e., good adhesion property), versatility, and facile preparation process. As a result, the RuO_2_ catalyst/BHJ/SAM/FTO structured photoelectrode exhibited the improved photocurrent density of −4.6 mA/cm^2^ at 0 V_RHE_, positive onset potential of a 1.0 V_RHE_ and significantly improved long‐term stability over 6 h maintaining 90% of the initial photocurrent density in 0.1 M NaB_i_ buffer electrolyte (pH 9) under a 1 sun irradiation condition as shown in Figure 11b.

On the other hand, Jiang and co‐workers successfully introduced C_60_ as an enhancing adhesion and electron transfer material between Pt catalysts and TiO_2_ balls on GeSe photocathodes [212]. Authors suggested that the incorporation of C_60_ could improve the adhesion between catalysts and the protective layer, facilitate electron transfer, and reduce photoexcited charge carriers at interfaces. As a result, Pt/C_60_/TiO_2_/GeSe structured photocathode showed the improved photocurrent density of −9.6 mA/cm^2^ at 0 V_RHE_, a positive onset potential of 0.6 V_RHE_ and significantly improved long‐term stability over 60 h (∼80% of the initial photocurrent density) in a phosphate buffer electrolyte (pH 6.8) under simulated sunlight AM 1.5 G conditions as shown in Figure 11c. Authors proposed that (i) the electron transfer pathway in Pt/C_60_/TiO_2_/GeSe structured photocathode was shorter and (ii) electrons were trapped through the thick TiO_2_ protective layer as shown in Figure 11d. The trapped electrons on the surface led to an electric field and prevented the HER process, while electron accumulation is effectively suppressed owing to fast charge carrier transfer out of the TiO_2_ layer through C_60_ to the Pt catalyst. A similar approach has been reported using an organic semiconductor as an enhancing adhesion material in various photoelectrodes by Moon's group [136, 194]. Changing the surface morphology and employing organic solvent‐based approaches are two efficient ways to alter photoelectrodes in PEC water splitting. Organic solvents improve the light absorption, charge separation, and catalytic activity of photoelectrode surfaces by facilitating the deposition of thin films, nanostructures, or catalysts. Photoelectrodes can be made more efficient by modifying their morphology, such as by adding rough surfaces, porous frameworks, or various nanostructures [6, 213, 214]. A large surface area of a photoelectrode can offer excellent wettability for the electrolyte. Additionally, flexibility and versatility also help improve charge transfer, reduce recombination rate at the interface, and enhance long‐term stability of PEC devices.

**FIGURE 12:**
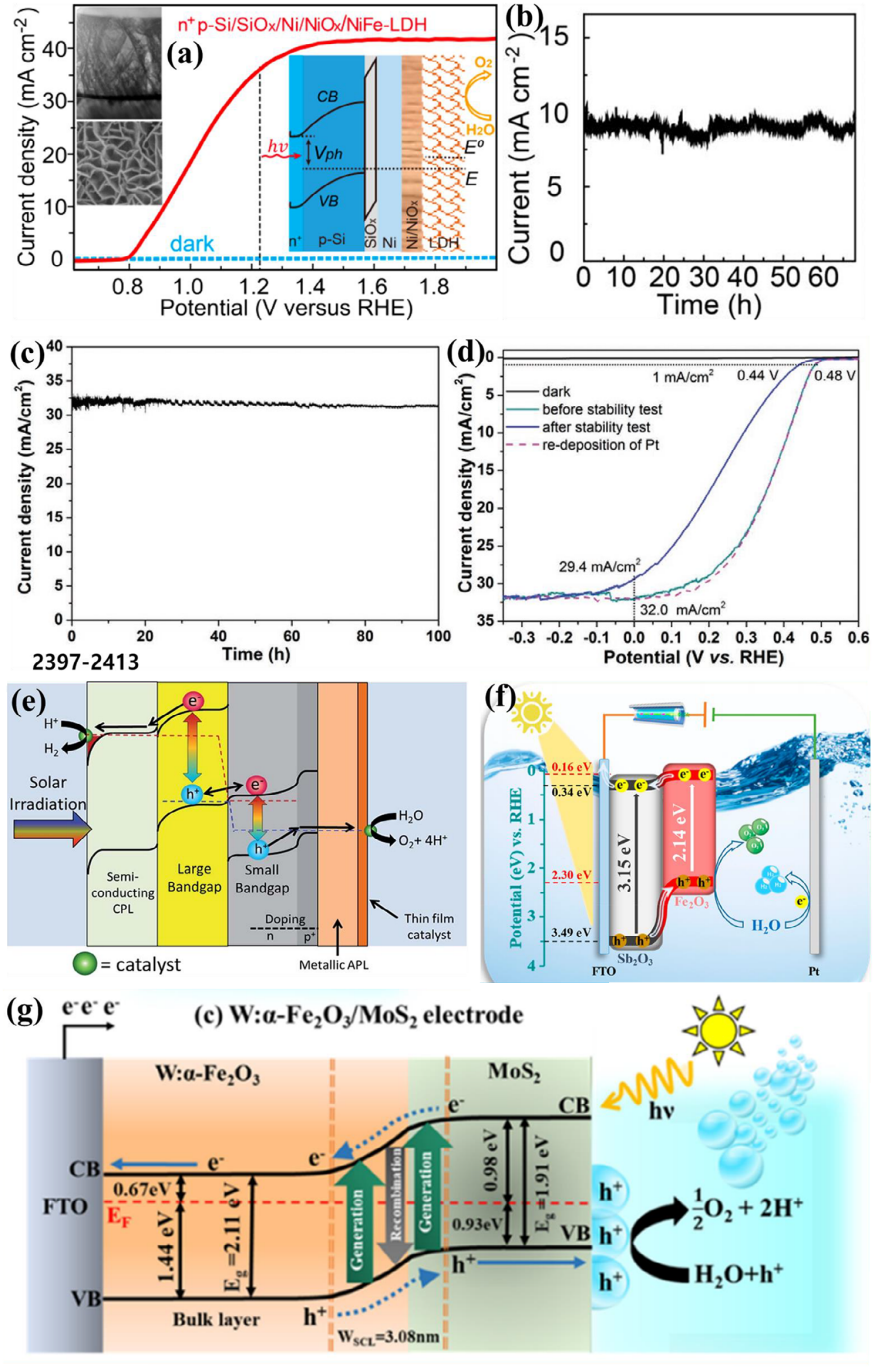
(a) *J*–*V* curves and (b) long‐term stability for *n^+^p*‐Si/SiO_x_/Ni/NiO_x_/NiFe‐LDH photoanode under dark and illumination in 1.0 M KOH (pH = 13.6) electrolyte solution. The insert figures are cross sectional TEM and surface FE‐SEM images for NiFe‐LDH on photoelectrodes, and schematic band alignment for *n^+^p*‐Si/SiO_x_/Ni/NiO_x_/NiFe‐LDH photoanode for water oxidation. Reproduced with permission [221, 238]. Copyright 2018, American Chemical Society. (c) Long‐term stability and (d) *J*–*V* curves for Pt/Ag/Si structured photocathodes without and with long‐term stability at 0 V_RHE_ under 100 mW/cm^2^ illumination in 0.5 M H_2_SO_4_. Reproduced with permission [222]. Copyright 2018, Wiley‐VCH. (e) Scheme for band alignment, charge separation, and transfer in a multi‐layered structure photoelectrode. Reproduced with permission [232]. Copyright 2014, Royal Society of Chemistry. (f) Schematic mechanism for Sb_2_O_3_/Fe_2_O_3_‐based photoanode for water oxidation. Reproduced with permission [233]. Copyright 2022, American Chemical Society. (g) Schematic illustration for charge separation, transfer, and band alignment for W:*a*‐F_2_O_3_/MoS_2_ photoelectrode. Reproduced with permission [235]. Copyright 2021, American Chemical Society.

Traditionally, the HTL, ETL, and co‐catalysts serve as multifunctional materials (i.e., improving charge transfer, reducing the recombination layer, and suppressing chemical corrosion reaction at photoelectrodes) between the protective coating and catalysts have been well known to improve performance as well as long‐term stability of PEC devices [4, 215, 216]. Interestingly, surface modification of the multifunction materials to minimize surface roughness can lead to further decreased absorption and reduced light scattering at photoelectrodes [217, 218]. In this regard, the performance and long‐term stability of a PEC water‐splitting system can be greatly affected by the choice of HTL, ETL, and co‐catalyst materials. The role of each multifunction material, a detailed discussion for excellent investigation results, and remaining challenges have been reported [217, 219, 220]. Gong and co‐workers [221] investigated facile integration between *n* ^+^ *p*‐Si and catalysts (NiFe‐layered double hydroxide (LDH)) for photoanodes by a multifunctional bridging layer by introducing a partially activated Ni (Ni/NiO_x_) to improve the performance and long‐term stability of a PEC device. In this device structure, Ni metallic interlayer protects the Si‐based photoanode from photocorrosion and enhances adhesion between Si and NiO_x_, indicating that it has a high capacity of hole accumulation and strong bonding with NiFe‐LDH. The *n* ^+^ *p*‐Si/SiO_x_/Ni/NiO_x_/NiFe‐LDH structured photoanode showed an excellent photocurrent density of 37 mA/cm^2^ at 1.23 V_RHE_, an onset potential of 0.87 V_RHE_, and good reproducibility over 20 samples as shown in Figure 12a at 1.0 M KOH electrolyte solution (pH = 13.6). Interestingly, this photoanode also exhibited outstanding long‐term stability over 68 h at a constant photocurrent density of 9 mA/cm^2^ at 1.23 V_RHE_ without degradation, as shown in Figure 12b. Authors suggested that the properties of NiFe‐LDHs were not changed based on FE‐SEM images after the long‐term stability test. This bridging layer approach is effective and facilitates charge transfer and establishes strong contact at the semiconductor/electrocatalyst interface in solar water‐splitting devices.

Pan and co‐workers [222] successfully demonstrate the introduction of a spatial decoupling multifunctional layer for water splitting. Here, SiN_x_ and Ag/Pt played the role of antireflection, passivating defects on the surface, protecting against photocorrosion, and serving as catalysts on *p‐n* ^+^Si‐based photoelectrodes. Interestingly, authors found that the electron‐tunneling glass layer between SiN_x_ and *n* ^+^‐Si layers offers good long‐term stability to Si‐based photocathodes in an acidic electrolyte (0.5 M H_2_SO_4_). As a result, the Pt/Ag/Si structured photocathode exhibited the improved photocurrent density for −36.1 mA/cm^2^ at 0 V_RHE_, a large onset potential of 0.61 V_RHE_, and high ABPE exceeding 9.7%. This photocathode also exhibited excellent long‐term stability over 100 h without significant photocurrent density degradation, as shown in Figure 12c,d. After re‐deposition of Pt on a Si‐based photocathode after long‐term stability test over 100 h, the photocurrent density recovered to a similar value to that before long‐term stability test (−32 mA/cm^2^ at 0 V_RHE_), indicating that the main cause of degradation was strongly related to the detachment of Pt during long‐term stability test.

Considering the above‐discussed points related to the introduction of multifunctional layers (e.g., ETL, HTL, protective, and co‐catalyst layers) in PEC water‐splitting devices, it is necessary to mention that selecting the appropriate HTL and ETL materials for a PEC water‐splitting device is paramount, as it profoundly affects its effectiveness and long‐term stability. These approaches to improve the long‐term stability of the PEC water‐splitting devices are the inclusion of a multifunctional material between the protective layer and the catalyst. This interlayer in PEC water‐splitting devices plays critical roles such as enhancing adhesion, promoting charge transfer, and offering corrosion protection. As a result, it is critical to examine the device's distinctive requirements in order to achieve exceptional performance and long‐term stability while selecting materials for PEC water‐splitting devices. However, there are some drawbacks such as parasitic absorption, reflection loss, alignment E_g,_ and/or band structure alignment for each layer, and high series resistance when employing multifunctional materials on photoelectrodes [217, 218, 220, 223, 224, 225, 226].

A photoelectrode devices performance in efficiently converting sunlight into photocurrent density depends on its band alignment between the photoelectrodes and the co‐catalyst or prospective layer [227]. The E_g_ of the photoelectrode should be such that it allows light in the intended wavelength range to be absorbed [228]. The VB position of the photoelectrode should be more negatively oriented than the electrolyte's oxidation potential, and its CB position should be more positively oriented than the electrolyte's reduction potential [229]. The phenomenon related to the reduction of a species in the electrolyte guarantees that electrons were stimulated from the VB to the CB and then injected into the electrolyte solution. The effectiveness of an outer barrier in preventing deterioration of the photoelectrode also relies on its band alignment [230]. The heterojunctions prepared with the two semiconductors can be categorized into three types based on their energy band alignments: straddling‐gap heterojunctions (type‐I), staggered‐gap heterojunctions (type‐II), and broken‐gap heterojunctions (type‐III) [231].

Depending on the application and intended device performance, each type of heterojunction has unique benefits and drawbacks. To maximize device performance, long‐term stability, and functionality, these criteria must be balanced while selecting the right heterojunction type. In summary, Type‐II heterojunctions are useful for PEC devices because they provide effective charge separation and decrease recombination, hence improving photocatalytic effectiveness. Despite the difficulties of material selection and band alignment, Type III broken‐gap heterojunctions improve carrier confinement and allow for new PEC device architectures.

Chorkendorff and co‐workers successfully investigated the band alignment, charge separation, transfer, and protective layers in tandem PEC devices [232]. They analyzed device design parameters such as (i) the selection of suitable photoelectrodes, including photoanode and/or photocathode, (ii) light absorption properties of the redox catalysts, (iii) electrolyte or operating pH, and (iv) the introduction of suitable protective materials on the photoelectrode. As a result, they designed an effective tandem device structure with a protective photocathode, *p‐n* homojunction photoanode, metallic protective layer, and catalysts as shown in Figure 12e. Additionally, authors discussed the representative materials for each layer and the favorable operating pH in redox reactions. Chen and co‐workers [233] first demonstrated the formation of a type II heterojunction structure consisting of Sb_2_O_3_ and Fe_2_O_3_ for significantly enhanced PEC water oxidation [234]. The Sb_2_O_3_/Fe_2_O_3_ photoanode exhibited a remarkably improved photocurrent density of 1.31 mA/cm^2^ at 1.23 V_RHE_ in 1 M KOH (pH 13.97), almost 14.5 times that of bare Fe_2_O_3_ and an outstanding ABPE of 0.08% (10.7 times that of Fe_2_O_3_). Surprisingly, this photoelectrode achieved excellent long‐term stability over 20 h without rapid degradation. The band alignment between Sb_2_O_3_ and Fe_2_O_3_ was proposed, and they formed a type II heterojunction, which promoted charge separation and transfer as shown in Figure 12f. Authors found improved device performances and excellent long‐term stability were mainly caused by the following reasons: (i) increased contact area between photoanode and electrolyte, and (ii) the formation of a type II heterojunction. Lee and co‐workers [235] employed a strategy for improving PEC performance by introducing the formation of a type II heterojunction with a facile approach of W‐doped a‐Fe_2_O_3_/MoS_2_ core‐shell structured photoelectrode. According to their detailed investigations, the MoS_2_ prepared by the liquid‐phase exfoliation process with few‐layer nanosheets had an average thickness of about 6 nm. The W‐doped *a*‐Fe_2_O_3_/MoS_2_ photoanode achieved an improved photocurrent density of 1.83 mA/cm^2^ at 1.23 V_RHE_, IPCE of 37% and ABPE of 0.26% under 100 mW/cm^2^ illumination conditions in 1 M NaOH electrolyte. Through extensive characterizations, authors proposed the charge separation, transfer mechanisms, and band alignment for approached W‐doped *a*‐Fe_2_O_3_/MoS_2_ photoanode as shown in Figure 12g. Similarly, Zhu and co‐workers [236] studied the band alignment between *a*‐FeO_x_ and Co_3_O_4_ in the Fe_2_O_3_ photoanode to investigate systematically the recombination at each interface. They suggested the charge separation and transfer mechanisms using band alignments. (i) When Fe_2_O_3_ encounters Co_3_O_4_, the diffusion and drift of carriers occur, resulting in a thermodynamic equilibrium of the Fermi levels. (ii) Photogenerated holes can separate from electrons, preventing recombination. These holes then move to catalytic sites for oxygen evolution. This occurs due to the upward bending of Fe_2_O_3_ energy bands from the bulk to the interface, which creates an internal electric field. Beneficial band alignment guarantees that photogenerated charge carriers are properly utilized for the needed PEC reaction by avoiding photocorrosion, mitigating charge recombination, improving transfer of charge, and encouraging surface passivation, all of which result in increased device efficiency and long‐term stability [237].

In summary, interface and junction engineering has become a highly effective approach for simultaneously enhancing both the efficiency and long‐term stability of PEC water‐splitting systems. In contrast to protective passivation, which mainly aims to prevent photocorrosion, interface engineering focuses on optimizing the interactions among the photoelectrode, protective layer, charge‐transport layer, and electrocatalyst. This integrated approach enables efficient charge extraction while preserving strong interfacial adhesion.

The use of well‐designed interlayers, multifunctional charge‐transport materials, and semiconductor heterojunctions can significantly improve charge separation, accelerate interfacial charge transfer, and reduce carrier recombination. At the same time, these strategies enhance the mechanical robustness of multilayer photoelectrodes during prolonged operation. Proper band alignment at semiconductor interfaces further supports this process by creating favorable charge‐transfer pathways and internal electric fields that promote carrier separation and suppress recombination losses.

Recent progress has therefore moved beyond simple protective coatings toward more integrated interface architectures, where each layer serves multiple roles, such as improving adhesion, facilitating charge transport, enhancing catalytic activity, and providing corrosion resistance. In the above section, key interface‐engineering strategies,including adhesion‐promoting interlayers, multifunctional transport layers, and heterojunction and band‐alignment design‐with a focus on their underlying mechanisms for improving PEC performance and durability have been discussed thoroughly.

### 4.3 Electrolyte Engineering

By changing the semiconductors, regulating the electrolyte compositions, or applying protective layers, the adverse chemical dissolution rate during PEC activity might be minimized [239]. A basic constraint on the lifetime of the PEC water splitting devices is strongly related that photons for energy harvesting are imposed by photocorrosion. In this regard, several factors, such as composition, addition of elements to the photoelectrode, pH, and reactivity of electrolytes, are strongly related to photocorrosion in photoelectrodes [41, 47, 162, 240]. In this section, the strategy of element addition to photoelectrodes is mainly presented to suppress the dissolution of the photoelectrode during the operation process.

**FIGURE 13:**
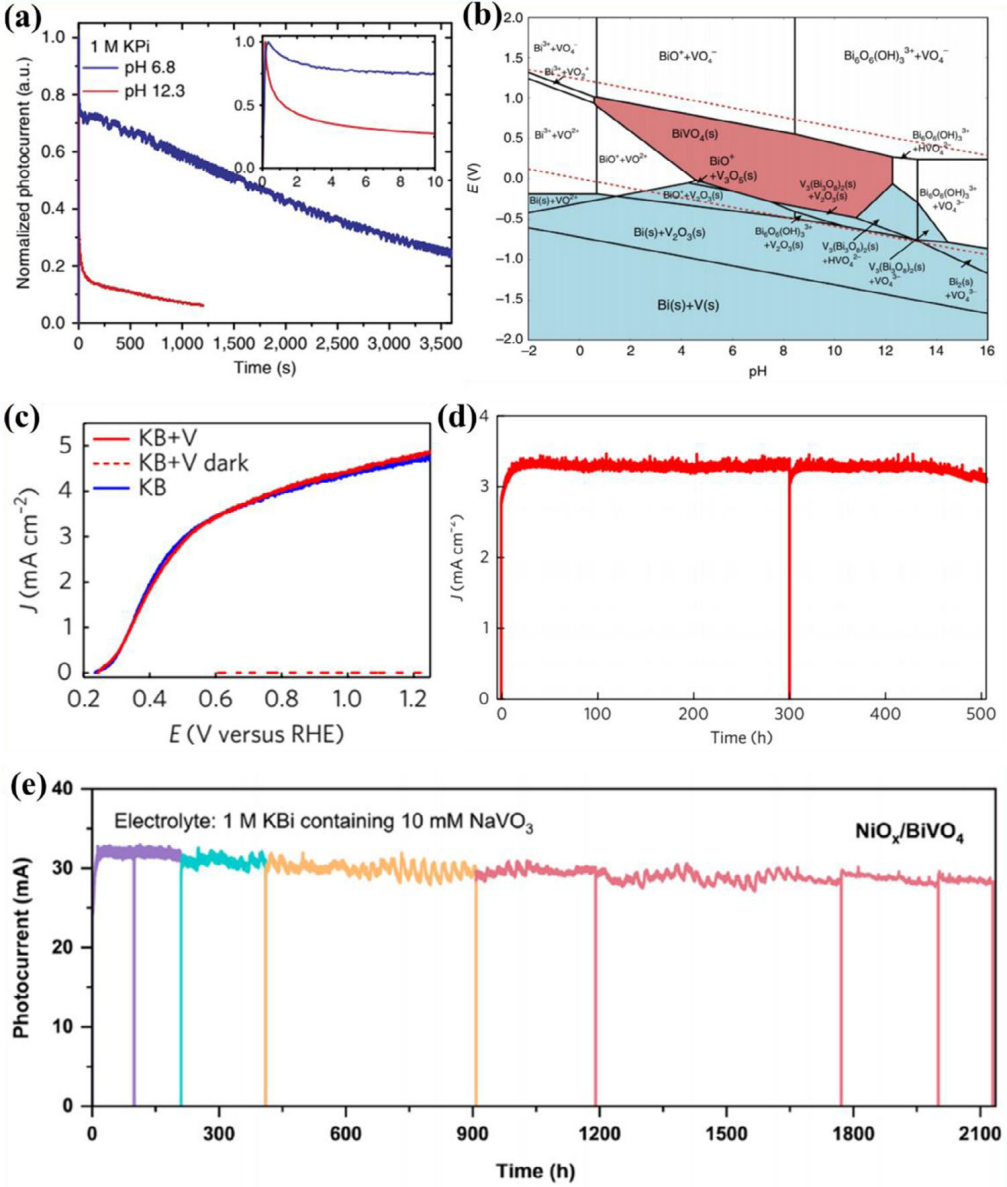
(a) Long‐term stability of pristine BiVO_4_ photoanode in 1 M sulfite at pH 6.8 (blue curve) and pH 12.3 (red curve) at 1.23 V_RHE_. (b) a Pourbaix diagram of 50%‐50% Bi‐V system in aqueous solution. The upper red dashed line shows the potential for OER, whereas the lower red dashed line corresponds to the potential for the HER. The cyan regions denote stable solid compounds, while the pink region is BiVO_4_. In the white regions, only ions are stable in aqueous solution. Reproduced with permission [244]. Copyright 2014, Springer Nature. (c) *J*–*V* plots and (d) long‐term stability for BiVO_4_/FeOOH/NiOOH in V^5+^ saturated 1 M potassium borate solution (pH 9.3) under AM 1.5G, 100 mW/cm^2^ illumination. Reproduced with permission [157]. Copyright 2018, Springer Nature. (e) Long‐term stability for BiVO_4_/NiO_x_ in 1 M KBi with 10 mM NaVO_3_ electrolyte. Reproduced with permission [245]. Copyright 2023, American Chemical Society.

BiVO_4_ is a well‐known photoanode material; however, it suffers significant photocorrosion during the OER process [241]. As per the literature [220, 242], the release of Bi^3+^ and V^5+^ ions into the electrolytes results from photogenerated holes attacking the BiVO_4_ crystal lattice, which causes dissolution. In this regard, Sugihara and co‐workers [243] first reported the degradation phenomenon of photocurrent density in BiVO_4_‐based photoanodes. They introduced an AgNO_3_ aqueous solution as the electrolyte, and the increased photocurrent density was observed. Ag^+^ ions can mitigate the dissolution of Bi^3+^ ions in BiVO_4_‐based photoanode; however, their long‐term stability was not notably enhanced. After some time had passed, Sharp and co‐workers revealed the cause of the degradation mechanism through advanced characterization tools (i.e., in situ electrochemical approach techniques) [244]. As a result, the BiVO_4_ photoanode showed higher photocurrent density at pH 6.8 than that at pH 12.3, while its long‐term stability was poor at pH 12.3 compared to that at pH 6.8, as shown in Figure 13a in 0.1 M sodium sulfite. Various characterizations for BiVO_4_‐based photoanodes both without and with long‐term stability suggested that (i) crystal facets of grains are not sensitive to chemical corrosion and (ii) corrosion rate is strongly related to the local/electrolyte interface morphology. Additionally, the chemical instability is not predicted from thermodynamic considerations of stable solid oxide phases such as Bi_2_O_3_ and Bi_4_O_7,_ as shown in Figure 13b. Authors suggested that the main cause for the dissolution of the BiVO_4_‐based photoanode was attributed to the limited structural transformation of the V‐deficient degradation product into a stable Bi‐based oxide phase. Following the above‐mentioned degradation mechanism of BiVO_4_‐based photoanode, Choi and coworkers [157] reported a remarkable long‐term stability on BiVO_4_‐based photoanode in KB_i_ solution at AM 1.5G, 100 mW/cm^2^ illumination condition. The nano‐porous BiVO_4_/FeOOH/NiOOH structured photoanode exhibited a highly improved photocurrent density of 5 mA/cm^2^ at 1.23 V_RHE_ and a similar photocurrent density after long‐term stability over 60 h in V‐containing KB_i_ electrolyte. Authors demonstrated that the use of a V^5+^‐saturated electrolyte can be an inexpensive and effective means to stop the anodic photocorrosion of BiVO_4_. The BiVO_4_‐based photoanode showed excellent long‐term stability over 500 h at 0.6 V_RHE_ and a photocurrent density of 3 mA/cm^2^ under V^5+^‐saturated electrolyte, as shown in Figure 13c,d. Recently, Wang and co‐workers [245] demonstrated the further improved long‐term stability of BiVO_4_‐based photoanodes for more than 2100 h, as depicted in Figure 13e, with the NiO_x_ catalyst layer deposited using the ALD techniques. Along with that, to improve the PEC device performance, they added NaVO_3_ to the aqueous medium electrolyte (pH 9.5). As a result, this approach helped to substantially improve the performance and long‐term stability of the BiVO_4_ photoanode. The addition of NaVO_3_ to the electrolyte can suppress photocorrosion of the photoanode and significantly improve the device performance (photocurrent density of 4.5 mA/cm^2^ at 1.23 V_RHE_). Authors revealed that the photoinduced holes are efficiently captured and stored by the NiO_x_ layer, which also accelerates the outer layer's OER dynamics and stabilizes the Bi─O bonding. It clearly suggests that the underlying reasons for poor long‐term stability for the photoanode during operation are intrinsic and photoaccelerated chemical corrosion reactions. Surface Bi sites tend to trap photoinduced holes and weaken Bi─O bonds when combined with nearby O atoms at VO_4_^3−^ in the BiVO_4_ lattice. Above‐discussed works have well established a new strategy for utilizing an electrolyte that is V^5+^‐saturated to inhibit the anodic photocorrosion of BiVO_4_. Similar approaches for BiVO_4_‐based photoelectrodes with significantly improved long‐term stability have been reported using V^5+^‐saturated electrolyte [245, 246]. To further improve the long‐term stability of the PEC water‐splitting device, precise electrolyte monitoring is essential [4, 160]. Proper electrolyte engineering can reduce corrosion, prevent unwanted reactions, and minimize harmful intermediates that can easily degrade photoelectrodes. For example, buffering the electrolyte to keep its pH constant guarantees a steady reaction environment and protects fragile elements from dissolving [247]. To stabilize the PEC system even more, substances that improve ionic conductivity or inhibit corrosion can be incorporated [248]. Thus, maintaining good performance and increasing the operational lifetime of PEC cells‐which increases the long‐term stability for continuous H_2_ production‐requires effective electrolyte management.

During PEC water splitting, *in*‐situ formed catalysts offer a promising strategy for fabricating long‐lasting and effective photoelectrodes due to their ability to be tailored to the specific photoelectrode material and are more stable than *ex*‐situ formed catalysts [249]. Precursor materials for catalysts are usually deposited onto the photoelectrode surface and subsequently activated during PEC operation to form new catalysts [250]. Modifying precursor materials and engineering of active site regeneration, surface defect healing, and reaction condition adaptation help maintain catalyst activity and prevent degradation during PEC operations [156]. For PEC water splitting, a variety of in situ produced catalysts were employed, notably metal‐hydroxides, oxyhydroxide, and oxides in strong alkaline electrolytes [251]. Normally, *in*‐situ catalyst production occurs when catalytic materials are created or activated directly on the surface of the photoelectrode during the PEC operation. In situ produced catalysts adapt quickly to the reaction environment, improving reaction kinetics and extending photoelectrode life by preventing catalyst deterioration. This strategy is especially helpful for PEC water‐splitting applications, where it's essential to maintain a high‐performance oxygen evolution catalyst to sustain PEC device performance [249].

**FIGURE 14:**
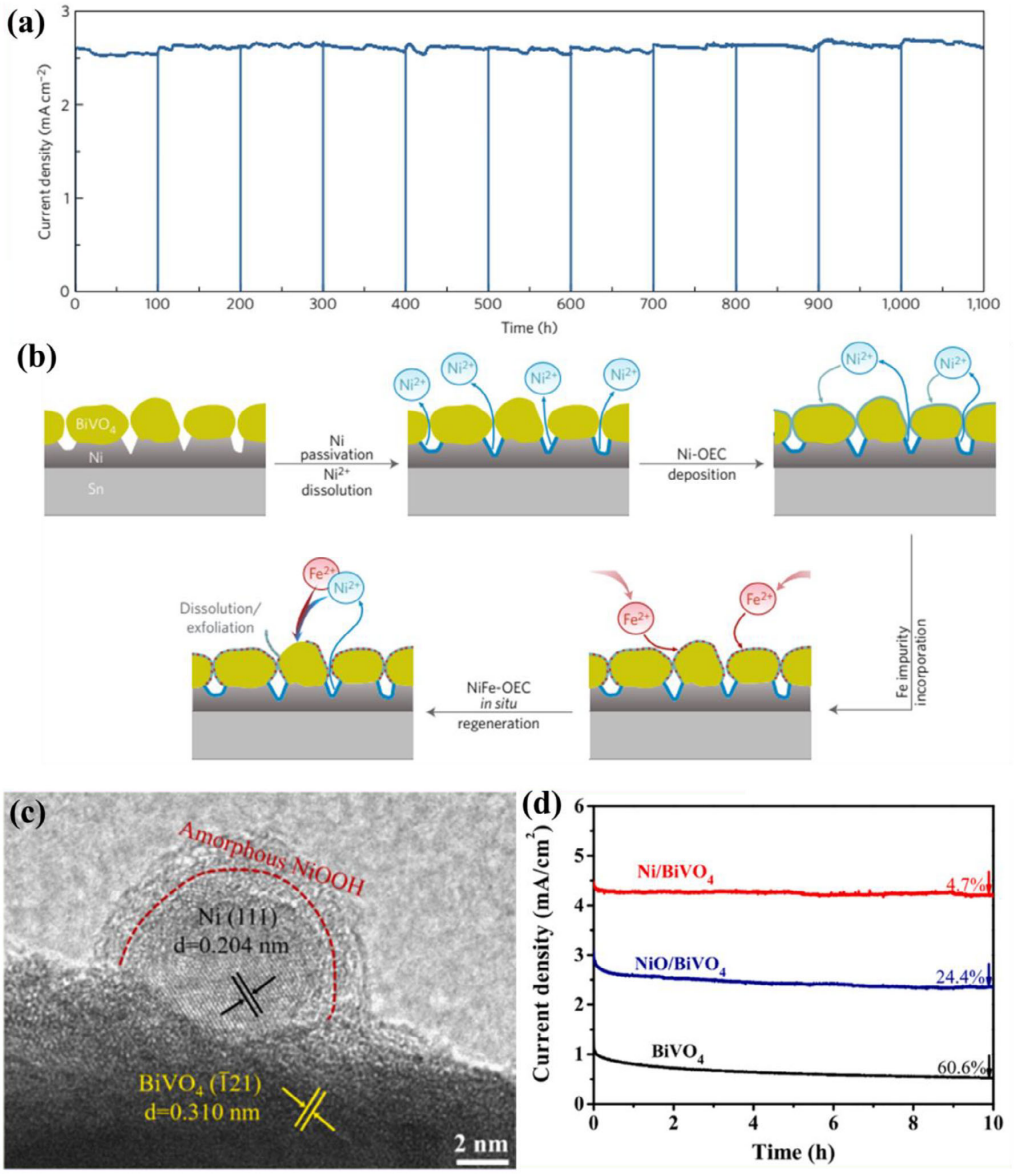
(a) Long‐term stability result for Sn/Sn/Mo:BiVO_4_/NiFe structured photoanode at 0.6 V_RHE_ in 1 M borate buffer electrolyte under AM 1.5G irradiation. (b) Schematic illustration of NiFe‐OEC self‐generation and *in*‐situ regeneration mechanism. Reproduced with permission [156]. Copyright 2016, Springer Nature. (c) HR‐TEM image for Ni/BiVO_4_ photoanode after long‐term stability test and (d) long‐term stability for various BiVO_4_‐based photoanodes at 1.23 V_RHE_ under AM 1.5G illumination.

Domen and co‐workers reported [156] a significantly stable photoanode for water oxidation through photocorrosion inhibition and *in*‐situ catalyst regeneration strategies. The Sn/Ni/Mo(0.3 at%):BiVO_4_/NiFe structured photoanode exhibited excellent photocurrent density over 4 mA/cm^2^ and an ABPE of 1.6% at 1.23 V_RHE_ in 0.2 M sodium sulfite in a 1 M potassium borate buffer (pH 9) under AM 1.5G irradiation. Particularly, this photoanode achieved outstanding long‐term stability over 1100 h without rapid photocurrent density degradation at 0.6 V_RHE,_ as shown in Figure 14a. In this regard, authors hypothesized *in*‐situ catalyst self‐generation and regeneration of OEC during PEC operation, as shown in Figure 14b. Detailed processes for in situ catalyst self‐generation were well discussed.

The OEC *in*‐situ regeneration strategy offers an opportunity to make more economically viable PEC devices. BiVO_4_ has been demonstrated to dissolve gradually in phosphate buffer electrolytes in the past [252]. While phosphate‐containing electrolytes could aid NiFe‐OEC catalyst regeneration, they are incompatible with Ni‐based catalysts as phosphate ions accelerate catalyst dissolution. The discoveries make it possible to create effective printable electrodes with inkjet printing, which lowers manufacturing costs. Qiu and co‐workers successfully demonstrated a significant boost in performance and long‐term stability in BiVO_4_‐based photoanode via *in*situ formation of Ni/NiOOH co‐catalyst during PEC operation [253]. The BiVO_4_/Ni structured photoanode exhibited a significantly improved photocurrent density of 4.41 mA/cm^2^ at 1.23 V_RHE_ (bare BiVO_4_ ∼1 mA/cm^2^) and high ABPE of 2% (bare BiVO_4_ ∼0.3%) under AM 1.5G illumination. Interestingly, the HR‐TEM image (see Figure 14c) clearly showed that an amorphous NiOOH layer formed on the Ni nanocrystal surface after long‐term stability test, thereby forming a core‐shell Ni@NiOOH co‐catalyst. This photoanode achieved significantly improved long‐term stability over 10 h (∼4.7% decrease as compared to the initial value) as shown in Figure 14d. On the other hand, the photocurrent densities for BiVO_4_ and BiVO_4_/NiO photoanode dramatically decreased up to 60.6 and 24.4%, respectively, after PEC operation over 10 h. Authors revealed the role of in situ formed Ni@NiOOH co‐catalyst on BiVO_4_ photoanode by charge migration, XPS characterizations, and photocurrent density simulation. In situ formed Ni@NiOOH co‐catalyst offers high electrical conductivity and high oxygen activity, leading to a significantly enhanced device performance and long‐term stability.

Practical PEC water‐splitting systems depend on developing robust and extremely effective photoelectrodes in neutral electrolytes [5, 203, 229]. Normally, strong acidic or alkaline electrolytes can be a good option in PEC water splitting systems due to their advantages in photocurrent density and reaction kinetics, even though these conditions may necessitate careful material selection to prevent photocorrosion, particularly in PEC applications aimed at optimizing the water splitting process. Compared to strong acidic or alkaline electrolytes, neutral electrolytes provide several benefits, such as excellent long‐term stability, material reliability, and sustainability for photoelectrodes [204]. However, high PEC activity and long‐term stability in neutral environments still need to be simultaneously achieved. For the duration of PEC water splitting, the electrochemical environments of the photoelectrode are significantly impacted. Specifically, the electrolyte pH significantly affects the photoelectrode's energy bands as well as the electrical properties of the electrolyte [254]. The electrolyte provides a reaction environment for water oxidation and H^+^ reduction, and constructs ion transport channels. Additionally, the electrolyte controls the characteristics of the interface between solids and liquids [14, 255]. Through the use of these tactics, scientists are consistently enhancing the performance and longevity of photoelectrodes in neutral electrolytes, therefore advancing the realization of feasible and enduring PEC water‐splitting devices for H_2_ production. As a result, precisely controlling the electrolyte's characteristics may be a useful strategy for raising the PEC performance [256]. When it comes to PEC operation, the neutral electrolyte has several benefits over strongly acidic or alkaline electrolytes. The stability and longevity of the photoelectrodes are significantly improved by neutral electrolytes owing to their less corrosive nature [204, 257]. It can minimize the photoelectrode dissolution and/or deterioration during PEC operation.

The neutral electrolyte can offer a stable PEC operation process; however, their device performance of a PEC device is relatively poor. One intriguing strategy for improving the long‐term stability of PEC water splitting is in situ catalyst synthesis during PEC operation. With this strategy, in situ catalytic layers are generated directly on the photoelectrode surfaces during PEC operation. By ensuring that the catalyst layer stays tightly bound to the photoelectrode and is well‐integrated, this adaptive mechanism lowers the possibility of deterioration and delamination. An adaptable strategy for PEC operation in neutral electrolytes can promote charge separation and transport in photoelectrodes. In particular, the in situ formation of a catalyst allows the catalytic surface to regenerate continuously, which compensates for any losses or degradation over time. These in situ generated catalysts frequently adjust to the local electrochemical circumstances, improving the overall efficiency of the reaction. Finally, neutral electrolytes can ensure more stable and efficient PEC operation. In the end, this strategy strengthens and extends the PEC system, enhancing its performance for continuous H_2_ production and helping to improve long‐term stability.

In a nutshell, electrolyte engineering has emerged as a key approach for enhancing both the stability and efficiency of PEC water‐splitting systems by carefully controlling the chemical environment at the semiconductor/electrolyte interface. Unlike protective passivation or interface engineering, which mainly focus on modifying the photoelectrode structure, electrolyte engineering targets the optimization of electrolyte composition, pH, ionic species, buffering capacity, and reaction intermediates to suppress photocorrosion while preserving efficient charge‐transfer kinetics.

Well‐designed electrolytes can limit semiconductor dissolution, stabilize surface chemical states, regulate interfacial reactions, and support catalyst regeneration during extended operation. Recent studies have shown that incorporating redox‐active ions, corrosion‐inhibiting additives, or saturated electrolyte species can significantly extend photoelectrode lifetime without compromising PEC performance. In addition, the electrolyte plays a crucial role in determining band‐edge positions, charge‐transfer behavior, catalyst stability, and overall reaction kinetics, making it a vital factor in the design of durable PEC systems.

This section discusses key electrolyte‐engineering strategies‐including optimization of electrolyte composition, the use of corrosion‐suppressing additives, in situ catalyst regeneration, and operation in near‐neutral electrolytes‐with emphasis on how these approaches improve both PEC performance and long‐term stability.

### 4.4 Deposition and Fabrication Strategies

**FIGURE 15:**
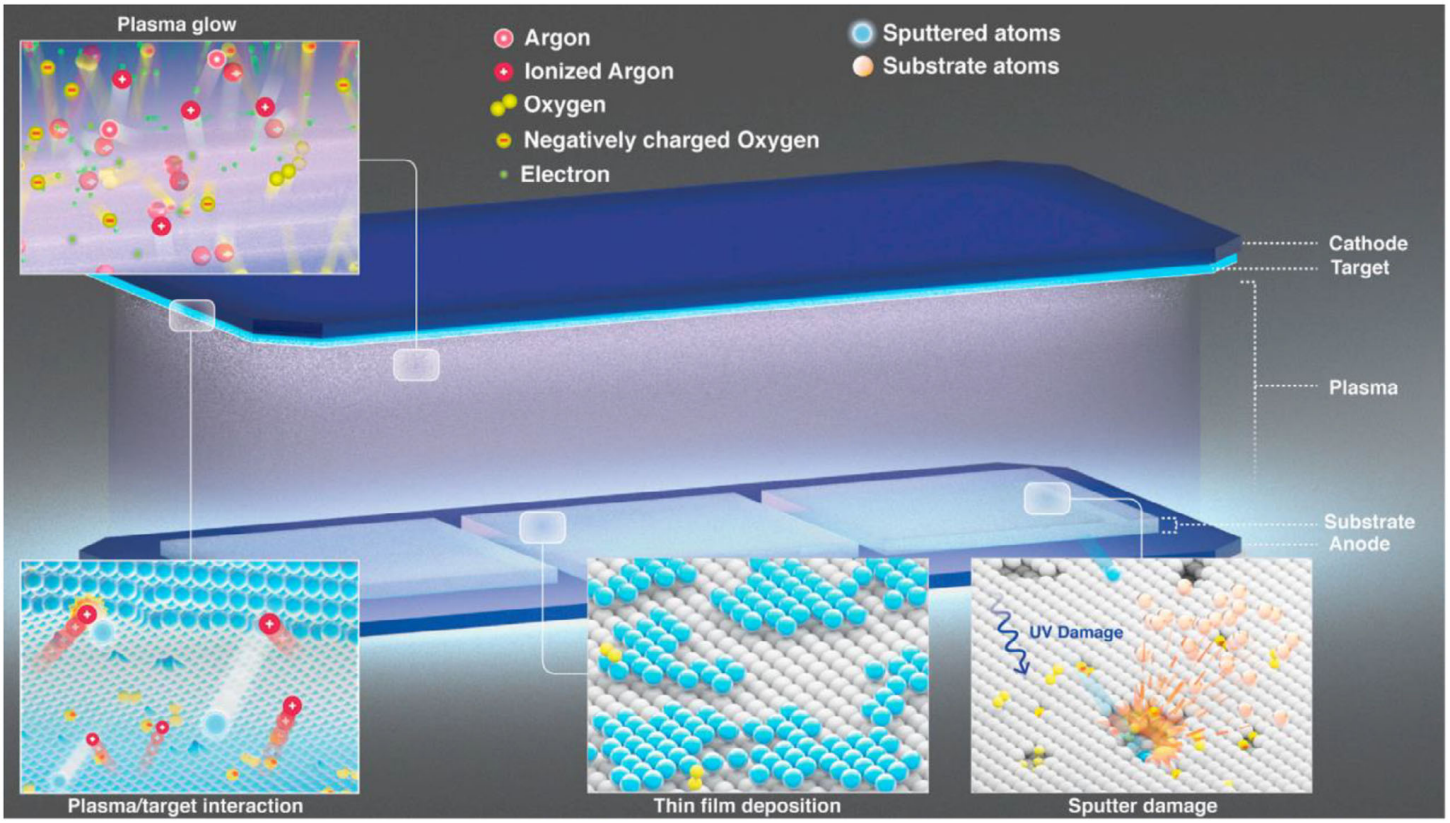
Illustration of the sputtering process and possible damage mechanism of the underlying layer. Reproduced with permission [267]. Copyright 2021, Elsevier.

As mentioned in the above sections, a PEC water splitting device with high performance and long‐term stability is essential to be fabricated with multilayered structures (i.e., normally, photoelectrode/protective layer/catalysts‐based structures). Normally, photoelectrodes in the PEC water splitting are susceptible to several forms of damage on the photoelectrode surface caused by several techniques [4, 8]. Numerous things, such as the materials utilized, the preparation under certain circumstances, and the deposition process itself, might result in the formation of abundant defects and related defect clusters on the photoelectrode surface [258]. Throughout the vacuum deposition process, the photoelectrodes experienced surface damage, various defects, and related defect clusters [259]. These imperfections tend to significantly scatter photogenerated carriers, trap charge carriers, and serve as sites for photogenerated electron and hole recombination at interfaces [260, 261]. Typically, chemically bath‐deposited CdS is employed as a protective and buffer layer on the metal‐chalcogenide‐based photocathodes (i.e., CZTSSe and Sb_2_(Se,S)_3_) to improve device performance and long‐term stability [120, 262]. Subsequently, ZnO or TiO_2_‐based compounds bi‐layered transparent conductive electrode were normally prepared by vacuum‐based techniques (i.e., sputtering‐based approach), which has low cost, excellent optoelectronic properties, and superior chemical and thermal stability [263]. Particularly, the plasma emission (i.e., Ar or oxygen ions), momentum transfer of high‐energy particles from plasma, and increased substrate temperature during the sputtering‐based deposition process resulted in significant damage (i.e., etching or thermal degradation) to the underlying sensitive photoelectrodes, as shown in Figure 15. This can adversely affect the optoelectronic properties of underlying charge transport, passivation, and protective layers. From previous reports, a locally formed damaged CdS layer was observed after the sputtering process [120]. In this regard, less‐ or no‐damage deposition approaches, such as vacuum‐ and solution‐based techniques, have been widely established [264, 265, 266, 267]. Related discussions, including ALD‐ and chemical vapor deposition (CVD)‐based approaches, will be presented.

As‐mentioned above, the chemical‐based approaches for preparing protective layers on photoelectrodes are used to improve durability, stability, and efficiency when producing catalysts, protective coatings, and photoelectrodes for PEC water‐splitting devices [8, 162]. Furthermore, chemical‐based approaches for protective materials or layers can also offer improved regulation of particle size, surface area, and catalytic activity. Several chemical‐based synthesis strategies can offer easy preparation while minimizing damage to the underlying photoelectrodes in PEC water‐splitting devices to improve the long‐term stability and performance. Some approaches, such as sol‐gel and hydrothermal techniques, can provide precise control over the compositional ratio, crystal structure (i.e., amorphous/crystalline or multi‐phase), and excellent optoelectronic characteristics of protective materials, guaranteeing demanding standards of PEC systems for the industrialization stage. The recently advanced PEC water splitting technologies' stability and device performances are contingent upon these procedures [14, 255]. Moreover, surface passivation using chemical ions or species during the preparation of a protective layer can prevent the abundant defect sites on the photoelectrode, which would lead to a high recombination rate at the interface and device degradation [160, 268]. Designing systems with minimal mechanical loads and a rate of photocorrosion is vital for preventing the deterioration of photoelectrodes in PEC water splitting. Furthermore, it is imperative when operating PEC water‐splitting equipment in environments that inhibit fouling or photocorrosion [41]. The advancement of solar‐to‐fuel conversion technologies depends on the development of a damage‐free deposition technique that enhances the long‐term stability of PEC photoelectrodes. These approaches can offer minimal mechanical and thermal stress for preparing protective materials on photoelectrodes at relatively low temperatures. The chemical approaches can offer significantly reduced damage to the underlying protective layers on the photoelectrode and the passivation role of defects and related defect clusters on the photoelectrode surface. However, some drawbacks, including non‐uniformity (i.e., roughness and thickness), abundant defects, slow deposition rate, material compatibility, solvent limitations, environmental concerns (i.e., toxic waste production), scalability, and industrial production, are still faced [269, 270].

ALD and CVD‐based approaches have been widely known as less or no damage vacuum‐based deposition techniques at relatively low temperature and energy in optoelectronic applications such as photodetectors, solar cells, and water splitting devices [172, 271]. ALD can offer a precisely adjusted compositional ratio, uniformity, and thickness through controlling the number of precursor cycles and pulse sequences [172, 191]. The precursor type, cycles, pulse rate, and substrate temperatures are critical factors to prepare high‐quality for uniform, and/or complex nanostructures protective layers on photoelectrodes [172]. In this regard, Grätzel and co‐workers [272] first demonstrated the meaningful results for ALD‐TiO_2_ protective layer on Si‐based photoelectrode with excellent long‐term stability over 30 days in acidic electrolyte through the post‐annealing process. This photoelectrode showed the photocurrent density of −22 mA/cm^2^ at 0 V_RHE_ and an onset potential of 0.51 V_RHE_. However, the post‐annealing process may not apply to the industrial stage.

On the other hand, the ALD process relies on the sequential exposure of two and/or three gaseous precursors, and these precursors adsorb onto the surface of the growing film and/or the substrate. Inert gas purging is used to separate the steps, which removes any remaining and unattached precursor molecules [273] Particularly, exceptional step coverage with uniformity using the ALD technique can be produced by the self‐limiting feature at relatively low deposition temperature, even though a 3D nanostructured photoelectrode [171, 172]. Normally, preparing a protective layer on 3D structured photoelectrodes faced some difficulties, such as (i) overcoming a short carrier diffusion length, (ii) encouraging carrier separation, and (iii) improving light collection [274]. These issues can be resolved with accurately regulated thickness and the material's composition. In this regard, ALD has been utilized to create a wide range of specialized nanostructures, including NRs and NWs, combined with nano‐trenches [275, 276]. Lewis and co‐workers [277] reported the ALD‐*a*‐TiO_2_ protective layer on a Si microwire array structured photoanode with excellent long‐term stability over 2200 h without rapid degradation in 1.0 M KOH under simulated 1 sun illumination. The same group reported the ALD‐NiO‐based protective layer on the Si photoanode with outstanding long‐term stability over 1200 h in 1.0 M KOH electrolyte [278]. Furthermore, they also reported the excellent long‐term stability over 1700 h for the Si‐based photoanode by introducing an ALD‐based CoO_x_/NiO_x_ protective layer at 1.63 V_RHE_ and photocurrent density of 28 mA/cm^2^ in 1.0 M KOH under illumination of 100 mW/cm^2^ [279]. Morante and co‐workers successfully introduced the ALD‐based TiO_2_ protective layer on ZnO‐based TCO/CdS/CZTSSe structured photocathode [280]. Authors investigated the effect of deposited temperatures (200‐300 °C) of the TiO_2_ protective layer on device performances for the CZTSSe‐based photocathode in 0.5 M H_2_SO_4_ at 0 V_RHE_ under 1 sun AM 1.5G illumination conditions. As a result, this photocathode exhibited the excellent photocurrent density of ‐37 mA/cm^2^ at 0 V_RHE_, HC‐STH of 7% and long‐term stability over 1 h in a highly strong acidic electrolyte. The same group investigated the degradation and regeneration mechanism of the ALD NiO protective layer on the Si‐based photoanode in an alkaline electrolyte [281]. Authors found that (i) the deposition temperature and thickness of ALD NiO protective layers crucially affected the device performances, long‐term stability of Si‐based photoanode, and (ii) deposition conditions of NiO protective layers were strongly related to their optoelectronic properties. Based on extensive characterizations for long‐term stability investigation with various NiO protective layers and operation conductions, authors proposed the degradation mechanism as followings; (i) formation of in situ protective layer (i.e., Ni(OH)_2_ and/or NiOOH) during PEC operation owing to interaction between NiO and alkaline electrolyte under anodic working condition and (ii) observation of second degradation under illumination owing to reduced incident light from *in*‐situ protective layer. Authors also suggested that the periodic scans from anodic to cathodic voltage could promote a partial recovery procedure for device performance and long‐term stability. As a result, this photoanode exhibited excellent long‐term stability over 1000 h compared to that of the initial photocurrent density at 1.7 V_RHE_ in 1 M KOH electrolyte. Similarly, Grätzel and co‐workers [107] introduced the ALD‐TiO_2_ as a protective layer on NW‐structured Cu_2_O‐based photocathode with significantly improved device performances and long‐term stability. Interestingly, the ALD‐TiO_2_ protective layer showed excellent coverage with a high aspect ratio (over 2 µm) and uniform thickness. Through introducing NW‐structured Cu_2_O/Ga_2_O_3_ buried *p‐n* junction as well as ALD‐TiO_2_ protective layer, this photocathode achieved the excellent long‐term stability over 100 h without rapid degradation, a large onset potential of 1 V_RHE_, and STH efficiency over 3% in weak alkaline electrolyte conditions. Several excellent reports for ALD‐based protective layers on photoelectrodes have been investigated [94, 195, 282, 283, 284, 285, 286, 287]. The process of ALD comprises the layer‐by‐layer application of protective layer deposition, including metal oxides, on complicated photoelectrode surfaces in a uniform and conformal manner, leading to significantly improved device performance and long‐term stability for PEC water splitting applications. However, a critical limitation of the ALD‐based approach is its slowness. Deposition rates are typically 100–300 nm/h and preparing micrometer‐thick protective layers is not the most effective. Manufacturing big batches of substrates or devices in a single process can make up for the slow growth rate. Another drawback is the presence of remnants of the precursors and carrier gas on the photoelectrode surfaces and the easy formation of unwanted interfacial and/or interlayer, playing a role as recombination centers between the photoelectrode and electrolyte. Moreover, the plasma‐induced damage is a constraint that can significantly impact specific applications during ALD operations. The deposition surface of the ALD cycle is exposed to a wide range of reactive species from the plasma, and these species have the potential to cause undesirable surface reactions, such as oxidation and nitridation of the substrate's upper surface coatings [288, 289]. In this regard, the defects and related defect clusters for the protective layer on photoelectrodes might also be easily formed under plasma damage. Accelerated ions in the plasma sheath can assault the underlying materials or surface, displacing atoms in the surface area, breaking bonds, and accumulating charges on the protective layer [290]. Furthermore, prolonged plasma treatment might cause undesired stress, phase shifts, or crystallographic flaws, affecting the film's mechanical, electrical, and optical features.

On the other hand, the CVD technique is a versatile process used to deposit very thin layers or coatings by chemically reacting gaseous precursors (i.e., volatile inorganic, metal‐organic, or organometallic materials) on the heated substrates [291, 292]. CVD has some advantages, such as flexibility for deposition materials, high deposition rates, excellent coverage and homogeneity, precisely adjustable compositional ratios, and an easy doping process for the protective layer on photoelectrodes [291, 293, 294]. There are many reports that the CVD approach has been used to improve the photoelectrode's long‐term stability and performance [293, 295, 296]. In this regard, Zhang and co‐workers [297] studied the MoS_2_ protective layer prepared by the CVD approach on NW‐structured Si‐based photocathode for the first time. CVD‐based nanosheet MoS_2_ protective layer exhibited excellent coverage and uniformity on the Si photocathode, which generated a substantially greater photocurrent density of active sites. Additionally, this photocathode also showed the outstanding photocurrent density of ‐16.5 mA/cm^2^ at 0 V_RHE_, onset potential of 0.26 V_RHE_, and exceptional long‐term stability after 48 h without rapid degradation in 0.5 M H_2_SO_4_ (pH 0.5) electrolyte under light illumination. Unfortunately, the CVD‐based protective layer on the photoelectrode has been reported to show relatively poor long‐term stability compared to that using the ALD‐based technique [298, 299, 300, 301, 302]. Furthermore, there are some drawbacks like easy damage to the underlying layer owing to high deposition temperature, hazardous byproducts, poor large‐scalability, expensive precursor cost, and highly demanding facility, which are limitations to early industrialization [291, 303].

### 4.5 Design Principles

As summarized in Table 4, an ideal photoelectrode should combine a suitable E_g_ (1.6 to 2.2 eV), favorable band alignment, long carrier diffusion length, low defect density, and excellent intrinsic chemical stability. Simultaneous optimization of these parameters remains the primary challenge in PEC water splitting.

**TABLE 4:** Design principles for photoelectrode selection.

Parameters	Desired features	Remarks
E_g_	1.6–2.2 eV	Excellent light absorption under visible region and high photovoltage
Carrier diffusion length	100 to 1000 nm	Reduce bulk and interfacial recombination
Band alignment	Favorable band positions between electrolyte and HER/OER/catalysts	Excellent charge transfer
Defect density	Low concentrations	Suppress recombination and photocorrosion
Electrolyte window in stability	Wide Pourbaix stability	Long‐term operation

The fundamental design considerations required to develop high‐performance and long‐lasting PEC photoelectrodes. An optimal semiconductor bandgap in the range of 1.6–2.2 eV is crucial to balance effective solar light absorption with sufficient photovoltage generation. Proper band alignment with the HER and OER redox potentials, along with well‐matched interfacial energy levels, plays a vital role in facilitating efficient charge transfer and improving operational stability. Minimizing defect density is equally important, as it extends carrier lifetime and reduces recombination losses, thereby enhancing both efficiency and device reliability.

The introduction of a compact, pinhole‐free protective layer can substantially improve chemical stability, although it may introduce a minor efficiency loss due to added transport resistance. In addition, the use of highly active HER/OER catalysts promotes faster interfacial reaction kinetics, lowering overpotentials and boosting overall PEC performance while supporting long‐term stability. Well‐engineered semiconductor/electrolyte interfaces with minimal recombination losses further aid in efficient charge separation and collection, contributing to improved efficiency and durability. Lastly, selecting an electrolyte with a stable pH range is critical to preserve photoelectrode integrity and ensure sustained operation under PEC conditions. Overall, achieving practical PEC water‐splitting systems requires the simultaneous optimization of all these interconnected design parameters.

### 4.6 Strategies for Efficiency and Stability

As discussed throughout this review, chalcogenide‐based photoelectrodes, together with other semiconductor systems, have demonstrated remarkable progress in PEC water splitting, particularly in achieving higher photocurrent densities through bandgap tuning, defect control, and nanostructuring. However, translating these advances into practical systems remains challenging because efficiency and long‐term stability rarely improve together. In many cases, strategies that boost performance also accelerate degradation, leading to photocorrosion, surface oxidation, structural instability, catalyst deactivation, and increased charge recombination during operation [304, 305].

For PEC systems to be viable, high STH conversion efficiency must be sustained over extended periods. This makes it essential to move beyond isolated improvements and instead adopt integrated design strategies that simultaneously optimize light absorption, charge dynamics, surface reactions, and material durability [147, 305]. The following discussion outlines key approaches for improving both efficiency and stability, with an emphasis on how these strategies can be combined to enable robust and high‐performance PEC systems.

#### 4.6.1 Strategies for Improving Efficiency

Improving the efficiency of all photoelectrodes fundamentally requires better control over how light is absorbed and how photogenerated carriers are generated, separated, transported, and utilized at the surface. One of the most effective strategies is defect engineering. Carefully introduced shallow defects can enhance carrier concentration, but uncontrolled deep‐level defects often act as recombination centers. Managing this balance is therefore critical for improving carrier lifetime and transport [237].

Future efforts should increasingly focus on advanced band engineering approaches. Through doping, alloying, or compositional tuning, the bandgap can be adjusted to enhance visible‐light absorption while aligning the band edges more favorably with hydrogen and oxygen evolution potentials. This directly improves both light harvesting and reaction thermodynamics [306].

Heterojunction construction further strengthens performance by introducing internal electric fields that promote spatial separation of electrons and holes. This reduces recombination losses and improves charge extraction. In parallel, nanostructuring‐such as forming nanorods, nanosheets, or porous networks‐shortens carrier diffusion lengths and increases active surface area, allowing more efficient interaction with the electrolyte [160, 307].

Surface reaction kinetics often become the rate‐limiting step, which makes cocatalyst integration particularly important. Materials such as Pt, MoS_2_, NiFeOx, CoPi, and transition‐metal phosphides can significantly lower overpotentials and accelerate interfacial charge transfer. At the same time, interface engineering‐through passivation layers or optimized contact interfaces‐helps suppress recombination while maintaining efficient carrier extraction.

An emerging direction is crystal‐phase engineering, especially the use of amorphous‐crystalline hybrid structures. Crystalline domains provide efficient charge transport pathways, whereas amorphous regions offer abundant active sites and structural adaptability. Combining these features can lead to improved overall PEC performance [308].

Overall, it is increasingly clear that no single modification is sufficient. The most meaningful improvements in PEC efficiency are achieved when multiple strategies‐defect control, band engineering, heterostructures, nanostructuring, cocatalysts, and interface design‐are combined in a synergistic manner.

#### 4.6.2 Strategies for Improving Stability

While PEC often receives primary attention, long‐term stability is equally critical and remains one of the main obstacles for chalcogenide photoelectrodes. Under continuous illumination and applied bias, these materials frequently suffer from photocorrosion, oxidation, elemental loss, catalyst detachment, and structural degradation. As a result, even high‐performing systems can degrade rapidly, limiting their practical applicability [43].

Among the various approaches, surface passivation has proven particularly effective. Ultrathin oxide layers such as TiO_2_ or Al_2_O_3_, typically deposited by atomic layer deposition, can act as protective barriers that prevent direct contact with the electrolyte while still allowing charge transport. This significantly suppresses photocorrosion without sacrificing performance [309].

In addition, protective coatings and corrosion‐resistant cocatalysts help stabilize the electrode surface while maintaining catalytic activity. Interface engineering also plays a key role by improving adhesion between layers and reducing the likelihood of delamination during operation. Electrolyte engineering is another often underappreciated factor. Adjusting electrolyte composition, pH, ionic strength, and introducing redox mediators can significantly influence both reaction kinetics and degradation pathways. Meanwhile, compositional engineering‐through alloying or multinary chalcogenide design‐can enhance intrinsic chemical stability and resistance to photocorrosion [13, 310]. Hybrid amorphous‐crystalline architectures are particularly promising for stability as well. These structures combine the robust charge transport of crystalline phases with the defect tolerance and flexibility of amorphous regions, enabling them to better withstand structural and chemical stresses during operation.

Looking ahead, deeper insight into degradation mechanisms will be essential. Advanced operando characterization techniques, combined with computational modeling and machine‐learning‐assisted materials discovery, are expected to accelerate the development of more stable systems by enabling real‐time tracking and prediction of failure pathways [311, 312].

## 5 Fundamental Characterization Methods

**FIGURE 16:**
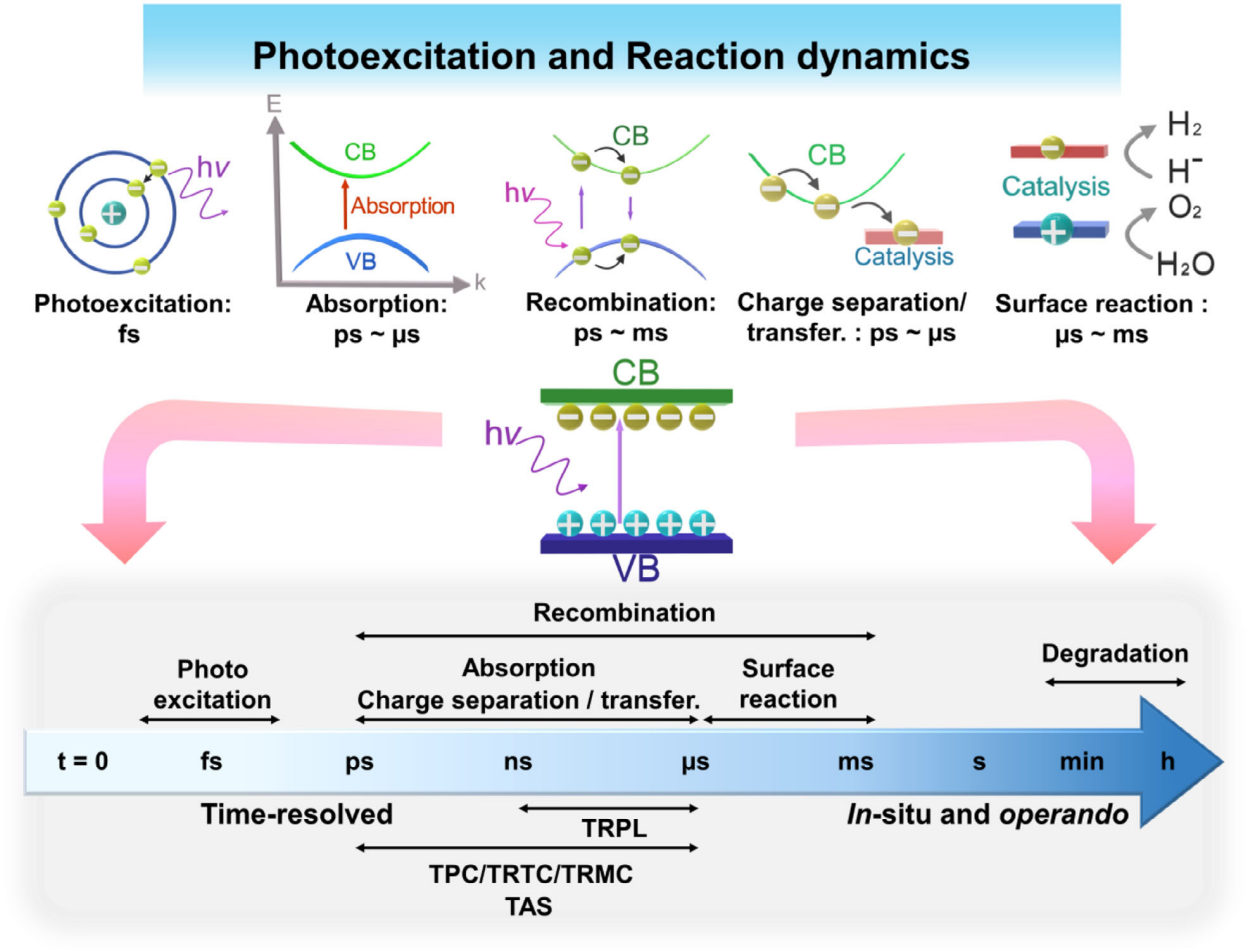
Schematic illustrating various advanced characterization tools for device performances and long‐term stability in PEC water splitting.

Recently, fundamental PEC device characterization tools have been developed to reveal material features and improve device performance. These tools provide various properties and detailed operation mechanism information, as provided in Figure 16. Characterization tools are broadly classified into two categories: (i) photoexcitation and reaction dynamics (i.e., absorption, recombination, charge separation and transfer, and surface reaction) and (ii) PEC reaction (i.e., performance degradation). The choice depends on the materials and system, under the required spatial and temporal resolutions. In this section, the simple principles, meaningful and recent advanced characterization tools, and representative examples for PEC devices will be presented.

### 5.1 Reaction Dynamics Analysis Methods

#### 5.1.1 Transient Absorption Spectroscopy (TAS)

**FIGURE 17:**
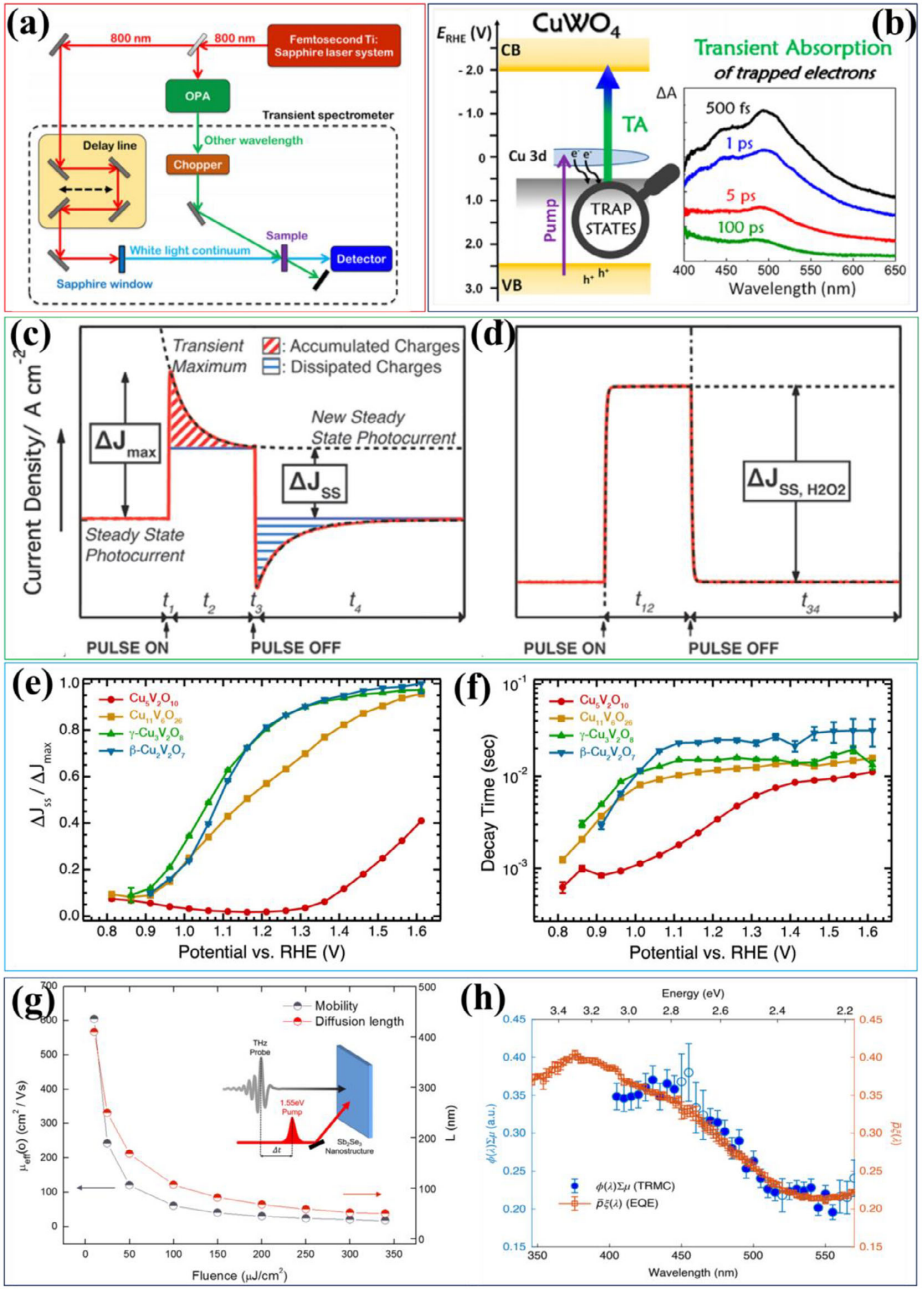
(a) Scheme for the working principle of TAS equipment. Reproduced with permission [315]. Copyright 2023, Royal Chemical Society. (b) Scheme for ultrafast relaxation and recombination pathways of electrons photopromoted in CuWO_4_ upon 40 nm excitation using the TAS tool and femtosecond TAS measured at different times after photoexcitation of CuWO_4_ at 400 nm under vacuum. Reproduced with permission [327]. Copyright 2021, American Chemical Society. Schemes of TPC tools were measured under (c) 1 M NaOH and (d) 1 M NaOH + 0.5 M H_2_O_2_ conditions. Reproduced with permission [319]. Copyright 2012, American Chemical Society. (e) The ratio between the steady state photocurrent density and photocurrent density as a function of applied potentials and (f) time constants obtained from the fitting of the transient photocurrent response of each photoanode. Reproduced with permission [320]. Copyright 2017, Cambridge University Press. (g) Diffusion length and effective mobility of Sb_2_Se_3_ nanostructured photocathode as a function of fluences calculated from the photoconductivity and time‐constant values. Reproduced with permission [325]. Copyright 2018, American Chemical Society. (h) Comparison of TRMC and photoelectrochemical EQE analysis. Reproduced with permission [326]. Copyright 2021, Nature Publishing Group.

The TAS technique can analyze the photogenerated charge concentration by monitoring the change in photoinduced absorption [313]. Identifying the pump pulse‐excited charge carriers on the sample is possible by tracking the probe signals that cause the photogenerated excitation to decay at various delay intervals. This information can help to deeply understand the recombination rate, trapping, and reaction of photogenerated electrons and holes [160]. Durrant and co‐workers earlier reported the recombination and carrier trapping mechanism concerning various Fe_2_O_3_‐based photoanodes [313]. In detail, they revealed that the water oxidation kinetics of Fe_2_O_3_‐based photoanodes strongly depend on the electron density from the electron‐hole recombination rate. The same group also studied the charge carrier trapping, recombination, and interfacial transfer for Fe_2_O_3_‐based photoanodes under external bias [148]. For further detailed reaction dynamics investigation, a femtosecond pump pulse has been introduced in the TAS equipment, and the scheme of the principle of the femtosecond TAS system is provided in Figure 17a. In this regard, Kennis and co‐workers have revealed the carrier dynamics, such as relaxation and trapping rates for electrons and holes in the BiVO_4_ photoanode using femtosecond TAS analysis [314]. Interestingly, authors found approximately 40 *p*s relaxation time and 2.5 ns deeper trapping time for photogenerated electrons and 5 *p*s trapped time for photogenerated holes in the BiVO_4_‐based photoanode. Recently, Selli and co‐workers revealed that the poor PEC performance of CuWO_4_ photoanode is strongly dependent on very fast recombination, as shown in Figure 17b. They suggested that the charge carriers are trapped in the Cu 3*d* orbital or lower energy mid‐gap states under the pump pulse in the CuWO_4_ photoanode. An excellent review covering the operating principle, fundamentals, and applications of femtosecond TAS analysis regarding the dynamics of photogenerated carriers in various materials has been reported [315].

#### 5.1.2 Transient Photocurrent/Photovoltage Spectroscopy (TPC/TPV)

TPC is well‐known as a useful analysis tool for studying the charge carrier dynamics and trapping of charge carriers in solar cells [316] and PEC water splitting systems [317], by monitoring the photocurrent responses in the samples [160, 318]. The decay mechanisms for photoelectrodes can contribute to the understanding of recombination and accumulation of charge carriers by fitting the transient decays and extracting time constants. Figure 17c shows the typical TPC response of a Fe_2_O_3_ photoanode with and without a pulse. Here, Δ*J_max_* is the transient maximum photocurrent density, and Δ*J_ss_* is the steady‐state photocurrent density under applied potential. When a pulse is incident on a photoelectrode, the photocurrent density exhibits a sharp anodic peak, which then eventually drops to Δ*J_ss_*. The cathodic current peak is observed without a pulse and then returns to the level of Δ*J_ss_*. As depicted in Figure 17d, the photoelectrode with a hole scavenger showed no transient decay during light‐pulse operation. The detailed calculation process for the distance at which the photogenerated hole originates inside the photoelectrode can be described by Formal and co‐workers [319]. Hayssener and co‐workers revealed TPC phenomena and two reaction pathways in a TiO_2_ photoanode by combining experiments and numerical analysis: such as (1) rapid charging of surface states and subsequent formation of acidic titanol group at the TiO_2_/H_2_O_2_ interface and (2) transfer process of H^+^ ions at the TiO_2_/Nafion interface in a TiO_2_ photoanode Sharp and co‐workers reported insights into the energy conversion and loss mechanism of copper vanadate compound‐based photoanodes by TPC analysis as shown in Figure 17e,f [320]. Quantitative TPC analysis revealed that the degradation of surface catalytic activity was strongly dependent on the Cu:V compositional ratio. Recently, TPC/TPV analysis for various photoelectrodes has been widely reported. These papers suggested that TPC/TPV analysis is very useful for studying the kinetics information of photogenerated charge separation, such as drift and diffusion processes on the nanosecond time scale. TPC/TPV analysis methods have merits for non‐contact and lossless detection, which can detect direct kinetic information like the separation direction, separation efficiency, and charge lifetime of photogenerated charges. As a result, the mechanism of charge recombination and guidance for improving the high efficiency of charge separation for PEC devices can be achieved.

#### 5.1.3 Time‐Resolved Terahertz/Microwave Conductivity (TRTC/TRMC)

TRMC measures the time‐dependent photoconductivity to investigate charge carrier dynamics and trap states for photoelectrodes under low‐frequency conditions [160]. Initially, Abdi and co‐workers demonstrated the origin of slow carrier transport (i.e., carrier lifetime ∼40 ns) in the BiVO_4_ photoanode using TRMC analysis [321]. Authors suggested that carrier mobility for BiVO_4_ with a W doping strategy, which introduces intermediate‐depth donor defects as carrier traps, is significantly decreased. In contrast, carrier density is increased, resulting in an improvement in the overall photoresponse. Furthermore, authors suggested that the significantly decreased electron and hole mobilities of the W‐doped BiVO_4_ photoanode were strongly attributed to charge carrier localization, as revealed by TRMC analysis [322]. The same group demonstrated the improved charge transfer characteristics and related mechanisms for hydrogen‐treated BiVO_4_ photoanode by combining TRMC and theoretical calculations [323]. Titova and co‐workers reported the ultrafast carrier dynamics, such as free carriers, trapped carriers, and low‐frequency lattice vibrations, in the BiVO_4_ photoanode using TRTC analysis and first‐principles photon spectrum calculations [324]. Authors found that (1) photoexcited holes form bound polaron states by introducing lattice distortion and (2) changing the lattice's symmetry and suppressing absorption at 1.92 THz by the A_g_ phonon mode. On the other hand, Moon and co‐workers demonstrated the photogenerated charge and carrier dynamics in the Sb_2_Se_3_ photocathode by TRTC analysis, as shown in Figure 17g [325]. The effective mobilities and diffusion lengths of photoexcited carriers in Sb_2_Se_3_ dramatically decreased with increasing fluence, which implies a lower trap density than that of Cu_2_O. Recently, Rothschild and co‐workers demonstrated the extraction of the mobile charge carrier photogeneration spectrum of metal‐oxide photoanodes with different illumination conditions and applied potentials [326]. Authors revealed that mobile charge carrier generation increases with increasing energy of the photoelectrode's absorption spectrum. Figure 17h shows the comparison of TRMC and EQE analysis for Sn‐doped Fe_2_O_3_ photoanode, and they are well‐matched under different wavelengths. It is suggested that TRMC analysis is a comparable method for probing the photogeneration yield spectrum, and it is not limited to ultrathin films.

### 5.2 *In*‐Situ Analysis Methods

#### 5.2.1 Raman Spectroscopy

**FIGURE 18:**
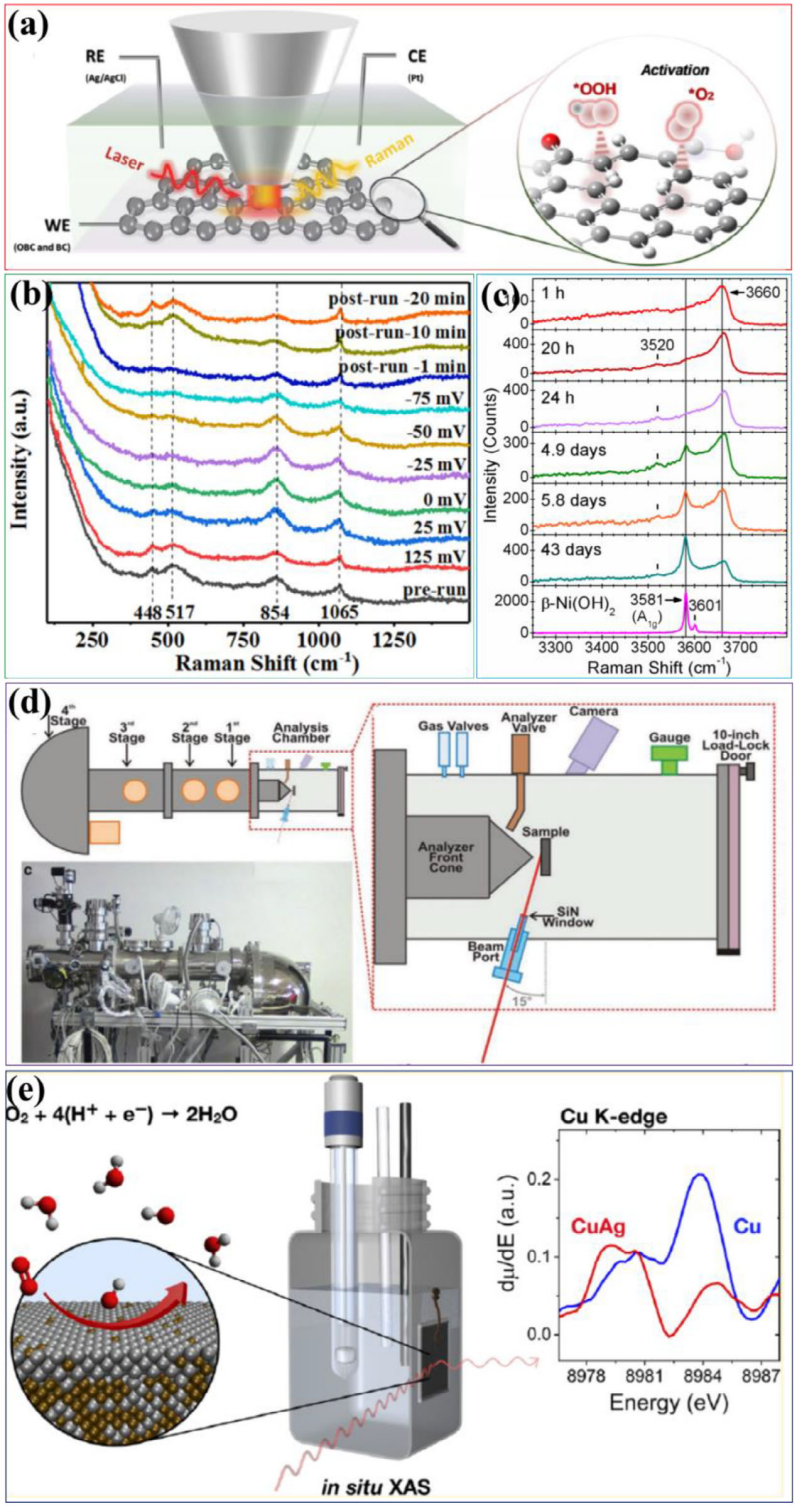
(a) Scheme of the *Operando* Raman analysis principle. Reproduced with permission [337]. Copyright 2022, Elsevier. (b) *in*‐situ Raman spectra of the AC‐Ni/NF catalyst with different potentials in 1 M KOH electrolyte. Reproduced with permission [338]. Copyright 2021, Elsevier. (c) The O‐H stretching region of the *in*‐situ Raman Spectra of β‐Ni(OH)_2_ with different aging days. Reproduced with permission [339]. Copyright 2014, University of Ottawa. (d) Scheme of XPS analysis equipment. Reproduced with permission [340]. Copyright 2015, Nature Publishing. (e) Scheme of XAS analysis equipment. Reproduced with permission [341]. Copyright 2020, American Chemical Society.

Raman spectroscopy is a classical technique to investigate molecular and/or crystal structures and to probe vibrational, rotational, and low‐frequency modes of materials using the vibrational spectroscopy principle [328]. Raman spectroscopy can detect the vibrational energy levels to provide information on the molecular composition and structure of the sample. Figure 18a shows the scheme of the Raman analysis principle. A monochromatic electromagnetic radiation beam (i.e., laser) interacts with molecular vibrations of the sample, resulting in inelastically scattered photons with different energy levels. The electron moves from the ground state to a higher virtual energy state when the laser is incident on the sample. Then the excited electrons fall to a lower vibrational state, and scattering spectra with a specific wavelength are released. Recently, the *in*‐situ Raman spectroscopy analysis tool has developed into a very useful technique to provide effective real‐time visualization and monitoring of the overall electrochemical reaction [311, 329, 330, 331]. The vibrational band associated with the presence of bonds, or a specific compound (molecules), can provide information on electrocatalytic performance. Ouyang and co‐workers reported *in*‐situ Raman analysis of the AC‐Ni/NF catalyst with different potentials in 1 M KOH electrolyte, as shown in Figure 18b. This result suggested that the Ni/Ni(OH)_2_ heterostructure is partially persistent and partially transformed to Ni metal and playing a critical role in the highly efficient HER catalysis on the AC‐Ni/NF catalyst. Hall and co‐workers reported *in*‐situ Raman analysis of *β*‐Ni(OH)_2_ electrocatalysis with different aging days shown in Figure 18c. Authors demonstrated the O‐H stretching modes for *β*‐Ni(OH)_2_ electrocatalysis with different aging days and revealed the phase transformation from *α‐* to *β*‐Ni(OH)_2_ structure. Reviews regarding recent advancements in Raman spectroscopy techniques, such as surface‐enhanced Raman spectroscopy, tip‐enhanced Raman, resonance Raman, stimulated Raman, and transmission Raman of electrochemical devices and in situ Raman analysis have been reported [328, 332].

#### 5.2.2 X‐Ray Spectroscopy (XPS)

XPS is one of the well‐known and classic analysis methods used to investigate the compositional ratio and chemical states of samples. It is very useful for studying the long‐term stability of PEC performance. Figure 18d shows the scheme of the XPS analysis equipment. When X‐rays are incident on the sample, the emission of electrons occurs via the photoelectric effect. The detector measures the kinetic energy of photoelectrons from their respective orbits (i.e., binding energy). Each core electron shows unique characteristics depending on the elements, oxidation state, and chemical environment in the sample. An excellent review regarding the investigation of material stability and interface dynamics in electrocatalysis and PEC applications has been reported [332].

#### 5.2.3 X‐Ray Absorption Spectroscopy (XAS)

XAS can provide insight and detailed element‐specific information on the electronic and geometric structures of catalytically active materials. Two meaningful pieces of information can be investigated from the XAS spectra for the samples. First, the X‐ray absorption near‐edge structure (XANES, ∼50 eV) provides element‐specific information about the electronic structure, density of unoccupied states, and bonding geometry of the absorbing atom [333]. Second, extended X‐ray absorption fine structure (EXAFS, 1000–1500 eV) reveals the local structure, including the identity, bond distances, and coordination number around the absorbing atom [333]. In this regard, the XAS tool can investigate structural information on amorphous phases, small solution species, and nanocrystals consisting of electrocatalysts and PEC devices. Jaramillo and co‐workers reported the role of Cu and Ag bimetallic catalysts for ORR using *in*‐situ XAS analysis and density functional theory investigation, shown in Figure 18e. This study demonstrates that improved ORR activity of the catalyst is caused by its direct electronic structure, rather than a geometric effect through, as revealed through *in*‐situ XAS Cu K‐edge XANES analysis. Excellent reviews on in situ and *Operando* electrocatalyst characterization using the XAS tool have been reported [333, 334, 335, 336]. They suggested that the XAS analysis tool can provide (1) qualitative and semi‐qualitative analysis from XANES spectra, (2) qualitative analysis from EXAFS data, (3) geometric structure descriptors like coordination numbers, interatomic distances, and disorder factors, (4) structure descriptors from XANES, (5) atomistic simulations for interpretation of EXAFS spectra, and (6) machine‐learning‐based EXAFS analysis.

#### 5.2.4 Infrared (IR) Spectroscopy

**FIGURE 19:**
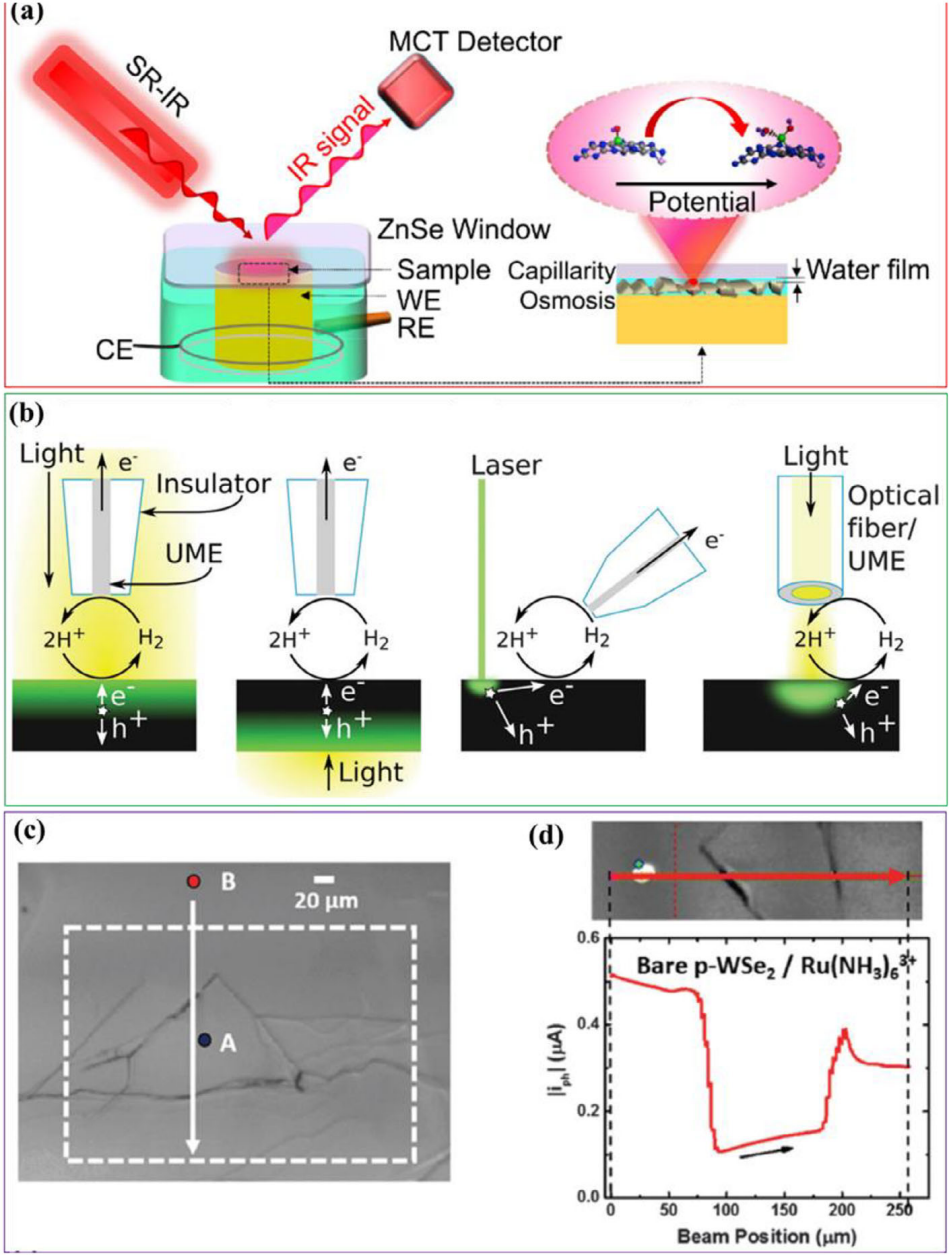
(a) Scheme of *in*‐situ IR equipment for PEC water splitting device. Reproduced with permission [343]. Copyright 2020, Elsevier. (b) Scheme of side‐views of several SECM systems. Reproduced with permission [19]. Copyright 2015 Royal Chemical Society. (c) Optical image and (d) photocurrent density measured as a function of beam position during a line scan as indicated by the white arrow in (c). Reproduced with permission [350]. Copyright 2016, Royal Society of Chemistry (RSC).

IR spectroscopy is widely used as an analysis method for the qualitative identification of samples and their structure [332, 342]. It is utilized when molecules absorb specific frequencies near the IR region. An IR spectroscopy tool can aid in understanding the reaction mechanism of the catalytic reaction and the presence of reaction intermediates in heterogeneous catalysis, as shown in Figure 19a [160, 343]. In particular, the in situ IR analysis techniques can provide (i) monitoring of real‐time PEC reaction processes and identification of reaction intermediates and (ii) adsorption and desorption processes at the photocatalyst surface [344, 345, 346]. Despite the merits mentioned above, conducting IR spectroscopy analysis under PEC operating conditions is a big challenge owing to strong IR absorption from water molecules. Interestingly, Hamann and co‐workers reported the existence of an intermediate at the surface of the Fe_2_O_3_ photoanode during PEC device working using in situ attenuated total reflectance IR spectroscopy analysis [347]. This study presented the first evidence of an H_2_O oxidation intermediate on photoelectrodes under PEC device working. Recently, Liu and co‐workers reviewed the insight into the dynamic evolution of liquid‐solid PEC interfaces for hydrogen/oxygen‐based intermediates to accelerate the screening process of catalysts using *Operando* IR spectroscopy analysis [343]. It covers the system‐level strategies of *operando* IR measurement for photo‐electrolytic liquid‐solid interfaces, including the light sources, optical pathways, and reaction cell design. Advanced IR‐related analysis techniques for electrocatalysis and PEC devices have also been reviewed [332].

#### 5.2.5 Scanning Probe Microscopy (SPM)

The scanning probe microscopy (SPM) techniques are used to visualize and image charge distribution at the surface or interfaces of catalysts or PEC devices to comprehend water‐splitting reactions more thoroughly. Traditionally, SPM is equipped with a sharp tip, and it can directly measure electrostatic force and current density between the probe and sample [348]. In this regard, the SPM technique can measure gaseous, vacuum, and liquid environments, and it is a useful analysis method to investigate the separation and distribution of photoexcited charges and surface actives of each sample. There are several SPM techniques, such as scanning photocurrent microscopy (SPCM), scanning electrochemical microscopy (SECM), Kelvin probe microscopy, and electrochemical atomic force microscopy. Here, we focus on the SECM technique, and the excellent reviews covering basic principles, classification, and detailed analysis for various SPM techniques in electrocatalysts and PEC devices have been reported [17, 18, 19, 160, 249]. Typically, an ultramicroelectrode (UME) is used as a scanning probe in SECM equipment, and it is a metallic disk or ring [19]. Figure 19b shows the scheme of side‐views of several SECM systems using (1) a conventional UME with a light source, (2) back illumination with a thin photoelectrode, (3) a focused laser beam, and (4) an optical fiber core UME with a light source. Generally, the SECM operation mode is substrate generation/tip collection. In this mode, H_2_ is generated from the reduction of protons at the photocathode's surface, while the UME tip oxidizes the H_2_ back to H^+^. H_2_O is oxidized at the photoanode, while O_2_ is reduced at the UME. In this regard, SECM can allow for mapping out and quantifying differences in H_2_ or O_2_ production across each surface in a photoelectrode. The in situ SECM technique helps to investigate spatially resolved information on the electrochemical reaction at the surface of the photoelectrode in the PEC device system. Talin and co‐workers successfully demonstrated the photocurrent density mapping under positive potential at the Si‐based photocathode and under negative potential at the UME tip [349]. Interestingly, these SECM mappings suggested that H_2_ evolution occurred at the collector surface as well as the surrounding SiO_2_ region. Lewis and co‐workers reported the differences in the charge collection performances on various macroscopic traces [350]. Figure 19c shows the optical image, and Figure 19d shows the photocurrent density measured as a function of beam position during a line scan as indicated by the white arrow in Figure 19c. Authors investigated that the SECM mapping for measured photocurrent density was strongly dependent on the terraces of WSe_2_. The measured photocurrent and PEC performance of the WSe_2_‐based photocathode with different terraces may be related to the non‐uniform distribution of surface and bulk defect states among terraces (particularly, chalcogen vacancies). Recently, nanoelectrode‐based SPM techniques have been developed to deeply investigate the surface electrochemical potential under PEC operation conditions and track electrochemical current density [351, 352]. These techniques employ a conical conductive tip that records the electrochemical current of redox reaction products from the catalyst surface to the probe, and their 100 nm resolution enables detailed catalytic reactivity with local surface morphology at the nanometer scale.

## 6 Computing‐Based Strategies

**FIGURE 20:**
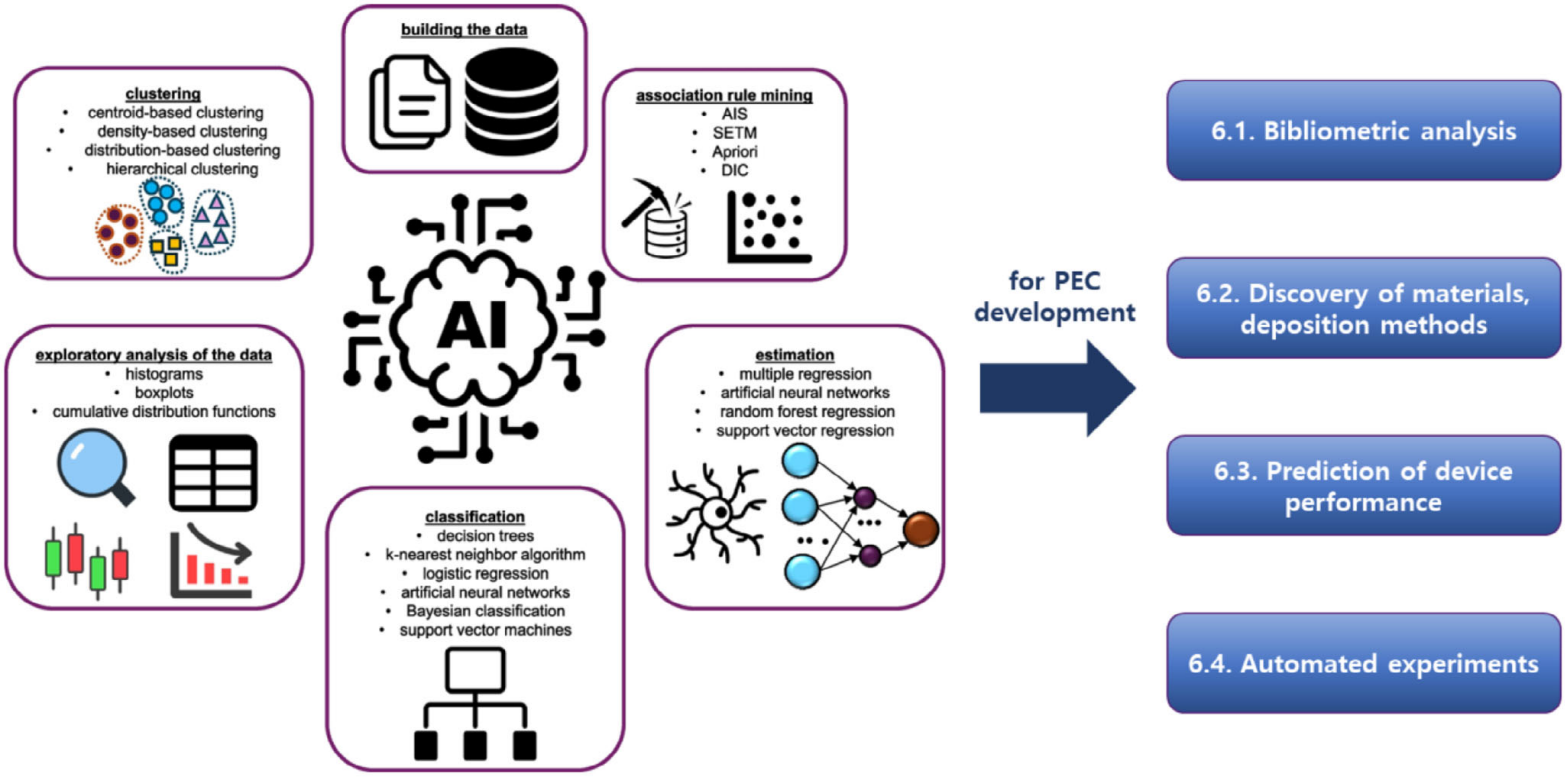
The conventional machine learning tasks and their popular algorithms, which can enable the computing‐based strategies introduced in Sections 6.1–6.4. Reproduced with permission [353]. Copyright 2024, Springer.

The computing‐based approaches, including bibliometric analysis, AI, DL, and ML methods, have been recently developed and allowed significant advancements in knowledge and unanticipated directions for the diverse research fields, including PEC water splitting [21, 22, 23, 26]. Even though not all of these approaches have been explored for PEC water splitting, there has been significant progress in the utilization of computing‐based approaches of various models across a range of scientific disciplines, suggesting their strong potential applicability to PEC water splitting. In particular, the data (i.e., collected literature and experimental results)‐driven approach can offer high potential to save the cost and experimental cycle times in the conventional trial‐and‐error research processes and can accelerate the materials discovery for photoelectrodes and/or catalysts, identifying critical factors and prediction for device performance and long‐term stability in PEC devices [312]. Typically, the ML approach is known as a subfield of AI, and it commonly aims to learn from past data using statistics and some algorithms. These algorithms include building the data, association rule mining, estimation, classification, exploratory analysis of the data, and clustering, as shown in Figure 20. The detailed information for ML, DL, and/or AI models has been excellently reviewed, and thus, we don`t mention them [21, 31]. In this section, we will review the computing‐based strategies, such as identifying critical factors by bibliometric analysis (Section 6.1), discovering new materials and deposition methods (Section 6.2), predictions for device performances and long‐term stability (Section 6.3), and automated experiments for improving performances (Section 6.4), respectively.

### 6.1 Bibliometric Analysis for Identifying Critical Factors

**FIGURE 21:**
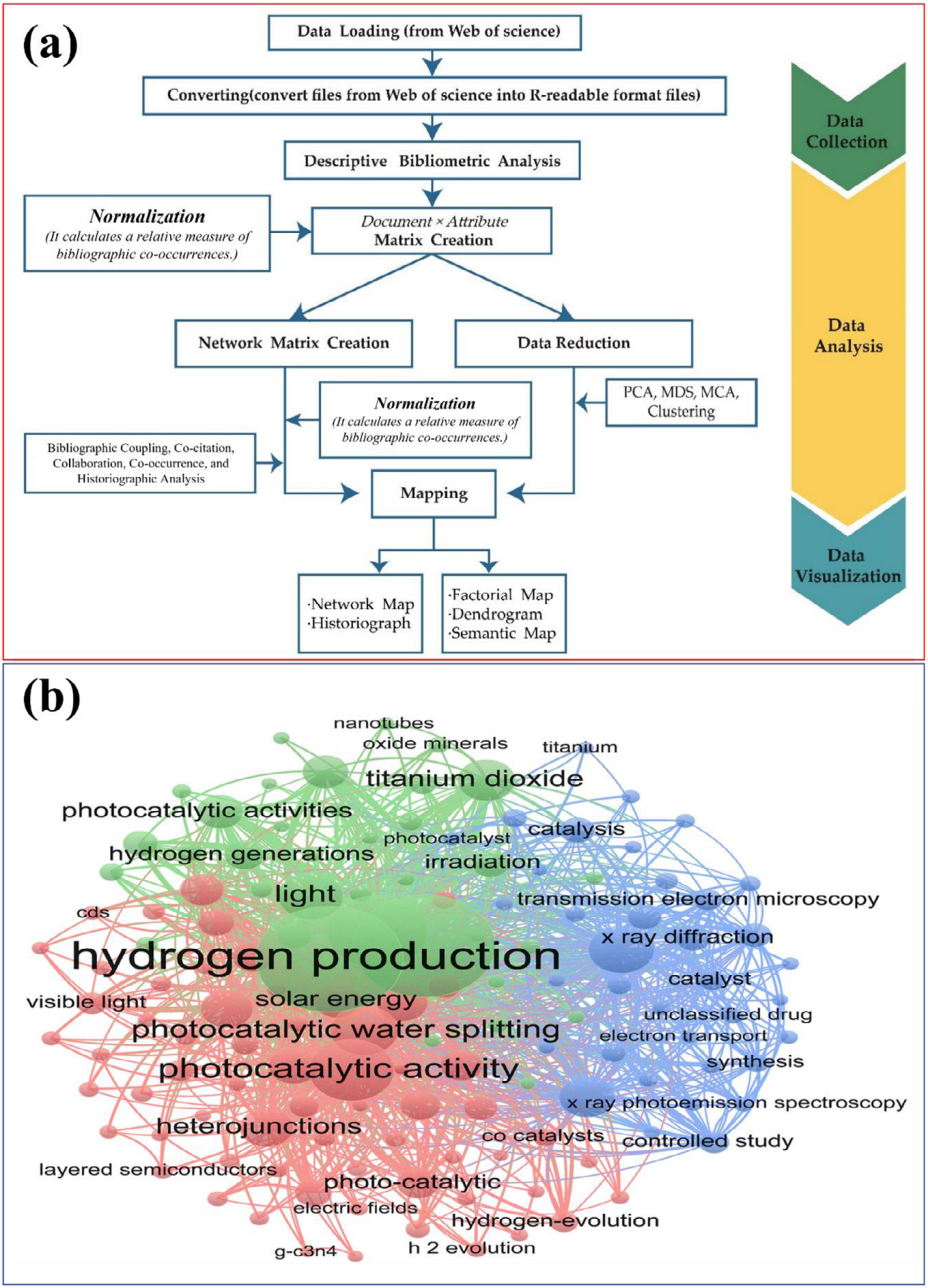
(a) Research flows for bibliometric analysis regarding PEC water splitting. Reproduced with permission [355]. Copyright 2024, Springer. (b) Co‐occurrence network with all keywords obtained by using the VOSviewer software. Reproduced with permission [232, 356]. Copyright 2025, Royal Society of Chemistry (RSC).

Bibliometric analysis has emerged as a useful characterization tool for identifying the critical factors in various material synthesis, property characterizations, and device performances. It uses a statistical algorithm to analyze many published research papers in specific fields to find developments and useful information for understanding the intellectual structure, impact, and collaborative networks. Additionally, it can also offer research patterns, trends, and impacts by exploring and analyzing large volumes of scientific data. Normally, the bibliometric analysis involves two main approaches: (i) device performance‐related analysis, such as publication, citation, author, co‐authors, nation, publication source, idea, technique, and important research points, (ii) science mapping; relationship and mapping for citation analysis, co‐citation analysis, bibliographic coupling, co‐word analysis, and co‐authorship analysis. In the case of science mapping, it easily identifies critical factors, influential works, emerging themes, research gaps, and the overall landscape of a new research area. Figure 21a shows the example for bibliometric analysis and mapping workflow, including study design, data collection, data analysis, data visualization, and interpretation process.

Yuliarto and co‐workers have developed the first meaningful bibliometric review study using more than 2900 articles on PEC devices in the Scopus database from diverse relevant journals [354]. Authors found various parameters, including annual advancements, keyword co‐occurrence, global participation, finding sponsors, impactful articles, and recent studies, and identified the key research trends (i.e., focusing on the earth‐abundant materials to achieve high performances). Interestingly, the link strength for the co‐occurrence network of keywords, contributing countries, and focal area in the VOSviewer software was evaluated. Authors also offered insight into the emerging Al and policies for photoanode and PEC devices, such as strategies for improving device performances. Medina et al. investigated the bibliometric examinations and state‐of‐the‐art overview of hydrogen evolution from PEC devices using 936 articles issued in the Web of Science database from 1970 to 2022 [355]. Authors demonstrated how PEC water splitting research has developed compared to prior research, what criteria apply to practical approaches, and what factors have emerged as new research trends. After bibliometric analysis, the world annual growth rate, trends of publication, citations, interactions among the most relevant countries, sources, and keywords, collaboration map from co‐authors, countries, citations, and keywords, and link strength for each keyword were visualized and discussed for each factor. Recently, Farrusseng and co‐workers reviewed the AI‐driven advances in photocatalytic hydrogen production, including the synthesis process, efficiency, harvesting sunlight, design of the reactor, and interaction between photon, solutions, photocatalysts, and co‐catalysts [356]. In particular, authors revealed the co‐occurrence network with all keywords using the VOSviewer software, shown in Figure 21b. Based on bibliometric analysis, authors suggested future research directions, including the use of green materials and the process of synthesis, avoiding extensive experimentation to reduce the carbon footprint and seek efficient and less resource‐intensive computational resources.

### 6.2 AI Approach for Discovery of Materials and Deposition Methods

It is well known that the timelines for new materials discovery, development, and applications into devices are long, and the related process is capital‐intensive because the developed new materials reach the commercial stage after 10–20 years [3]. ML and/or AI‐derived approaches have the potential to accelerate the entire materials discovery and innovation process to reduce the research period by an order of magnitude. To efficiently discover new materials for photoelectrode and/or photocatalysts, the selection of domain factors including optoelectrical properties (i.e., E_g_, position of VBM and CBM, and charge transfer), toxicity, and low cost in composed elements, theoretical calculations are essential. In this regard, Neaton and co‐workers developed a meaningful photoanode material discovery by integrating high‐throughput theory and experimental results [357]. Authors found eight ternary vanadate oxide photoanodes ranging from 1.2 to 2.8 eV of E_g_ by integrating database mining (i.e., M‐V‐O with VO_4_ motif), computational screening (i.e., PBE+U and HSE), and a high‐throughput experimental screening process (i.e., crystal purity, E_g,_ and photocurrent density). This screening‐related approach is very effective and is considered an effective research method for new materials discovery and the synthesis process. Similarly, Jacobsen and co‐workers demonstrated the screen pipeline to identify the photoelectrode materials for PEC devices from a previously synthesized semiconductor database using toxicity, element abundance, stability, high charge carrier mobility, E_g,_ and defect tolerance of materials [358]. Authors found 74 materials with pnictogenides, chalcogenides, and halides from the 22,807 material database. Initial screening of toxicity and abundance in the composed elements filtered out the candidate materials from 22,807 to 7,241. Second screening of E_g_ was calculated using the open quantum materials database, and they reduced the candidate materials to 1630. The detailed calculated process and prediction of E_g_ reduced the candidate materials to 518 (i.e., wide E_g_: 323 and narrow E_g_: 195). Third screening of charge carrier transport and effective mass could be considered as candidate materials of 222. Finally, the filtering defect‐related properties (i.e., point defects and related defect clusters) and DOS study can be derived for the candidate materials of 74. Around the same time, Ertekin and co‐workers designed the functionalized Si photocathode materials and photocatalysis (i.e., a set of 20 mixed aryl and methyl monolayers) by integrating a computational approach combining DFT calculations and a solid‐state drift and diffusion device model (i.e., wxAMPS) [359]. As a result, authors offered therelationship between the molecular dipole moments of different aryl and methyl monolayers and the corresponding DFT calculated surface dipole with respect to Si‐H bonding. Based on these investigations, the combination of functionalized Si photocathode materials and photocatalysis for a small barrier for charge transfer and large photovoltage was discovered. Recently, Caruso et al. reviewed the electrocatalyst and photocatalyst design and materials discovery using ML approaches [31]. This review offered the information for critical ML techniques, the process of implementing ML techniques for electro/photocatalyst systems, including a summary of ML algorithms and their merits and limitations, literature on ML‐based electro/photocatalyst design, discovery, and development for materials, and a perspective on current challenges and future development.

### 6.3 Prediction of Device Performance

**FIGURE 22:**
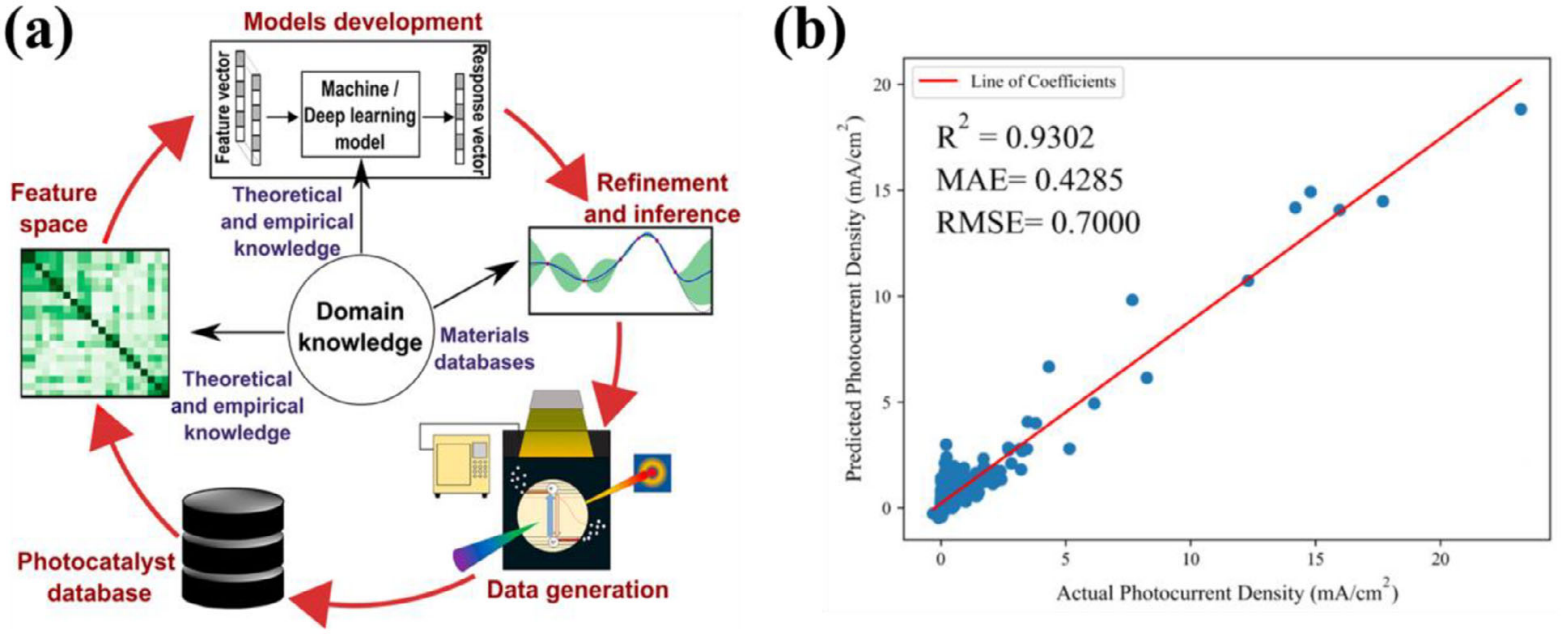
(a) Integration of domain knowledge into ML frameworks for photoelectrode and/or photocatalyst materials discovery. Reproduced with permission [360]. Copyright 2019, American Chemical Society. (b) Correlation graph between actual and predicted photocurrent density. Reproduced with permission [36]. Copyright 2024, Elsevier.

Another merit of ML and/or AI‐derived approaches can offer significantly improved device performance and long‐term stability in PEC systems through comparative analysis between predictive and experimental results and feedback to the experimental process in a short research period. In this regard, Amal and co‐workers successfully demonstrated the integration of domain knowledge into ML frameworks for photoelectrode and/or photocatalyst materials discovery and predictions for performances and/or long‐term stability shown in Figure 22a [360]. Authors classified the domain knowledge into (i) theoretical knowledge and empirical knowledge derived from first‐principle and experimental observations, and (ii) knowledge from existing materials databases. Based on this classification, the ML‐derived strategies proposed like (i) integration of theoretical and prior empirical knowledge during training of the ML model, (ii) embedding the knowledge in feature space, and (iii) utilizing existing material databases to constrain ML predictions. Jiang and co‐workers utilized the comprehensive ML process to guide and assist in the analysis and preparation of copper‐based sulfide photocathodes [29]. Through data preprocessing techniques (i.e., data cleaning, imputation, and partitioning), the valid data set for the ML model was secured. Authors investigated the random forest model was selected to train and capture the complex relationship between different layers of photoelectrodes and electrolytes to predict unrevealed conditions, and the accuracy of the test set reached 96.7%. Similarly, Wu and co‐workers demonstrated the combination of ML and first principles to screen effectively for double perovskite oxides for photocatalytic water splitting [361]. Authors collected over 2500 double perovskite oxides from multiple databases. The E_g_ of candidate materials was predicted using a two‐step modeling method. As a result, the predicted materials with high performance were classified as nearly 8000 from 56 984. On the other hand, Kumar and co‐workers constructed the prepared hybrid models (i.e., MLP, adaboost, Ridge, and Elastic Net) based on an ML approach to predict the PEC device performances (see Figure 22b) [36]. Authors suggested the ML played a critical role in determining the device performance through predicting photocurrent density. The device performance of the photoelectrode using the three‐hybrid model was better than each individual, and the accuracy between the prediction and experimental result was equal to 96.89%. Recently, Katayama et al. demonstrated insight into charge transport behaviors in Fe_2_O_3_ photoanode using a combination strategy of potential‐dependent impedance spectroscopy (PEIS) characterization and ML [35]. Through the Shapley additive explanation analysis from the ML model using PEIS and PEC performance, authors suggested the main factors for electron transport at the back contact in bulk and hole transfer at the photoelectrode/electrolyte interface. As a result, the shallow defect states significantly enhanced the electron transfer, while deep defect states contributed to the hole transfer. Introduction of TiO_2_ underlayer and CoP_i_ cocatalyst improved the charge separation and hole transport, leading to the enhancement of the overall device performance.

### 6.4 Automation of Experiment Planning, Execution, and Analysis

**FIGURE 23:**
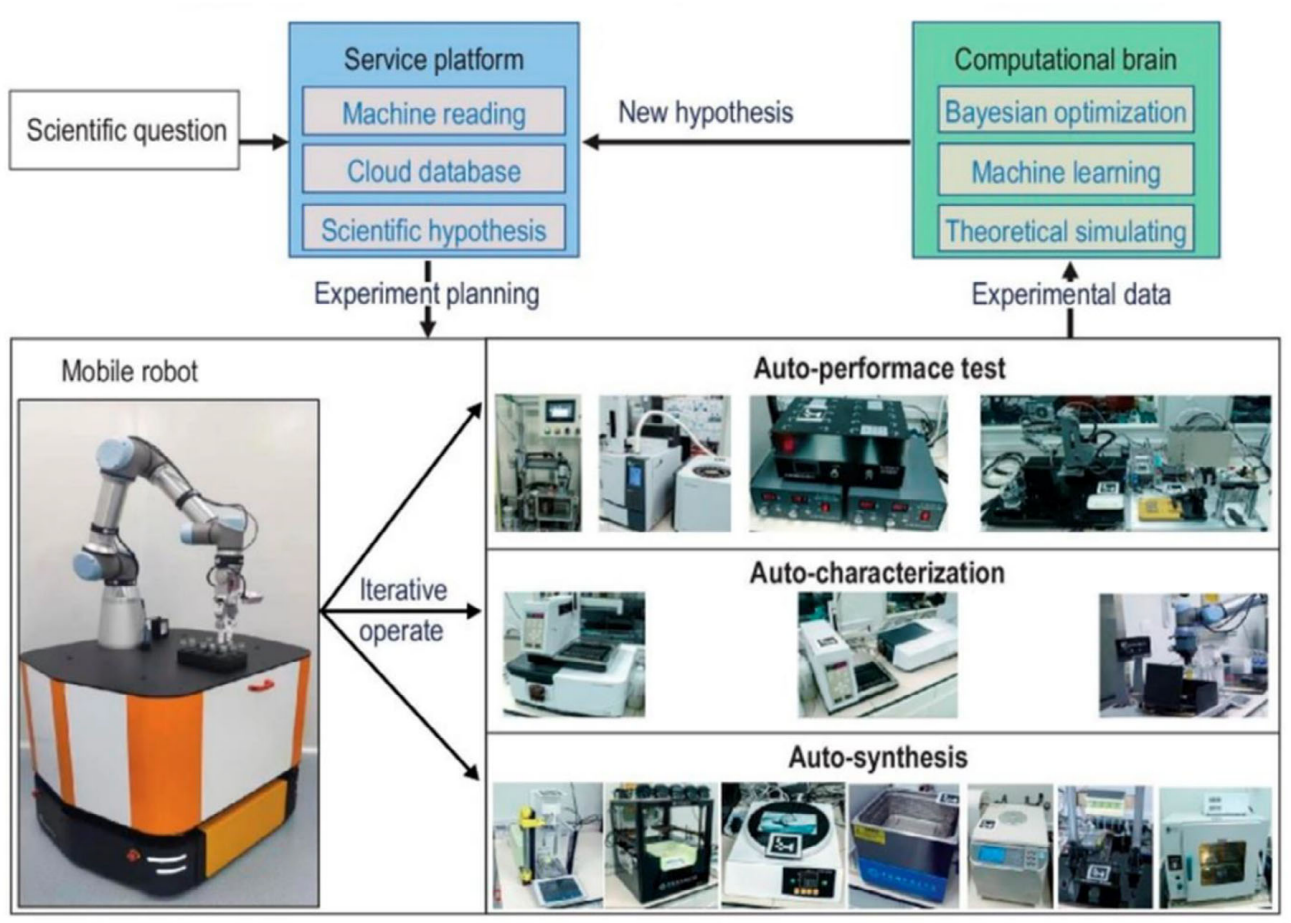
The workflow of the Al‐chemist and the functions of each module. Reproduced with permission [370]. Copyright 2022, Oxford University Press.

As mentioned previously, the lack of diverse data and the need to be able to test it experimentally clearly contribute to the high accuracy and scope of the predictive models derived from ML‐ and AI‐related data science. In this regard, integrating automated experimentation, ML, and a data‐rich monitoring approach can improve the performance and long‐term stability of PEC devices in a short period. It was pioneered by the pharmaceutical research field in the search for efficient screening of new materials and for cost‐effective synthesis of new organic compounds [362, 363]. The automated synthesis process should require the fine control of chemical reaction and in‐suit characterization tools of the products to be linked through feedback loops at the hardware level, and they still remain as challenges [21]. In this regard, several automation strategies have been developed, like flow chemistry, microfluidic systems, and nanomole‐scale batch miniaturization, and a meaningful automated synthesis system was developed [364, 365, 366, 367, 368, 369]. Interestingly, Luo and co‐workers have built an all‐round AI‐chemical synthesis and characterization facility [370]. It can (i) propose scientific hypotheses and conduct experiment plans using previously reported results, (ii) execute the experimental procedure (i.e., synthesis, characterization, and performance measurement), and (iii) build predictive models exploiting theoretical computations and experimental results feedback shown in Figure 23.

Computing strategy has emerged to achieve it efficiently to discover high performance of photoelectrodes and photocatalysts, and to fully understand the complex structure‐properties relationship. As mentioned above, it offered a unique opportunity for the selection of critical factors, material discovery for new photoelectrodes and photocatalysts, predicting input‐output correlations for improving device performance, and automated experiments and characterizations in a complex device system. However, several questions and/or challenges remain as follows.

- Classifying valid experimental data from a web‐based database, selecting an appropriate database size, and standardizing the database to obtain high‐accuracy ML or AI models.
- High sensitivity between experimental parameters (i.e., temperature, pressure, compositional ratio, annealing atmospheres, and electrolyte conditions) and device performances.
- Difficulty for multi‐step and complex reactions of PECs in applying ML or AI models.

## 7 Conclusions and Future Prospects

**FIGURE 24:**
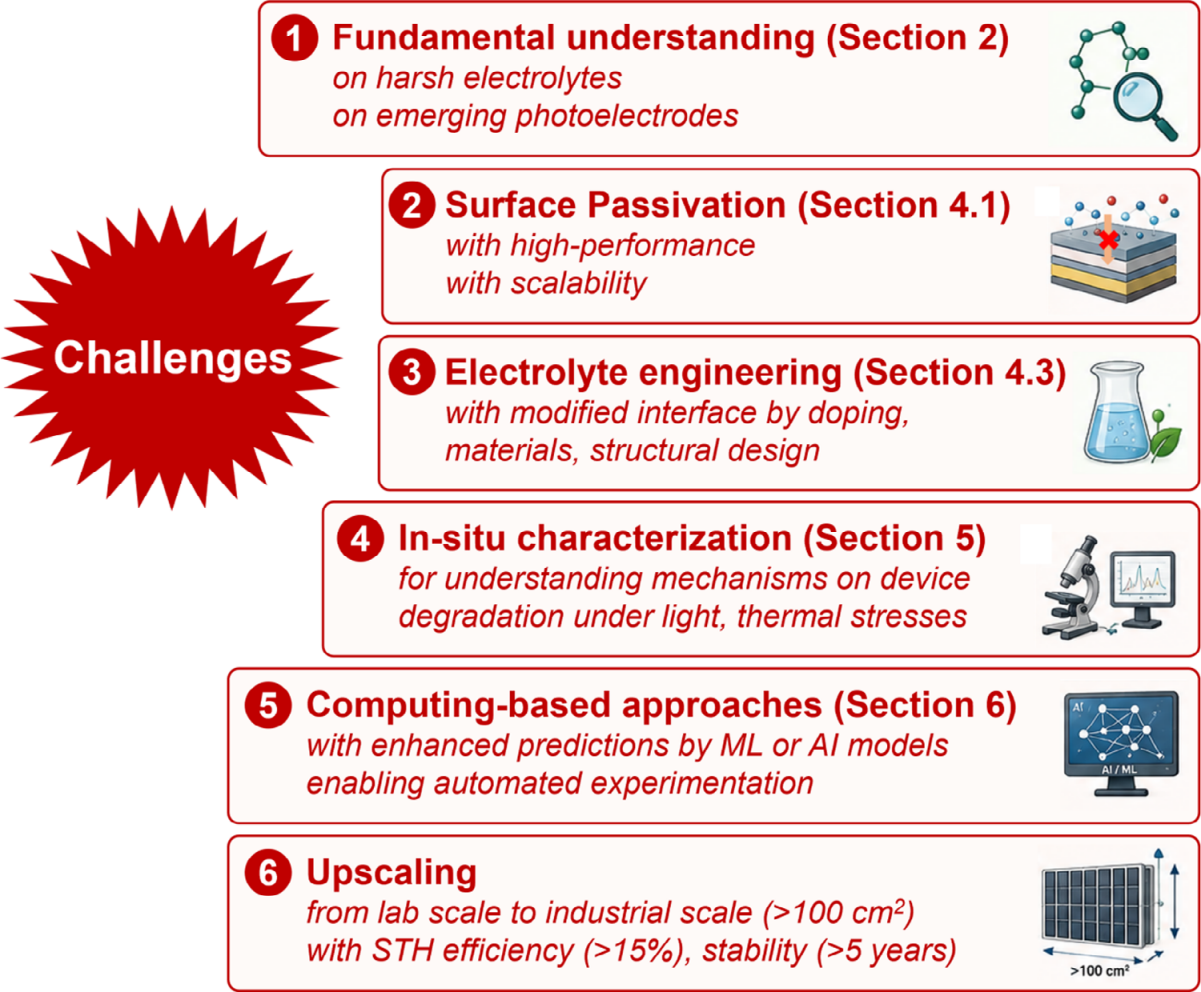
Key challenges remaining for the commercialization of PEC water‐splitting devices based on inorganic photoelectrodes.

This review provides a concise overview of the origins of instability, including photocorrosion, the current performance of the state‐of‐the‐art devices, and in‐depth discussions on the merits and drawbacks of inorganic photoelectrodes (e.g., Si, III‐V, Cu_2_O, kesterite, Sb_2_Se_3_, TiO_2_, Fe_2_O_3_, and BiVO_4_) in PEC water‐splitting devices. This review introduces recently advanced strategies, the application of passivation layers (metal, metal oxide, MIS, or amorphous materials); interfacial engineering between catalysts and passivation layers aiming at improving adhesion, introducing multifunctional materials, and/or adjusting band alignment); mitigation of undesired chemical dissolution during PEC operations (through electrolyte modification, in situ catalyst formation, or neutral electrolytes); and exploration of damage‐free deposition processes (solution‐based approaches, ALD, CVD, and MBE). In addition, this review comprehensively discusses recently advanced strategies and characterization tools concerning photoexcitation and reaction dynamics, as well as PEC processes across timescales ranging from picoseconds to hours. Finally, computing‐based strategies, applicable across the entire PEC device development cycle, include identifying critical factors by bibliometric analysis, discovering new materials, predicting device performance, and automating experiments. Expanding on the aforementioned key fundamentals, materials, and strategies, we highlight key findings and future prospects for the realization of early commercialization of PEC water‐splitting devices based on inorganic photoelectrodes, as summarized below and in Figure 24.

1. The origin of instability and photocorrosion mechanism for known photoelectrodes has been extensively investigated (Section 2); however, their theoretical understanding and detailed studies on the dissolution process under harsh electrolyte environments (i.e., strong acidic, alkaline, and seawater solutions). This should include the detailed interaction resistance for the dissolution process between specific atoms and the photoelectrode, as well as how to suppress the dissolution process between defects and electrolyte by adjusting doping or defect engineering strategies in harsh electrolyte conditions. Furthermore, the theoretical, fundamental, and detailed photocorrosion mechanism‐related investigations for emerging photoelectrode materials (i.e., Bi‐ and Sb‐based chalcohalides) are essential to unlock their potential for highly efficient and durable PEC water splitting device applications.
2. Metal, metal oxide, MIS, and amorphous materials‐based protective layers on photoelectrodes (Section 4.1) have been extensively introduced to achieve excellent performance and long‐term stability for earth‐abundant‐based photoelectrode‐based PEC water‐splitting devices. To achieve early industrialization, new protective materials prepared by a low and/or no‐damage deposition process should be mainly developed, in collective consideration of high STH efficiency, durability, and scalability. For example, the carbon‐based materials (i.e., graphitic carbon nitride, graphene derivatives, and carbon quantum dots) can be employed to preserve or enhance PEC performances as well as long‐term stability in an acidic electrolyte environment. However, they still face some challenges, like scalability and parasitic light absorption.
3. The conditions for electrolyte (i.e., pH, compositional ratio, and additive) are essential factors for secure long‐term stability and achieving early industrialization in earth‐abundant‐based photoelectrodes for PEC water‐splitting application (Section 4.3). Particularly, it is significantly improved by neutral electrolytes owing to their less corrosive nature; however, the produced photocurrent densities have not been greatly improved compared to those under strong acidic or alkaline conditions. To solve this issue, the known (i.e., doping, surface modification, heterojunction, co‐catalysts, seed layer, and morphology control) and novel strategies (i.e., plasmonic nanostructure, conjugated polymers, and surface densification) to improve photocurrent density should be developed and introduced simultaneously.
4. The advanced characterization tools for PEC operations are widely investigated. It is crucial to develop in situ characterization tools for performance degradation mechanisms in the excellent long‐term stability of PEC water splitting devices. As discussed in Section 5, the currently developed characterization tools (i.e., Raman, XAS, IR spectroscopy, and SPM) are not sufficient to fully understand the performance degradation mechanism, and they only focus on in situ electrocatalysis reactions, not PEC operation. Unlike other optoelectronic applications (i.e., solar cells, photodetectors, photodiodes, and light‐emitting diodes), the PEC operation is quite light and/or thermal sensitive. Therefore, the low energy‐utilizing in situ characterization tools should be developed and widely investigated to reveal the PEC performance degradation for various photoelectrodes. These characterization tools may contribute to discovering the nature of dissolution between the specific site and electrolyte during PEC operation and facilitate a full understanding of PEC performance degradation and establish an excellent strategy.
5. Computing‐based approaches for collected literature and experimental results‐driven analysis can significantly contribute to the development of novel photoelectrodes and photocatalysts and improvement of device performance and long‐term stability in a short period (Section 6). However, the large gap between experimental results and predictions for device parameters and performances should be reduced to significantly enhance the PEC water splitting devices at the commercial level. Securing valid experimental data, revealing highly sensitive experimental parameters for device performances and long‐term stability, and demonstrating input data for multi‐step and complex PEC reactions for PEC should be solved to achieve excellent ML or AI models. Furthermore, the automated experimentation framework needs to be advanced on the ML or AI models.
6. The scalability is also a crucial issue to achieve early industrialization in earth‐abundant‐based photoelectrodes for PEC water‐splitting application. As discussed, strategies, most of the research has focused on lab‐scale devices and long‐term stability characterizations. There are a few studies for large‐scale PEC water splitting devices over 100 cm^2^ with excellent long‐term stability. Achieving ≥10% STH efficiency serves as a key performance indicator for the practical viability of PEC research, however, to make PEC water‐splitting devices competitive for practical deployment, systems should target STH efficiencies of at least 15% together with operational stability on the order of 5 years or longer, which underscores the need to develop robust protective strategies in parallel with device scale‐up.

## Conflicts of Interest

The authors declare no conflicts of interest.

## Data Availability

The data that support the findings of this study are available from the corresponding author upon reasonable request.
